# Mitigating Dexamethasone‐Induced Muscle Wasting and Mitochondrial Impairment in Mice on a High‐Fat and High‐Sucrose Diet With Peanut Sprout Extract

**DOI:** 10.1002/fsn3.71469

**Published:** 2026-01-19

**Authors:** Sang‐Mi Jo, Thi My Tien Truong, Hyun‐Jin Jang, Ji Hee Lim, Inhae Kang

**Affiliations:** ^1^ Department of Food Science and Nutrition Jeju National University Jeju Korea; ^2^ Interdisciplinary Graduate Program in Advanced Convergence Technology and Science Jeju National University Jeju Korea; ^3^ OLAOLAB Inc Jeju Korea; ^4^ Center for Nutrition‐Disease Research & Intervention Systems Jeju National University Jeju Korea

**Keywords:** dexamethasone, high‐fat and high‐sucrose diet, inflammation, mitochondrial dysfunction, muscle atrophy, peanut sprout extract

## Abstract

Prolonged high‐fat and high‐sucrose diets (HFHS) diet accelerates skeletal muscle atrophy and impairs muscle function. Combined HFHS diet and dexamethasone (Dex), a synthetic glucocorticoid which involves the induction of protein degradation, will promote atrophy of skeletal muscle. Although we previously demonstrated that peanut sprout extract (PSE) inhibits adipogenesis, its impact on HFHS+Dex‐induced muscle atrophy remained unknown. To investigate, we treated C57BL/6 male mice with a control or HFHS diet, with or without PSE (10 mg/kg BW), over 10 weeks, introducing Dex (10 mg/kg BW) once daily for six consecutive days to induce muscle atrophy. PSE treatment reduced skeletal muscle triglyceride accumulation, restored muscle strength (grip and hanging capacity), and mitigated muscle atrophy expression. While systemic interleukin (IL)‐1β levels were unaffected, PSE reduced inflammatory gene expression and inhibited nuclear factor‐κB (NF‐κB) protein expression in skeletal muscle, enhanced mitochondrial function (increased mitochondrial transcription factor A (TFAM), oxidative phosphorylation (OXPHOS) complex IV/V protein expression, but no differences in peroxisome proliferator‐activated receptor gamma coactivator 1‐alpha (PGC1α)). Consistent with these results, PSE protected against muscle atrophy in Dex‐treated C2C12 cells by modulating atrophic and inflammatory expression. This study highlights PSE's efficacy in attenuating skeletal muscle atrophy and mitigating inflammation with partial enhancement of mitochondrial function.

## Graphical abstract

Peanut sprout extract (PSE) significantly attenuated skeletal muscle atrophy induced by a high‐fat, high‐sucrose diet and dexamethasone, improving muscle strength and reducing triglyceride accumulation and inflammation. It enhanced mitochondrial function by upregulating TFAM and OXPHOS proteins and partially restoring PGC1α expression. Additionally, PSE protected C2C12 myotubes from dexamethasone‐induced atrophy and inflammation in vitro, highlighting its potential as a nutraceutical intervention for muscle wasting.

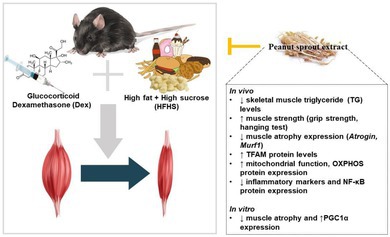

**Abbreviations**
36B4/RPLP0: Ribosomal protein lateral stalk subunit P0
Atrogin‐1/MAFbx: Muscle atrophy F‐box
AWGS2: Asian Working Group for Sarcopenia
BW: Body weight
BWG: BW gain
Dex: Dexamethasone
EWGSOP2: European Working Group on Sarcopenia in Older People
GAPDH: Glyceraldehyde‐3‐phosphate dehydrogenase
HFHS: high‐fat and high‐sucrose diet
HPRT: Hypoxanthine–guanine phosphoribosyltransferase
IL1β: Interleukin‐1 beta
IL6: Interleukin‐6
LPS: Lipopolysaccharide
MuRF1: Muscle RING‐finger protein‐1
Myod1: Myogenic Differentiation 1
NFκB: nuclear factor‐κB
OXPHOS: Oxidative phosphorylation
PGC1α: Peroxisome proliferator‐activated receptor‐γ coactivator1‐α
PS: peanut sprout
PSE: peanut sprout extract
SDOC: American Society for Bone and Mineral Research;TC: Total cholesterol
TFAM: mitochondrial transcription factor A
TG: Triglyceride
TNFα: tumor necrosis factor‐α

## 1 Introduction

Skeletal muscle, comprising 40%–50% of body mass, is essential for movements, metabolic regulation, and whole‐body protein homeostasis (Frontera and Ochala 2015; Lee et al. 2022; Yadav et al. 2022). Muscle atrophy is triggered by various factors, including aging, sedentary lifestyle, malnutrition, and pharmacological agents such as glucocorticoids (GCs). This reduction in muscle mass and function leads to increased fall risk, susceptibility to fractures, and higher mortality (Argiles et al. 2016; Chen et al. 2020; Fanzani et al. 2012). Mechanistically, mitochondrial dysfunction is a central driver of atrophy, disrupting cellular homeostasis and ATP production, which in turn suppresses protein synthesis and accelerates proteolysis (Chen et al. 2023; Hyatt et al. 2019; Peng et al. 2021; Romanello and Sandri 2021; Chen et al. 2020; Cruz‐Jentoft et al. 2019; Lee et al. 2022; Stuck et al. 2023). Thus, finding out anti‐atrophic bioactive components modulating mitochondrial dysfunction is a promising area for human well‐being health.

Concurrently, the consumption of high‐fat and high‐sucrose (HFHS) diets contributes to obesity, a condition often complicated by myosteatosis—the ectopic accumulation of fat within skeletal muscle (Han et al. 2017; Amini et al. 2019; Tachi et al. 2018). Myosteatosis exacerbates muscle weakness and metabolic dysfunction. In clinical settings, synthetic GCs like dexamethasone (Dex) are widely used for their anti‐inflammatory effects, yet they induce rapid and severe muscle atrophy by stimulating protein degradation and mobilizing amino acids for hepatic gluconeogenesis (Son et al. 2015). Recent evidence suggests that when GC therapy is combined with obesity, the metabolic burden is compounded, accelerating both muscle loss and insulin resistance (Gounarides et al. 2008; Savas et al. 2017; Alev et al. 2022; Han et al. 2017; Son et al. 2015). In obese individuals, the overuse of glucocorticoids can exacerbate these effects, promoting both muscle atrophy and myosteatosis, thereby compounding metabolic and functional impairments (Savas et al. 2017). Understanding the interaction between dietary stress and GC‐induced atrophy is therefore critical for developing effective therapeutic strategies.

Peanuts are rich in diverse bioactive compounds, such as resveratrol, flavonoids, phenolic compounds, and phytosterols (Choi et al. 2015). The germination of peanut sprouts results in a significant increase in the concentration of resveratrol, which is well known for its anti‐disease effects, such as cancer, cognition, and metabolic syndromes (Wang et al. 2005). Additionally, these sprouts exhibit elevated levels of sucrose, glucose, and total free amino acids (Wang et al. 2005). Peanut sprout extract (PSE) has been reported to exhibit anti‐inflammatory (Choi et al. 2015; Lee et al. 2019) and antioxidant properties (Choi et al. 2013; Wu et al. 2011). Moreover, it offers various health benefits, including relieving constipation (Seo et al. 2013), improving prostate function (Pyo et al. 2017; Song et al. 2020), enhancing alcohol metabolism (Seo et al. 2014), and inhibiting adipocyte proliferation and differentiation (Kim et al. 2013).

In our previous reports, we reported that PSE inhibits lipid accumulation in adipocytes with enhancements of mitochondrial fatty acid oxidation (Seo et al. 2019). Furthermore, it effectively mitigates high‐fat (HF) diet‐induced inflammation, activates mitochondria, reduces lipopolysaccharide (LPS)‐induced inflammation, and prevents the suppression of adipocyte browning (Seo et al. 2021). We further identified *p*‐Coumaric acid (PCA) as a prominent constituent that partially recapitulates the activity of PSE (Seo et al. 2021); PCA mitigated muscle atrophy associated with HFHS feeding (Truong et al. 2024). However, the effects of PSE on skeletal muscle atrophy have not been fully defined. Here, we examined whether PSE attenuates dexamethasone‐induced skeletal muscle atrophy under dietary stress. The mice were allocated to Control, Dex + HFHS, and Dex + HFHS + PSE groups to model the interaction between dietary metabolic overload and glucocorticoid‐induced catabolic stress on skeletal muscle. However, a Dex‐only group (with or without PSE) was not included in the experimental design, which limits the ability to distinguish the independent effects of glucocorticoid signaling from those arising from the combined Dex + HFHS exposure. Therefore, the present data should be interpreted as reflecting the integrated impact of glucocorticoid treatment superimposed on HFHS feeding, rather than the isolated actions of Dex alone. Then, we utilized complementary experiments in Dex‐treated C2C12 myotubes in vitro.

## 2 Materials and Methods

### 2.1 Sample Preparation and Experimental Materials

Dulbecco's Modified Eagle's Medium (DMEM), penicillin/streptomycin (P/S), horse serum (HS), and fetal bovine serum (FBS) were sourced from Gibco (Fabulous Island, NY, USA), whereas other chemicals and reagents were supplied by Sigma Aldrich Compound Co. (St. Louis, MO, USA). Unless otherwise specified, all cell culture dishes were procured from SPL Life Sciences (Seoul, Korea).

PSE was prepared according to procedures outlined in previous studies (Seo et al. 2019). Peanut sprouts were provided by Olaolab Inc. (Jeju, South Korea) that germinated for 9 days, were first dried and freeze‐dried to obtain a powdered form. To prepare the extract, dried PS powder (10 g) was mixed with water (100 mL) and subjected to pressurized hot‐water extraction as previously described (Seo et al. 2019). The resulting extract was centrifuged (3000 rpm, 3 min) and filtered using Whatman filter paper. Subsequently, the filtrate was lyophilized to obtain a powdered extract. These samples were dissolved in dimethyl sulfoxide (DMSO, Sigma) at a concentration of 100 mg/mL for use in both cell and animal experiments. The detailed composition of these samples has been previously reported in our earlier publication, and the same lyophilized batch of PSE prepared and characterized in that study was used in the present work (Seo et al. 2019).

### 2.2 Animals and Treatments

All animal experiments and experimental strategies were approved by the Institutional Creature Care and Use Committee of the Jeju National University (IACUC ID # 2021–0026). All animal experiments and husbandry have been carried out under the guidelines of the Jeju National University IACUC. All methods are reported in accordance with ARRIVE guidelines. Six‐week‐old C57BL/6 mice were purchased from the ORIENT BIO Animal Center (Seongnam‐si, South Korea) and housed under 12‐h dark/light cycles with free access to food and water. Prior to the study, the mice underwent a 2‐week adaptation period with oral gavage. For 10 weeks, eight‐week‐old male C57BL/6 mice were fed either a control diet (Control) or an HFHS diet with either saline or PSE (10 mg/kg body weight [BW], oral gavage). The concentration of PSE was based on previous study and bioavailability results (Sales and Resurreccion 2014; Seo et al. 2021). During the last 6 days, Dex (10 mg/kg BW) was administered once daily for six consecutive days through intraperitoneal injection to exacerbate muscle atrophy in mice, resulting in three groups: (i) control (*n* = 5), (ii) Dex + HFHS (*n* = 8), and (iii) Dex + HFHS+PSE (*n* = 9). Control diet (Control, 11% kcal fat) and HF diets (60% kcal fat) were prepared based on modifications of the AIN‐93G diet (Table S1). High‐sucrose water (0.8 kcal/g of HS drinking water) was prepared by autoclaving 20% sucrose in deionization water at 115°C for 15 min. Body weight and food intake were measured weekly during the experiment. Upon completion of the study, the tissues were promptly excised, snap‐frozen, and stored at −80°C until subsequent analysis.

### 2.3 Measure Serum Biochemistry Parameters

After completing the experiment, the animals were fasted for 12 h and euthanized via carbon dioxide narcosis. Blood was collected via cardiac puncture, and serum samples were aliquoted. The serum total cholesterol (TC, mg/dL) and triglyceride (TG, mg/dL) levels were analyzed using an enzyme assay kit (Asan Pharmaceutical Co., Seoul, Korea) as per the manufacturer's protocol with absorbance at the wavelengths of 500 and 550 nm, respectively. Blood glucose levels were measured using a glucose meter (Contour Next, Bayer NJ, USA). Insulin levels in the mouse serum were measured using an ultra‐sensitive mouse insulin ELISA kit according to the manufacturer's protocol (Crystal Chem, Elk Grove Village, IL 60007, USA). The homeostatic model assessment for insulin resistance (HOMA‐IR) was calculated as [(fasting plasma glucose) × (fasting plasma insulin)]/22.5 (mmol/L). Plasma IL‐1β levels were measured using a mouse IL‐1 beta/IL‐1F2 Quantikine ELISA Kit (MLB00C, R&D Systems Inc., Minneapolis, MN 55413, USA).

### 2.4 Hematoxylin and Eosin (H&E) Staining and Muscle Fiber Size Quantification

Upon necropsy, quadricep muscles, epididymal adipose tissue, and hepatic tissue of the mice were promptly excised and fixed in formalin buffer (10%). These specimens were subsequently embedded in paraffin, and 5–7‐μm thick tissue sections were prepared. The sections were stained with hematoxylin and eosin (H&E). Bright‐field images of the stained sections were captured using an Invitrogen microscope (Invitrogen EVOS FL Advanced Rearranged Fluorescence Magnifying instrument, Invitrogen, CA, USA) at 10X and 20X magnifications. Adipocyte size was examined using ImageJ (NIH, USA). The muscle fiber size of quadriceps muscle tissues and hepatic steatosis were quantified using the Materials Image Processing and Automated Reconstruction (MIPAR) software (MIPARTM, Worthington, OH, USA) and ImageJ.

### 2.5 Muscle Lipid Accumulation

For the extraction of total muscle lipids, gastrocnemius muscle tissue weighing 100–150 mg was subjected to methanol and chloroform mixture (1:2 v/v) treatment at a 20‐fold dilution. The resulting mixture was thoroughly blended and incubated at 60°C for 3 h, followed by an overnight incubation at room temperature (RT). Subsequently, the samples were filtered using Whatman filter paper, subjected to multiple chloroform washes, and air dried at RT. Once dried, the samples were reconstituted in deionized water. The quantification of triglyceride (TG) content in the muscle samples was quantified using a TG enzyme assay kit sourced from Asan Pharma Co. Ltd. (Seoul, Korea), and absorbance was measured at 550 nm.

### 2.6 Muscle Strength Test

During the 10th week of the experiment, grip strength was assessed using a Grip Strength Meter (Ugo‐Basile). Forelimb grip strength primarily reflects upper‐body muscle function, involving the brachialis, biceps brachii, and flexor muscles, while hind‐limb grip strength indicates lower‐body muscle function, including the gastrocnemius, tibialis anterior, and soleus muscles (Alford et al. 1987; Takeshita et al. 2017). Forelimb grip strength was measured thrice, and the highest recorded value among the three was considered to represent the subject's true maximal muscle capacity. Similarly, for the forelimb and hindlimb grip strength assessment, the highest recorded value among the three was considered. Grip strength and hanging capacity were normalized by muscle weight (combined weight of the quadriceps and gastrocnemius muscles). The hanging test assessed the hanging duration of the mice to a wire mesh after they were allowed to hold it by themselves before it was inverted. Each mouse underwent a minimum of three assessments, and the longest duration was considered.

### 2.7 Cell Culture

C2C12 myoblast cells from the American Type Culture Collection (ATCC) (Manassas, VA, USA) were cultured in basal medium consisting of DMEM supplemented with 10% FBS and 1% P/S. Upon reaching 80%–90% confluence, the growth medium was substituted with a differential medium containing 2% horse serum (HS) in DMEM supplemented with 1% P/S to induce myotube differentiation. Cell cultures were maintained for 4–6 days, with the medium changed every other day. C2C12 cells were treated with Dex (10 μM) and PSE (25 μg/mL) for 24 h.

### 2.8 Cell Viability Assay

The cytotoxic effect of PSE on C2C12 cells was assessed using the EZ‐cytox cell viability assay kit (DAEIL LAB SERVICE CO. Ltd., Seoul, Korea) following the manufacturer's instructions. C2C12 cells were seeded in a 96‐well plate at a density of approximately 20,000 cells/well in the growth medium. Subsequently, the cells were treated with either DMSO or PSE (25 μg/mL) for 24 h. Following treatment, the growth medium was replaced with fresh medium containing 10% EZ‐cytox solution, and the cells were incubated at 37°C. The microplate reader measured the plate at OD 450 nm for a duration of 0.5–4 h to determine the cell viability.

### 2.9 Analysis of mRNA by Real‐Time Polymerase Chain Reaction (Real Time‐PCR)

RNA was extracted from both gastrocnemius muscle tissues and cells using TRIzol reagent (Invitrogen). Prior to cDNA synthesis, RNA concentrations were determined using a Nano‐200 Micro‐Spectrophotometer (Hangzhou City, China). The cDNA synthesis was conducted using the iScript cDNA synthesis kit (Bio‐Rad, Hercules, CA, USA). For gene expression analysis, real‐time quantitative PCR (qPCR) was performed using a CFX96 Touch Real‐Time PCR Detection System (Bio‐Rad, CA, USA). Hypoxanthine‐guanine phosphoribosyltransferase (HPRT) and/or ribosomal protein lateral stalk subunit P0 (RPLP0/36B4) served as reference genes for normalizing the relative gene expression. Gene‐specific qPCR primers were sourced from Cosmo Genetech (Seoul, Korea) (Table S2).

### 2.10 Protein Isolation and Western Blotting

Gastrocnemius muscle tissues (mid‐belly portion) were homogenized in ice‐cold radioimmunoprecipitation (RIPA) lysis buffer supplemented with a protease and phosphatase inhibitor cocktail. The homogenized lysates were subsequently centrifuged (10,000 rpm, 4°C, 10 min) to separate the supernatant. For protein analysis, 10–15 μg of protein was loaded onto an 8%–10% SDS‐PAGE gel. The proteins were separated through electrophoresis and then transferred to a polyvinylidene difluoride (PVDF) membrane (Thermo Fisher Scientific, MA, USA) using the transfer buffer, Tris‐buffered saline/Tween 20 (TBST). Subsequently, the membrane was blocked with a blocking solution for 1 h at RT. Following the blocking step, the PVDF membranes were washed multiple times with TBST solution. Subsequently, they were incubated overnight at 4°C with primary antibodies against Muscle atrophy F‐box (MAFbx/Atrogin‐1), Muscle specific RING finger protein (MuRF1) (Santa Cruz Biotechnology, CA, USA); glyceraldehyde‐3‐phosphate dehydrogenase (GAPDH); oxidative phosphorylation (OXPHOS) (Abcam, Cambridge, MA, USA); peroxisome proliferator‐activated receptor gamma coactivator 1‐alpha (PGC1α) (Novus Biologicals LLC); Mitochondrial transcription factor A (TFAM) (Abcam, Cambridge, MA, USA), and total or phospho nuclear factor‐κB (NF‐κB) (Cell Signaling Technology, Danvers, MA, USA). The following day, the membranes were washed several times with 1X TBST and incubated with a secondary antibody, either goat anti‐rabbit (Cell Signaling Technology, Danvers, MA, USA) or goat anti‐mouse IgG‐horseradish peroxidase (HRP) (Santa Cruz Biotechnology), for 1 h. After washing, the membranes were treated with an enhanced chemiluminescence (ECL) reagent, specifically the SuperSignal West Pico PLUS Chemiluminescent kit (Thermo Fisher Scientific). Protein bands were visualized using a ChemiDoc MP system (Bio‐Rad). The original blots, showcasing full‐length gels and membrane edges, are provided in Figure S2. However, some full‐length blot images are not included due to hybridization with additional antibodies. Their expression levels were determined using the Image Lab software (Bio‐Rad) or ImageJ software (National Institutes of Health [NIH], MD, USA).

### 2.11 Oxygen Consumption Rate by Seahorse Extracellular Flux Analyzer

The oxygen consumption rate (OCR) in the C2C12 cells was measured using a XF24 extracellular flux analyzer (Agilent Technologies, Santa Clara, CA, USA/Bio‐Health Materials Core‐Facility at Jeju National University) for measuring the mitochondrial respiration activities as previously described (Seo et al. 2021). C2C12 cells were seeded in an XFe analyzer microplate (24‐well) and cultured until they reached confluency. C2C12 cells were treated with PSE during the differentiation process, and on the 4th day, cells were exposed to 10 μM Dex for 24 h following a 1‐h starvation period. In OCR measurements, cells were treated with oligomycin (oligo, 1 μM) to measure the adenosine triphosphate (ATP) turnover. The maximum respiratory capacity was evaluated using carbonyl cyanide 4‐trifluoromethoxy phenylhydrazone (FCCP, 0.5 μM). Then, the mitochondrial oxygen consumption was blocked by a combination of antimycin A (1 μM) and rotenone (1 μM) (A+ *R*).

### 2.12 Protein–Protein Interaction Analysis

STRING (https://string‐db.org/), a comprehensive proteomic database, catalogs protein interactions and networks across species. It allows for searching for one or multiple proteins while specifying the desired species. The basic and advanced settings were as follows: type = Protein with Values/Ranks, organisms = *Mus musculus* , network type = full STRING network, meaning of network edges = evidence, active interaction sources = text mining, experiments, databases, co‐expression, neighborhood, gene fusion, co‐occurrence, minimum required interaction score = 0.400, maximum number of interactors to show = none/query protein only (1st shell), and network display options = none. The structure of the network consisted of lines and gene nodes. Lines represented “known interactions”, “predicted interactions”, and “others”, while gene nodes indicated associated proteins that contribute to a shared function. This does not necessarily imply physical binding between proteins, but rather that they are jointly involved in a specific and meaningful biological process, represented in unique colors.

GO and KEGG analyses of the 12 core gene targets were performed using an online SRPLOT platform (http://www.bioinformatics.com.cn/), which integrated R packages, including clusterProfiler and pathview.

### 2.13 Statistical Analyses

The trial results were reported as the mean ± standard error of the mean (SEM). Statistical analyses were conducted using one‐way analysis of variance (ANOVA) with Duncan's or Scheffe's multiple comparison test or Student's t‐test. Statistical significance was set at *p* < 0.05. All statistical analyses were performed using GraphPad Crystal 9.3.1 (La Jolla, California, USA) and Statistical Package for the Social Sciences (SPSS) (Version 16.0, SPSS software Inc., Chicago, IL, USA).

## 3 Results

### 3.1 PSE Enhanced the Dex and HFHS Diet‐Induced Muscle Fiber Size and Muscular Strength With Amelioration of Myosteatosis, but Not Altering Obesity

We first checked metabolic parameters, including BW, organ weight, and lipid and glucose profiles, in Dex and HFHS diet‐treated C57BL/6 mice treated with PSE at a dosage of 10 mg/kg along with the control. No significant differences were observed in the obesogenic parameters such as BW, body weight gain (BWG), organ weight (epididymal fat and hepatic tissue weight), blood chemistry (triglyceride, total cholesterol, glucose, insulin, and HOMA‐IR levels), and food intake of mice between Dex + HFHS vs. Dex + HFHS+PSE groups (Table 1).

**TABLE 1:** Characteristic of diet intake, metabolic parameters, and blood profiles*.

	Control	Dex + HFHS	Dex + HFHS+PSE	*p*
Diet intake				
Food intake (g/mouse/day)	4.03 ± 0.27^a^	1.29 ± 0.07^b^	1.15 ± 0.11^b^	< 0.0001
Drink intake (g/mouse/day)	—	7.97 ± 0.60	8.48 ± 0.74	n.s.
Total kcal intake (kcal/mouse/day)	15.65 ± 1.03^a^	12.67 ± 0.56^b^	12.29 ± 0.72^b^	< 0.0001
Phenotypes				
BW (g)	32.0 ± 1.13	32.06 ± 1.00	31.61 ± 1.67	n.s.
ΔBW gain (g)	9.10 ± 1.22^b^	13.56 ± 1.20^a^	12.56 ± 2.22^ab^	< 0.01
Liver (g)	1.05 ± 0.05^b^	1.55 ± 0.07^a^	1.57 ± 0.08^a^	< 0.0001
Liver/BW (%)	1.00 ± 0.05^b^	1.48 ± 0.06^a^	1.53 ± 0.02^a^	< 0.0001
Epididymal fat (g)	0.99 ± 0.12	1.15 ± 0.13	1.21 ± 0.22	n.s.
Epididymal fat/BW (%)	1.00 ± 0.09	1.17 ± 0.12	1.22 ± 0.16	n.s.
Blood Chemistry				
Triglyceride (mg/dL)	128.50 ± 10.56^b^	233.92 ± 20.39^a^	228.69 ± 14.17^a^	< 0.0001
Total Cholesterol (mg/dL)	223.83 ± 34.24^b^	401.94 ± 23.31^a^	379.43 ± 24.76^ab^	< 0.0001
Glucose (mg/dL)	73.2 ± 1.33^b^	100.3 ± 6.40^ab^	107.7 ± 8.12^a^	< 0.05
Insulin (ng/mL)	1.53 ± 0.64^b^	3.14 ± 0.36^ab^	3.65 ± 0.54^a^	< 0.05
HOMA‐IR	6.99 ± 3.03^b^	20.68 ± 3.50^a^	22.90 ± 2.77^a^	< 0.01

* Values are mean ± S.E.M. All groups *n* = 5–9/group. Values within a row with different superscript letters (a, b) are significantly different (*p* < 0.05), as determined by one‐way ANOVA followed by Duncan or Tukey’s multiple‐comparison test; values sharing a letter are not significantly different.

**FIGURE 1:**
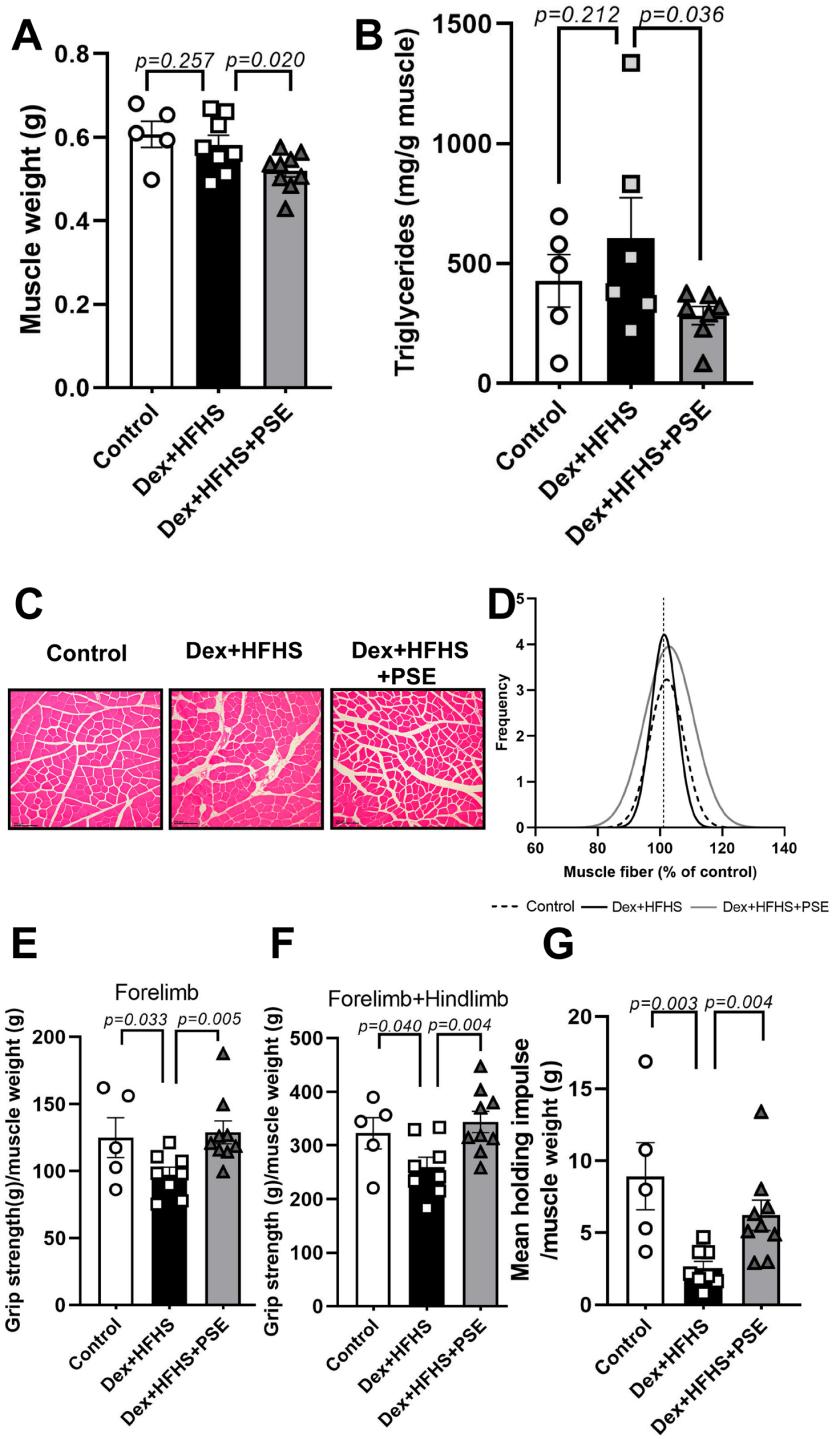
Peanut sprout extract (PSE) enhanced the Dex and HFHS diet‐induced muscle fiber size and muscular strength with amelioration of myosteatosis, but not altering obesity. Male C57BL/6 mice are fed control diet (Control, *n* = 5) or HFHS diet with daily administration of saline or PSE (10 mg/kg body weight [BW]) for 10 weeks. Dex was injected during the last six days to induce muscle atrophy (Dex + HFHS, *n* = 8; Dex + HFHS+PSE, *n* = 9 per group). (A) Skeletal muscle weight (g) combined weight of the quadriceps and gastrocnemius muscles from the experimental mice. (B) Gastrocnemius muscle triglycerides (TG) content (mg/g muscle). (C) Representative sections of the quadriceps muscle stained with hematoxylin and eosin (H&E) are examined at a 20× magnification. Scale bars measuring 100 μm are included to provide a visual reference for the size of the muscle fibers. (D) The frequency of muscle fiber, expressed as a percentage of the control group, is analyzed and reported. The grip strength and hanging test was performed at the 10th week. (E) Grip strength test of forelimbs (g)/muscle weight (g). (F) Grip strength test of forelimbs + hindlimbs (g)/muscle weight (g) of the mice. (G) Mean holding impulse/muscle weight (g). Data are expressed as the mean ± standard error of the mean (SEM); n.s., not significant; exact *p*‐value is shown in the graph by comparing Control vs. Dex + HFHS or Dex + HFHS vs. Dex + HFHS+PSE by Student's *t*‐test.

Next, we investigated whether PSE treatment alters myosteatosis and muscle function. Although one‐way ANOVA did not reveal a significant difference in skeletal muscle weight (combined weight of the quadriceps and gastrocnemius muscles) between Dex + HFHS vs. Dex + HFHS + PSE (Figure 1A), PSE treatment effectively mitigated the HFHS diet‐induced elevated TG levels in the gastrocnemius muscle (Figure 1B). There were no differences in adipocyte size and hepatic steatosis rate between groups (Figure S1). Histological examination of the quadriceps muscle revealed that the muscle fiber sizes in the PSE‐treated group were slightly increased compared to those in the Dex + HFHS‐treated groups (Figure 1C,D), although there were no significant differences in Fiber CSA among groups (Figure S1E). Grip strength and hanging tests were performed to assess the comprehensive muscle strength and endurance, enabling the assessment of overall muscle function and performance. PSE administration resulted in a significant enhancement in grip strength among mice whose muscular strength was downregulated by the combination of HFHS and Dex treatments in both forelimb (Figure 1E) and forelimb with hindlimb measurements (Figure 1F). Furthermore, the positive effect of PSE on muscle function was confirmed using the hanging test compared with the Dex + HFHS group (Figure 1G). Moreover, the unnormalized grip strength and hanging test data also showed significant improvement in the PSE‐treated group compared to the Dex + HFHS group (Figure S1F–H). These results indicated that PSE enhanced the Dex and HFHS diet‐induced muscle fiber size and muscular strength with amelioration of myosteatosis, but did not alter obesity.

### 3.2 PSE Attenuated Dex‐Induced Skeletal Muscle Atrophy and Inflammation in HFHS‐Fed Mice

**FIGURE 2:**
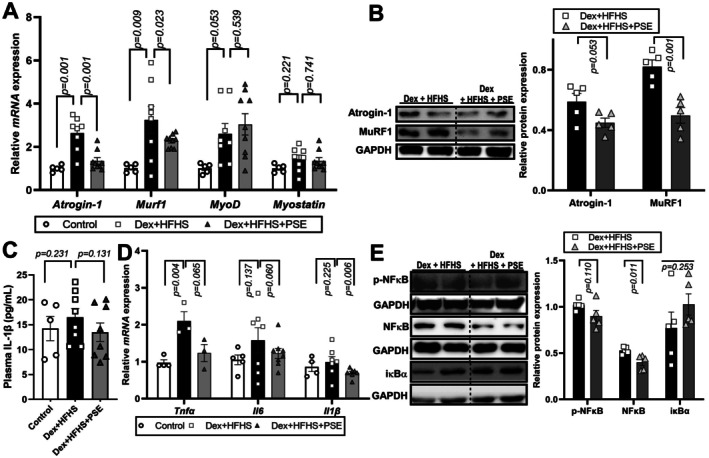
Peanut sprout extract (PSE) attenuated Dexamethasone (Dex)‐induced skeletal muscle atrophy in high‐fat and high‐sucrose (HFHS)‐fed mice. Male C57BL/6 mice are fed a control diet (Control, *n* = 5) or HFHS diet with daily administration of saline or PSE (10 mg/kg body weight [BW]) for 10 weeks. Dex was injected during the last six days to induce muscle atrophy (Dex + HFHS, *n* = 8; Dex + HFHS + PSE, *n* = 9 per group). (A) Relative mRNA expressions of *Atogin‐1, Murf1, Myod1* and *Myostatin* in gastrocnemius muscle using real‐time PCR. (B) Western blot analysis of muscle atrophy markers, Atrogin‐1 and MuRF1 in gastrocnemius muscle (left) and relative protein expression (right). Dashed lines in the western blot images indicate boundaries between different experimental groups (Dex + HFHS vs. Dex + HFHS + PSE), while all samples were processed on the same blot. (C) IL‐1β levels in blood plasma by ELISA. (D) Relative mRNA expressions of *Tnfα*, *Il6*, and *Il1β* in gastrocnemius muscle are quantified using real‐time PCR. (E) Phospho or total nuclear factor‐κB (NF‐κB) and IκBα protein expression in gastrocnemius muscle and glyceraldehyde 3‐phosphate dehydrogenase (GAPDH) served as a control. Dashed lines in the western blot images indicate boundaries between different experimental groups (Dex + HFHS vs. Dex + HFHS + PSE), while all samples were processed on the same blot. Data are expressed as the mean ± standard error of the mean (SEM); n.s., not significant; exact *p*‐value was shown in graph by comparing Control vs. Dex + HFHS or Dex + HFHS vs. Dex + HFHS + PSE by Student's *t*‐test.

To confirm the effect of PSE on the restoration of muscle strength in mice, we evaluated the MAFbx/Atrogin‐1 and MuRF1 expression levels, which are associated with muscle atrophy in skeletal muscles. As expected, *MAFbx/Atrogin‐1* and *MuRF1* gene expressions were significantly higher in the Dex + HFHS group compared to Control. Interestingly, PSE treatment exhibited a discernible decline of those genes compared to the Dex + HFHS group (Figure 2A, left). However, PSE treatment did not alter the *Myod1* expression, a significant marker for muscle differentiation, and *Myostatin*, a myokine involved in the regulation of muscle mass and function in the gastrocnemius muscle (Figure 2A, right). The protein expression of MuRF1 in the Dex + HFHS + PSE group was significantly reduced compared to that in the Dex + HFHS groups, but there were no significant differences in Atrogin‐1 expression (Figure 2B, original blots are shown in Figure S2).

To determine whether the previously reported anti‐inflammatory effect of PSE (Seo et al. 2021) extended in Dex‐ and HFHS‐treated mice, we assessed cytokine release. Plasma levels of IL‐1β had no differences among groups (Figure 2C). Next, we investigated the inflammatory status in the gastrocnemius muscle of Dex + HFHS fed mice. The Dex and HFHS‐mediated elevated *Tnfα*, *Il6*, and *Il1β* expressions were significantly reduced by PSE consumption (Figure 2D). Consistent with these results, the protein expression level of NF‐κB, a crucial regulator of cytokine production, was also reduced by PSE compared to the Dex + HFHS group (Figure 2E, original blots are shown in Figure S2). However, the expression of p‐NF‐κB and IκBα, a suppressor of the NF‐κB transcription factor, did not reach statistical significance by PSE (Figure 2E, original blots are shown in Figure S2). These results emphasize the potential anti‐inflammatory effects of PSE and its influence on cytokine production in skeletal muscle of Dex and HFHS‐induced muscle atrophy C57BL/6 mice.

### 3.3 PSE Facilitated Mitochondrial Activation in Skeletal Muscle of Dex‐ and HFHS‐Treated Mice

**FIGURE 3:**
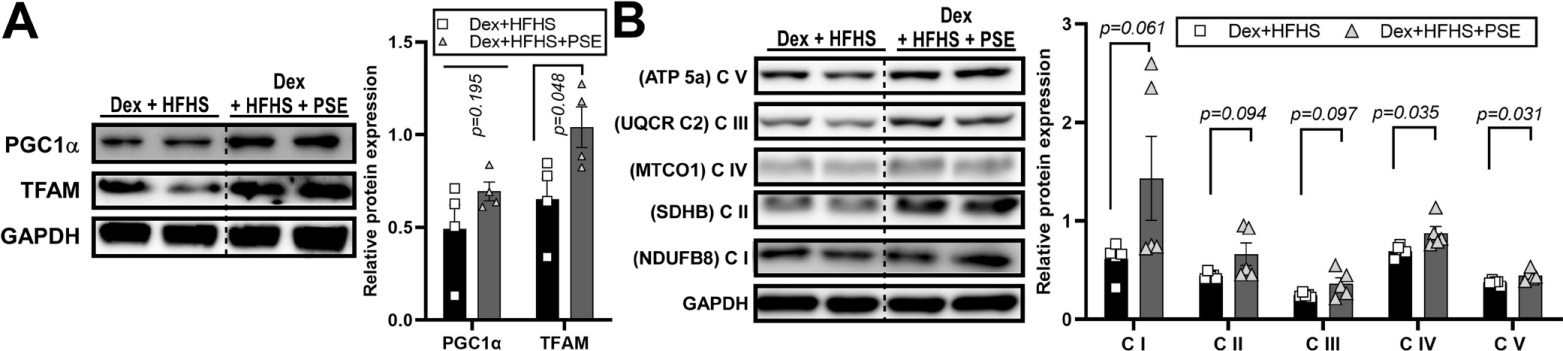
Peanut sprout extract (PSE) facilitated mitochondrial activation in the skeletal muscle of Dexamethasone (Dex) and high‐fat and high‐sucrose (HFHS)‐treated mice. Male C57BL/6 mice are fed a control diet (Control, *n* = 5) or HFHS diet with daily administration of saline or PSE (10 mg/kg body weight [BW]) for 10 weeks. Dex was injected during the last six days to induce muscle atrophy (Dex + HFHS, *n* = 8; Dex + HFHS + PSE, *n* = 9 per group). (A) Western blot analysis of peroxisome proliferator‐activated receptor‐γ coactivator 1‐α (PGC1α) and mitochondrial transcription factor A (TFAM) in gastrocnemius muscle and relative protein expression. GAPDH served as a control. (B) Western blot of oxidative phosphorylation (OXPHOS) protein expression in gastrocnemius muscle and relative protein expression. GAPDH served as a control. Dashed lines in the western blot images indicate boundaries between different experimental groups (Dex + HFHS vs. Dex + HFHS + PSE), while all samples were processed on the same blot. Data are expressed as the mean ± standard error of the mean (SEM); n.s., not significant; exact *p*‐values were shown in the graph by comparing Control vs. Dex + HFHS or Dex + HFHS vs. Dex + HFHS + PSE by Student's *t*‐test.

Consumption of an HFHS diet has been reported to induce mitochondrial dysfunction (Ferey et al. 2019; Sverdlov et al. 2015). In this study, we assessed the effect of PSE treatment on mitigating mitochondrial dysfunction in the skeletal muscle of HFHS‐fed mice by assessing the mitochondrial biogenesis markers and mitochondrial oxidative capacity. The PSE‐treated group exhibited a significant increase in the protein expression level of TFAM compared with the Dex + HFHS group. Additionally, a similar pattern was observed in the protein expression of PGC1α, although the difference was not statistically significant (Figure 3A, original blots are shown in Figure S2). Furthermore, western blot analysis of OXPHOS expression in the gastrocnemius muscles revealed an enhancement in PSE treatment compared to that in the Dex + HFHS groups. Specifically, complexes 4 (C4) and 5 (C5) exhibited significant expression compared to that in the Dex + HFHS group (Figure 3B, original blots are shown in Figure S2). These results show that PSE treatment enhanced mitochondrial function and biogenesis in skeletal muscles, emphasizing its potential therapeutic significance in mitochondria‐associated conditions.

### 3.4 Effects of PSE on Dex‐Induced Muscle Atrophy and Mitochondrial Function in C2C12 Cells

**FIGURE 4:**
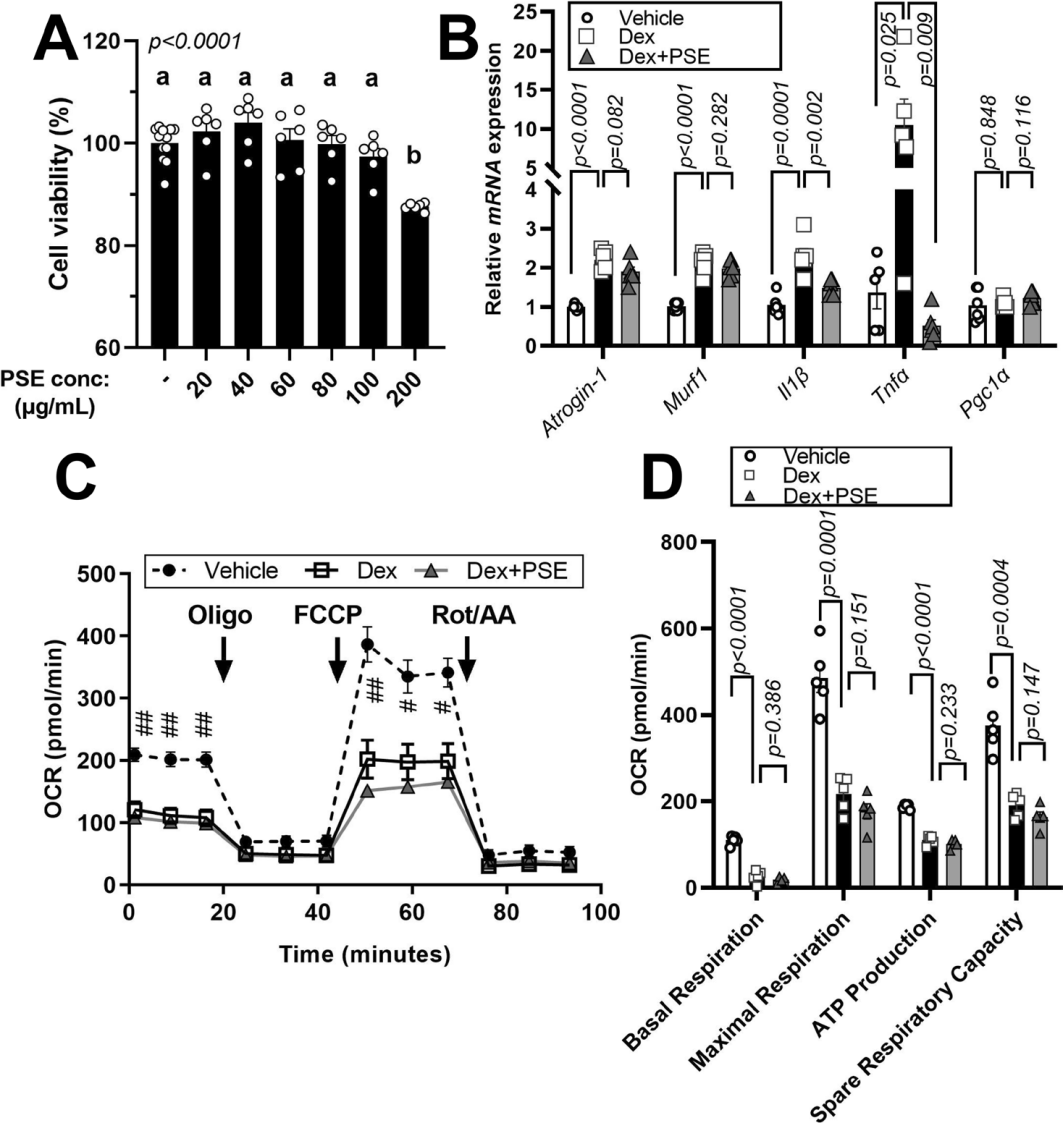
Effects of PSE on Dex‐induced muscle atrophy and mitochondrial function in C2C12 cells. The C2C12 cells were incubated with PSE extract at different doses (0–200 μg/mL). XTT reagent was added for 3 h into 96‐well plates to measure cell viability. (A) cell viability by XTT. (B) The C2C12 cells were incubated with 25 μg/mL of PSE for five days during differentiation. After starving for 1 h, they are treated with 10 μM Dex and harvested after 24 h. Atrophy and inflammatory gene expressions of *Atrogin‐1*, *Murf1, Il1β, Tnfα*, and *Pgc1α a*re quantified using real‐time PCR. (C) Oxygen consumption rate (OCR) in C2C12 cells treated with Veh (black circle, dot line), Dex (transparent square, black line) or Dex + PSE (Gray triangle, gray line) as determined by Seahorse extracellular analyzer. The C2C12 cells are treated with 25 μg/mL PSE for five days during differentiation. After starving for 1 h, they are treated with 10 μM Dex. Arrow indicates the addition of respiratory inhibitors of oligomycin (Oligo), Carbonyl cyanide 4‐trifluoromethoxyphenylhydrazone (FCCP) and combination of antimycin A and rotenone (Rot/AA). (D) OCR profiles. Data are expressed as the mean ± standard error of the mean (SEM); Bars with different letters are significantly different by one‐way ANOVA with Duncan or Tukey's comparison test. n.s., not significant; exact *p*‐value were shown in graph by comparing Control vs. Dex + HFHS or Dex + HFHS vs. Dex + HFHS+PSE by Student's *t*‐test.

To confirm the protective effects of PSE against muscle atrophy, in vitro experiments were conducted using C2C12 mouse myoblasts. PSE exhibited no cytotoxicity in C2C12 cells up to a concentration of 100 μg/mL and was used at a concentration of 25 μg/mL, consistent with a previous study (Figure 4A; Seo et al. 2019; Seo et al. 2021). PSE treatment significantly reduced the upregulation of Dex‐induced *Atrogin1* expression, with a slight reduction in *Murf1* expression (Figure 4B, left). Furthermore, PSE treatment significantly reduced the *Il‐1β* and *Tnfα* expression, inflammatory markers (Figure 4B, middle). Additionally, PSE treatment has a trend to increase in *Pgc1α* expression, a marker associated with mitochondrial activity, compared to the other experimental groups, but there were no significant differences (Figure 4B, right). We also tested a Dex and sodium palmitate–bovine serum albumin (BSA) complex (750 μM) in vitro to simulate the Dex + HFHS condition. Consistent with our gene expression data (Figure 4B), PSE treatment downregulated atrophic and inflammatory markers while upregulating *Pgc1α* (Figure S3). However, the Dex + PA condition did not result in an increase in *Atrogin1*, *Il‐1β*, and *Tnfα* gene expression. We used Dex treatment alone to assess the protective effects of PSE against muscle atrophy in vitro. The downregulated mitochondrial respiration rate (basal and maximal respiration, ATP production, and spare respiratory capacity) had no changes by PSE treatment (Figure 4C). These results indicate the protective effects of PSE against muscle atrophy and its potential to modulate the gene expression associated with muscle function and inflammation in vitro.

### 3.5 Network and Pathway Analyses Associate PSE Treatment With Suppressed Inflammatory Signaling and Enhanced Mitochondrial Respiration in Dex + HFHS Muscle

**FIGURE 5:**
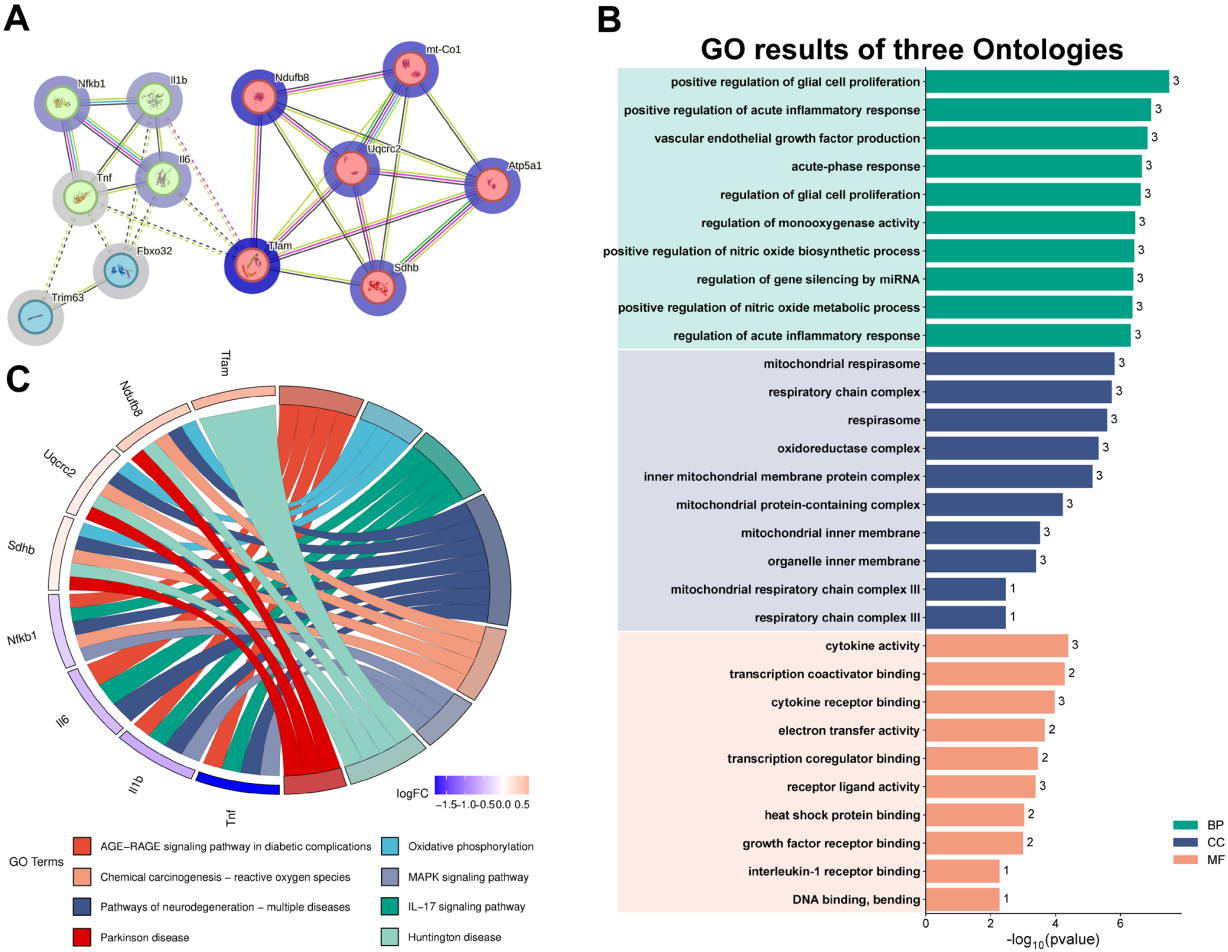
Results of GO and KEGG Enrichment Analysis. (A) The protein–protein interaction (PPI) network of the 12 intersecting targets analyzed by STRING. The network was connected by lines between genes, with each characteristic indicated by a unique color. (B) Bar plot chart of GO functional enrichment analysis for biological processes (BP), cellular components (CC), and molecular functions (MF). The x‐axis represents the negative log_10_ (pvalue), and the y‐axis represents the gene count for different GO terms. (C) KEGG enrichment chord plot. Genes are shown on the left, with red gene bands indicating up‐regulated expression and blue gene bands indicating down‐regulated expression. The different colored bands on the right represent different pathways. Lines connect genes to their corresponding pathways. The outer semicircle represents the log2 fold change (log2FC) value of differentially expressed genes (DEGs) (*p* value ≤ 0.05) in significant KEGG‐enriched pathways.

A protein–protein interaction (PPI) network was constructed using the 12 identified genes (Table S4). After removing unconnected nodes, the final network comprised 12 nodes and 29 edges, with a PPI enrichment *p*‐value of 3.82e‐05, indicating statistically significant and complex interconnections among the proteins (Figure 5A). To further investigate the functional roles of these genes, Gene Ontology (GO) enrichment analysis was performed, yielding 986 significant terms, including 915 biological processes (BPs), 26 cellular components (CCs), and 27 molecular functions (MFs). Among the top enriched BP terms were response to positive regulation of glial cell proliferation, positive regulation of acute inflammatory response, and vascular endothelial growth factor production. Enriched CC terms were predominantly associated with the mitochondrial respirasome, respiratory chain complex, and respirasome. Key MF terms include cytokine activity, transcription coactivator binding, cytokine receptor binding (Figure 5B; Tables S5–S7).

To further explore the significant differentially expressed genes (DEGs), the multiple KEGG pathways converge on mitochondrial dysfunction, inflammation, and energy imbalance, forming a pathogenic network underlying skeletal muscle atrophy, were chosen for further KEGG enrichment string analysis. As shown in the enriched chord diagram, the hub genes *Tnf*, *Il1b*, *Il6*, and *Nfkb1* were downregulated while *Sdhb*, *Uqcrc2*, *Ndufb8*, and *Tfam* were upregulated related to the AGE‐RAGE signaling pathway, oxidative phosphorylation, IL‐17 signaling pathway, pathways of neurodegeneration—multiple diseases, chemical carcinogenesis—reactive oxygen species, MAPK signaling pathway, huntington disease, and parkinson disease (Figure 5C; Tables S8 and S9).

## 4 Discussion

Consumption of a HFHS diet elevates the risk of obesity and metabolic syndrome, initiating inflammatory responses and reactive oxygen species (ROS) production, specifically causing mitochondrial metabolic abnormalities (Jorgensen et al. 2017; Mendez et al. 2014; Rasool et al. 2018). Skeletal muscle with mitochondrial dysfunction undergoes catabolism, upregulating the expression of muscle atrophy genes, thereby resulting in skeletal muscle atrophy (Chen et al. 2023; Romanello and Sandri 2021). In our previous studies, we demonstrated that PSE inhibits lipid accumulation by enhancing mitochondrial fatty acid oxidation in adipocytes and mitigates LPS‐induced inflammation and adipocyte browning through mitochondrial activation (Seo et al. 2019, 2021). However, the effects of PSE on Dex‐ and HFHS‐induced myosteatosis and atrophy remain unclear. Therefore, we evaluated the potential of PSE to mitigate muscle atrophy exacerbated through Dex treatment in HFHS‐fed male C57BL6 mice.

HFHS diets induce various metabolic complications and obesity (Rasool et al. 2018). In our previous study, we demonstrated that PSE partially mitigated obesity induced by a high‐fat diet (Seo et al. 2021). That study incorporated peanut sprout (PS) powder into the diet for *ad libitum* and provided relatively high daily intakes of crude extracts. Such crude‐extract approaches are common in functional foods studies, often employing doses in the range of 100–1000 mg/kg BW, but it might be accompanied by potential xenobiotic burden. In our study, we used a concentrated extract and administered it via oral gavage at a standardized dose of 10 mg/kg BW which is approximately 10–100 fold lower than typical crude‐extract regimen. This strategy was chosen to minimize potential off‐target effects. Based on our previous findings (Seo et al. 2021) and unpublished data, we propose that *p*‐coumaric acid (PCA), a key bioactive component of PSE, may contribute to its anti‐atrophic effects. In our previous study, PCA attenuated the mild muscle atrophy induced by HFHS feeding. However, HFHS‐driven atrophy develops gradually and remains relatively modest, limiting its value as a robust intervention model. Accordingly, in the current study we employed a Dex + HFHS model to better emulate compounded metabolic (HFHS) and catabolic (dexamethasone) stress. In a preliminary mouse study using the same Dex + HFHS‐induced muscle atrophy model, PCA supplementation (10 mg/kg BW) improved functional outcomes such as grip strength and hanging capacity, despite limited effects on muscle weight or TG content. These findings suggest that PCA may partially mediate the muscle‐protective actions of PSE. To further investigate the nutritional composition, we analyzed both PS powder and its ethanol extract (PSE) and presented the findings in Table S3. This analysis highlights differences in caloric content, macronutrients, and specific bioactives, confirming that the extraction process substantially alters the profile of functional components, particularly PCA. In the current study, we administered PSE via oral gavage at a standardized dose of 10 mg/kg BW to ensure consistent daily intake across animals. This dosage, smaller than typical in other studies, was selected to minimize potential side effects, such as vomiting. Nevertheless, it remains uncertain whether the 10 mg/kg BW dosage and oral gavage method are sufficient to effectively distribute the beneficial effects of PSE throughout the body. In our current study, no significant variations were observed in metabolic indicators, including BW, glucose, adipose accumulation, and plasma lipid profiles (Table 1). Despite the absence of observable alterations in the skeletal muscle weight among the groups (Figure 1A), PSE appeared to preserve the muscle atrophic features including muscle lipid accumulation, fiber size with altering atrophic markers (Figures 1 and 2). According to Abrigo et al., excessive weight gain, obesity, and sarcopenic obesity are associated with diminished physical abilities (Abrigo et al. 2016; Hsu et al. 2019). Although our animal model did not use aged model, we confirmed that PSE treatment preserved grip strength and hanging potential in mice that were compromised by HFHS and Dex (Figure 1E–G).

GCs and HFHS contribute to muscle atrophy (Rasool et al. 2018; Schakman et al. 2013). Our assessment confirmed a significant increase in muscle atrophy markers in mice following 10 weeks of HFHS consumption and Dex treatment. The PSE‐treated group exhibited a substantial reduction in Atrogin1 expression compared to that in the Dex group and a reduction compared to that in the HFHS group, although not statistically significant. Similarly, *Murf1* expression was reduced in the PSE‐treated group compared to the Dex group and modestly reduced compared to the HFHS group. Furthermore, MuRF1 protein expression in the PSE group was significantly lower than that in the Dex and HFHS groups (Figure 2A,B). Although not statistically significant, PSE induced a modest reduction in *Myostatin* expression, which is a significant regulator of muscle mass and a slight increase in *Myod1* expression, a marker of muscle differentiation, compared to the other groups (Figure 2A). Muscle function is a more crucial determinant than muscle mass and composition (McGregor et al. 2014). Actual muscle function exhibited a reverse trend compared to gene expression associated with muscle atrophy. Although our experimental model did not involve aged subjects, recent findings have reported that associated with a reduction in quadriceps muscle fiber size, particularly affecting type II fibers, which are more susceptible to atrophy (Nilwik et al. 2013). Gunder et al. reported that dexamethasone treatment reduced muscle strength, muscle size, and the proportion of type II muscle fibers, with obesity further exacerbating these effects (Gunder et al. 2020). Consistent with these findings, our study demonstrated a reduction in quadriceps muscle fiber size (Figure 1C,D) alongside altered expression of atrophic markers (Figure 2A,B) in Dex + HFHS‐fed mice. While we have provided both histological and molecular evidence supporting the use of the Dex + HFHS model to study myosteatosis in animals, a potential limitation of our study lies in the use of a discrete muscle type. Future studies are warranted examining the same muscle type to ensure comparability and further validate these findings. Thus, our results demonstrate PSE might serve as an effective treatment unprotecting or against Dex‐ and HFHS‐induced muscle atrophy by suppressing the expression of *Atrogin1* and *Murf1*. Animal numbers were determined a priori using the resource‐equation approach, which recommends maintaining the error degrees of freedom for ANOVA between 10 and 20; in our design, the error DF was 12, indicating an acceptable exploratory sample size within this framework. Nonetheless, relatively small group sizes (*n* = 5–9) increase the risk of Type II error, and therefore future studies incorporating larger cohorts will be necessary to more robustly detect modest effects and validate the present findings.

Mitochondrial dysfunction is a crucial factor in skeletal muscle atrophy (Carafoli et al. 1964; Chen et al. 2023; Matsumoto et al. 2022). TFAM, which shares structural similarities with damage‐associated molecular patterns, serves as an initiator of inflammation (Chaung et al. 2012; Little et al. 2014). In the present Dex + HFHS model, PSE increased TFAM and OXPHOS protein expression in skeletal muscle, suggesting preservation of mitochondrial biogenesis markers and respiratory chain components under combined metabolic and catabolic stress. OXPHOS C1 serves as the primary entry point for electrons into the respiratory chain and is the rate‐limiting step in overall respiration, playing a crucial role in energy metabolism (Sharma et al. 2009). Therefore, the observed mitochondrial activation effect of PSE against HFHS and Dex treatment may be attributed to an increase in C1. However, these protein‐level changes were not accompanied by demonstrable improvements in mitochondrial respiration in Dex‐treated C2C12 myotubes, and functional assays of mitochondrial OCR or ATP production were not performed in vivo. Therefore, our mitochondrial findings should be interpreted as evidence for partial maintenance of mitochondrial protein content rather than definitive proof of enhanced mitochondrial oxidative capacity, and future studies incorporating direct functional measurements in skeletal muscle will be essential to validate this putative mitochondrial benefit. Consumption of HFHS diet induces inflammation in the muscles and body (Collins et al. 2017), contributing to chronic inflammation and skeletal muscle atrophy (Haddad et al. 2005; Irazoki et al. 2022; Ji et al. 2022; Tuttle et al. 2020). The NF‐κB signaling pathway, activated by proinflammatory cytokines, plays a significant role in facilitating muscle breakdown (Guttridge 2004; Li et al. 2008). In our study, PSE treatment produced a modest, non‐significant reduction in plasma IL‐1β and decreased NF‐κB protein levels while increasing its inhibitor IκBα (Figure 2C,E). PSE also slightly reduced *Il1β* and *Il6* mRNA and significantly increased Tnfα expression in Dex + HFHS‐treated muscle, indicating a complex and only partially resolved inflammatory profile (Figure 2D). More comprehensive profiling of muscle cytokines will be needed to define PSE's full anti‐inflammatory effects. In C2C12 cells, PSE did not affect viability across the tested concentrations (Figure 4A). Co‐treatment with PSE during differentiation attenuated Dex‐induced atrophic gene expression, modestly increased *Pgc1α*, and significantly reduced *Tnfα* and *Il1β* mRNA, supporting a direct anti‐atrophic and anti‐inflammatory action in vitro (Figure 4B). To place these in vitro effects within the in vivo Dex + HFHS context, we conducted an integrated systems analysis of the differentially expressed genes (Figure 5). This analysis identified a compact 12‐gene module with significant network connectivity and a clear functional polarity‐anti‐inflammatory (TNF/IL‐1β/IL‐6/NF‐κB↓) and pro‐mitochondrial (TFAM/OXPHOS subunits↑)‐that mechanistically accords with the improved muscle function observed after PSE treatment in the combined Dex + HFHS atrophy model. In our previous study, we demonstrated an increase in mitochondrial activity, as evidenced by the induction of maximal respiration rate in adipocytes (Seo et al. 2019, 2021). However, in our current experiments, PSE did not induce mitochondrial activation. In our previous study, PSE (25–50 μg/mL treatment) significantly upregulated OCR in PA‐treated human hepatoma cells and differentiated mouse adipocytes (Seo et al. 2019). We presumably assume that PSE or PSE's responsible bioactive components such as PCA (Seo et al. 2021) or resveratrol (Seo et al. 2019) may interact with lipid metabolism. Therefore, future studies investigating the effects of Dex in combination with other fatty acids, such as oleic or palmitic acid, on muscle atrophy are warranted. These additional experiments will contribute to a comprehensive understanding of the efficacy of PSE in enhancing muscle atrophy under conditions that closely mimic the intricate metabolic environment associated with HFHS.

Recent studies have identified Dex as an effective agent for inducing muscle atrophy, with applying various doses (1 mg/kg BW to 25 mg/kg BW), delivery methods (e.g., intraperitoneal injection, drinking water), and treatment durations (5–18 days) (Britto et al. 2014; Geng et al. 2020; Hong et al. 2019; Kweon et al. 2019; Shen et al. 2019; Sun et al. 2014; Yeon et al. 2020). In our study, we aimed to develop an animal model of sarcopenic obesity by administering short‐term Dex treatment alongside a HFHS diet. Using a correlation model to calculate the Dex dose and treatment duration (𝑦 = −0.0949 𝑥 + 11.295), we derived an optimal acute yet subtle Dex concentration of 10 mg/kg BW, administered over 10 days. HFHS diet was administered over 8 weeks, a timeframe that is widely accepted in recent studies to induce metabolic complications (Parks et al. 2013). Given that diet was an additional factor in our study, we adjusted Dex duration to 6 days. While we established a rationale for the Dex dosage and HFHS feeding duration, a notable limitation of our study is the absence of a Dex‐only treatment group with or without PSE. Dexamethasone can regulate muscle protein turnover by influencing both synthesis and degradation; however, our study focused only on protein degradation pathways. Future work employing a full 3 × 3 factorial design (Dex, HFHS, and PSE) and concurrently assessing protein synthesis pathways (e.g., mTOR/Akt/S6) and degradation pathways would be essential to clarify the relative contribution of glucocorticoid versus dietary stress and to refine the mechanistic interpretation of PSE's protective actions.

While the observed effects of PSE on myosteatosis and muscle atrophy are promising, it remains unclear whether these effects are primarily driven by HFHS‐induced obesity or Dex‐induced muscle atrophy. Preliminary data from HFHS‐fed mice, with or without PSE treatment, showed that PSE upregulated muscle fiber CSA and grip strength (forelimb) while reducing *Atrogin‐1* and *MuRF1* gene expression in muscle with lowered plasma IL‐1β levels. However, unlike in Dex‐treated mice, PSE did not affect HFHS‐mediated lowered grip strength (forelimb + hindlimb) and hanging capacity with PGC1α and TFAM protein expression (data not shown), suggesting that TFAM induction by PSE may be specific to Dex‐induced atrophy. Additionally, preliminary findings indicate that PSE enhanced mitochondrial complexes IV (CIV) in HFHS‐fed mice, with a significant increase in ATP synthase (CV) and marginal increases in complexes I (CI)/II and III (CIII). These results suggest that grip strength and TFAM induction may be unique to the Dex‐induced model (data not shown). However, as these experiments were not conducted simultaneously, future studies with a refined experimental design are needed to distinguish the effects of Dex from HFHS‐induced atrophy and mitochondrial dysfunction. To address this, we are pursuing three complementary lines of investigation: (i) dose–response studies in a Dex‐induced atrophy model with and without concurrent HFHS feeding; (ii) mechanistic testing in TLR4‐deficient mice, administering PSE or PCA to determine whether anti‐atrophic effects are mediated, at least in part, through TLR4‐linked inflammatory signaling; and (iii) efficacy studies in aged mice to evaluate PSE's ability to protect against age‐associated skeletal muscle atrophy and strength loss. These experiments are designed to disentangle Dex‐ versus diet‐specific effects, define mechanisms, and clarify the translational scope of PSE.

Considering PCA as a key bioactive component in PSE, we estimated the theoretical systemic exposure based on published bioavailability data. Pei et al. reported that serum concentrations of PCA peaked at 165 μmol/L within 10 min after oral administration of 100 μmol/kg in rats (Pei et al. 2016). Similarly, Garrait et al. demonstrated that a single oral dose of PCA (0.1 mol/L/kg) was absorbed across multiple intestinal segments, with tissue levels reaching steady state within 1 h and serum concentrations approximating 5 μmol/L at 180 min (Garrait et al. 2006). Based on these studies and our previous data (Table S3, Seo et al. 2021), we estimated that oral administration of 10 mg/kg BW PSE contains ~5 μg of PCA per kg BW, potentially resulting in serum concentrations of ~5 μM and tissue levels of ~0.15 μM after a single dose. With repeated daily dosing over 10 weeks, cumulative exposure may reach ~40 μM in serum and ~12 μM in tissues, assuming linear accumulation. Therefore, we guess the 25 μM concentration of PSE used in our in vitro experiments falls within the estimated physiological range corresponding to the in vivo dosage, supporting its translational relevance. We intentionally selected a relatively low oral gavage dose (10 mg/kg BW) to identify the minimum effective dose with consistent bioavailability, as our previous study using 4% PS in the diet (Seo et al. 2021) showed only modest effects, possibly due to variability in intake. A key limitation of this study is the absence of single‐component, head‐to‐head experiments within the current dataset; consequently, we cannot ascribe the observed effects to any specific constituent of PSE. To address this, we are conducting an in vivo bioavailability study to quantify plasma and tissue concentrations of putative bioactives following PSE administration. In parallel, future work will refine the dose‐duration relationship of PSE to optimize physiological efficacy while maintaining practicality for dietary applications. For component‐level attribution and standardization, we will undertake comprehensive LC–MS/MS profiling of PSE lots to define batch composition and quantify candidate actives (e.g., PCA), implement fractionation with bioassay‐guided testing to localize activity to discrete chemical fractions, and perform reconstitution studies to evaluate synergy among constituents.

## 5 Conclusion

This study explored the potential of PSE in mitigating HFHS‐ and Dex‐induced muscle atrophy in male C57BL/6 mice. Although PSE did not have a significant effect on body weight or glucose parameters, it effectively preserved muscle fiber size and reduced TG content in the skeletal muscle. PSE exhibited significant protective effects against Dex‐ and HFHS‐induced muscle atrophy markers, modulated inflammatory responses, and enhanced mitochondrial function. In vitro experiments with C2C12 cells further support the ability of PSE to alleviate atrophy and inflammation. Furthermore, we provided a network‐level, mechanism‐anchored readout in which a compact 12‐gene module shows significant PPI enrichment and bidirectional polarity (TNF/IL‐1β/IL‐6/NF‐κB↓; TFAM/OXPHOS subunits↑) across oxidative phosphorylation and inflammatory pathways, aligning with the functional improvements. These findings indicate the diverse potential of PSE in protecting against skeletal muscle atrophy and preserving muscle strength. This emphasizes the need for further assessment, specifically in aged mouse models, to elucidate its broader implications in the complex metabolic scenarios associated with HFHS.

## Author Contributions

S.‐M.J. contributed to data curation, formal analysis, investigation, and writing‐original draft, T.M.T.T. contributed to data curation, formal analysis, investigation, and writing‐review and editing, H.‐J.J. contributed to methodology and writing‐review and editing, J.H.L. contributed to methodology, resources, and writing‐review and editing, and I.K. contributed to conceptualization, project administration, supervision, and writing‐review and editing.

## Funding

This research was supported by Korean government (MSIT) RS‐2023‐00208776, RS‐2023‐00221563 through National Research Foundation of Korea (NRF) and Regional Innovation System & Education (RISE) program through the Jeju RISE center, funded by the Ministry of Education (MOE) and the Jeju Special Self‐Governing Province, Republic of Korea (2025‐RISE‐17‐001).

## Conflicts of Interest

The authors declare no conflicts of interest.

## Supporting information


**Appendix S1:** fsn371469‐sup‐0001‐AppendixS1.docx.

## Data Availability

The data that support the findings of this study are available from the corresponding author upon reasonable request.
