# Supplementary material for: Mitigating Dexamethasone‐Induced Muscle Wasting and Mitochondrial Impairment in Mice on a High‐Fat and High‐Sucrose Diet With Peanut Sprout Extract
Source: Food Sci Nutr. 2026 Jan 19;14(1):e71469. doi: 10.1002/fsn3.71469 (PMC12816884; doi:10.1002/fsn3.71469)
Supplement: Supplementary file 1 — Appendix S1: fsn371469‐sup‐0001‐AppendixS1.docx. [file FSN3-14-e71469-s001.docx]

**Supplementary information**

Table S1. Dietary composition

| **Ingredients** | **LF** | **HF** |
| --- | --- | --- |
|  | g/kg | g/kg |
| Casein | 200 | 200 |
| l-Cysteine | 3 | 3 |
| Sucrose | 0 | 69 |
| Corn starch | 600 | 0 |
| Maltodextrin 10 | 50 | 125 |
| Lard | 10 | 245 |
| Cholesterol | 0 | 2 |
| Soybean oil | 39 | 39 |
| Cellulose | 50 | 50 |
| Mineral mix | 35 | 35 |
| Calcium phosphate | 4 | 4 |
| Vitamin mix | 10 | 10 |
| Choline bitartrate | 2 | 2 |
| Total | 1003 | 784 |
|  | (3.9 kcal/g) | (4.2 kcal/g) |
|  |  |  |
|  | kcal (%) | kcal (%) |
| Carbohydrate | 67.8 | 19.8 |
| Protein | 20.9 | 19.3 |
| Fat | 11.3 | 60.8 |
|  | 100 | 100 |

The AIN-93G diet was modified

Table S2. Primer sequences for real-time PCR

| **Gene** | **Forward** | **Reverse** |
| --- | --- | --- |
| *mIl6* | CTGCAAGAGACTTCCATCCAGTT | AGGGAAGGCCGTGGTTGT |
| *mTnfα* | GGCTGCCCCGACTACGT | ACTTTCTCCTGGTATGAGATAGCAAAT |
| *m36b4* | GGATCTGCTGCATCTGCTTG | GGCGACCTGGAAGTCCAACT |
| *mHprt* | TTGCTCGAGATGTCATGAAGGA | AGCAGGTCAGCAAAGAACTTATAGC |
| *mMurf1* | AGTGTCCATGTCTGGAGGTCGTTT | ACTGGAGCACTCCTGCTTGTAGAT |
| *mAtrogin-1* | GAGGCAGATTCGCAAGCGTTTGAT | TCCAGGAGAGAATGTGGCAGTGTT |
| *mMyostatin* | TCACGCTACCACGGAAACAA | AGGAGTCTTGACGGGTCTGA |
| *mPgc1α* | CCCTGCCATTGTTAAGACC | TGCTGCTGTTCCTGTTTTC |
| *mMyod1* | CCACTCCGGGACATAGACTTG | AAAAGCGCAGGTCTGGTGAG |
| *mIl1β* | AAATACCTGTGGCCTTGGGC | CTTGGGATCCACACTCTCCAG |

Table S3. Nutrition composition

| **Nutrients** | PS powder  (Amount per 100g) | PSE  (Amount per 100g) |
| --- | --- | --- |
| **Calories (kcal/100g)** | **407.0** | **10.98** |
| **Sodium (mg/100g)** | **60.9** | **2.89** |
| **Total Carbohydrate (g/100g)** | **38.9** | **1.64** |
| **Total Sugars (g/100g)** | **3.2** | **0.73** |
| **Total Fat (g/100g)** | **25.1** | **0.14** |
| **Trans Fat (g/100g)** | **-** | **-** |
| **Saturated Fat (g/100g)** | **4.7** | **0.02** |
| **Cholesterol (mg/100g)** | **3.6** | **-** |
| **Protein (g/100g)** | **24.1** | **0.79** |
| **Cellulose (g/100g)** | **35.7** |  |
| **PCA (mg/100g)** | **25.4** | **42.9** |

**Table S4.** **Gene ID**

| **Gene ID** | **Entry** | **String** | **DexHFHS/PSE** | **Log2FC** |
| --- | --- | --- | --- | --- |
| Atrogin1 (Fbxo32) | [Q9CPU7](https://www.uniprot.org/uniprotkb/Q9CPU7/entry) | [ENSMUSP00000022986](https://string-db.org/network/10090.ENSMUSP00000022986) | 0.333904172 | -1.58249 |
| Murf1 (TRIM63) | [Q969Q1](https://www.uniprot.org/uniprotkb/Q969Q1/entry) | [ENSP00000363390](https://string-db.org/network/9606.ENSP00000363390) | 0.304547901 | -1.71526 |
| Tnfα | [P06804](https://www.uniprot.org/uniprotkb/P06804/entry) | ENSMUSP00000025263 | 0.287000567 | -1.80087 |
| Il6 | P08505 | [ENSMUSP00000026845](https://string-db.org/network/10090.ENSMUSP00000026845) | 0.692146199 | -0.53085 |
| Il1β | [P10749](https://www.uniprot.org/uniprotkb/P10749/entry) | [ENSMUSP00000028881](https://string-db.org/network/10090.ENSMUSP00000028881) | 0.635471309 | -0.6541 |
| NFkB (Nfkb1) | [P25799](https://www.uniprot.org/uniprotkb/P25799/entry) | [ENSMUSP00000029812](https://string-db.org/network/10090.ENSMUSP00000029812) | 0.770816593 | -0.37554 |
| TFAM | [P40630](https://www.uniprot.org/uniprotkb/P40630/entry) | [ENSMUSP00000090086](https://string-db.org/network/10090.ENSMUSP00000090086) | 1.59733431 | 0.675666 |
| NDUFB8 | [Q9D6J5](https://www.uniprot.org/uniprotkb/Q9D6J5/entry) | [ENSMUSP00000026222](https://string-db.org/network/10090.ENSMUSP00000026222) | 1.3600701 | 0.443681 |
| SDHB | [Q9CQA3](https://funcoup.org/uniprot/Q9CQA3) | [ENSMUSP00000010007](https://string-db.org/network/10090.ENSMUSP00000010007) | 1.109023571 | 0.14929 |
| UQCRC2 | [Q9DB77](https://www.ebi.ac.uk/intact/search?query=id:Q9DB77*#interactor) | [ENSMUSP00000033176](https://string-db.org/network/10090.ENSMUSP00000033176) | 1.140572333 | 0.189758 |
| MTCO1 | [P00397](https://mint.bio.uniroma2.it/cgi-bin/protein.py?id=P00397) | [ENSMUSP00000080993](https://string-db.org/network/10090.ENSMUSP00000080993) | 1.18575058 | 0.245801 |
| ATP5a | [Q03265](https://mint.bio.uniroma2.it/cgi-bin/protein.py?id=Q03265) | [ENSMUSP00000026495](https://string-db.org/network/10090.ENSMUSP00000026495) | 1.137779639 | 0.186221 |

**Table S5. Biological processes (BPs)**

| **ID** | **Description** | **Gene**  **Ratio** | **Bg**  **Ratio** | **pvalue** | **p.adjust** | **qvalue** | **Gene**  **ID** | **Count** | **Enrichment**  **Score** | **Fold**  **Enrichment** |
| --- | --- | --- | --- | --- | --- | --- | --- | --- | --- | --- |
| GO:0060252 | positive regulation of glial cell proliferation | 3/9 | 22/29008 | 3.17075E-08 | 3.48466E-05 | 6.14125E-06 | Tnf/Il6/Il1b | 3 | 7.4988 | 439.5152 |
| GO:0002675 | positive regulation of acute inflammatory response | 3/9 | 33/29008 | 1.12144E-07 | 4.65396E-05 | 8.20198E-06 | Tnf/Il6/Il1b | 3 | 6.9502 | 293.0101 |
| GO:0010573 | vascular endothelial growth factor production | 3/9 | 36/29008 | 1.46688E-07 | 4.65396E-05 | 8.20198E-06 | Tnf/Il6/Il1b | 3 | 6.8336 | 268.5926 |
| GO:0006953 | acute-phase response | 3/9 | 41/29008 | 2.18836E-07 | 4.65396E-05 | 8.20198E-06 | Tnf/Il6/Il1b | 3 | 6.6599 | 235.8374 |
| GO:0060251 | regulation of glial cell proliferation | 3/9 | 42/29008 | 2.35632E-07 | 4.65396E-05 | 8.20198E-06 | Tnf/Il6/Il1b | 3 | 6.6278 | 230.2222 |
| GO:0032768 | regulation of monooxygenase activity | 3/9 | 48/29008 | 3.54678E-07 | 4.65396E-05 | 8.20198E-06 | Tnf/Il1b/Nfkb1 | 3 | 6.4502 | 201.4444 |
| GO:0045429 | positive regulation of nitric oxide biosynthetic process | 3/9 | 49/29008 | 3.77751E-07 | 4.65396E-05 | 8.20198E-06 | Tnf/Il6/Il1b | 3 | 6.4228 | 197.3333 |
| GO:0060964 | regulation of gene silencing by miRNA | 3/9 | 50/29008 | 4.018E-07 | 4.65396E-05 | 8.20198E-06 | Tnf/Il6/Nfkb1 | 3 | 6.3960 | 193.3867 |
| GO:1904407 | positive regulation of nitric oxide metabolic process | 3/9 | 51/29008 | 4.26846E-07 | 4.65396E-05 | 8.20198E-06 | Tnf/Il6/Il1b | 3 | 6.3697 | 189.5948 |
| GO:0002673 | regulation of acute inflammatory response | 3/9 | 53/29008 | 4.80009E-07 | 4.65396E-05 | 8.20198E-06 | Tnf/Il6/Il1b | 3 | 6.3188 | 182.4403 |
| GO:0060147 | regulation of posttranscriptional gene silencing | 3/9 | 53/29008 | 4.80009E-07 | 4.65396E-05 | 8.20198E-06 | Tnf/Il6/Nfkb1 | 3 | 6.3188 | 182.4403 |
| GO:0060966 | regulation of gene silencing by RNA | 3/9 | 54/29008 | 5.08166E-07 | 4.65396E-05 | 8.20198E-06 | Tnf/Il6/Nfkb1 | 3 | 6.2940 | 179.0617 |
| GO:0014009 | glial cell proliferation | 3/9 | 58/29008 | 6.31763E-07 | 4.95934E-05 | 8.74017E-06 | Tnf/Il6/Il1b | 3 | 6.1994 | 166.7126 |
| GO:0032757 | positive regulation of interleukin-8 production | 3/9 | 58/29008 | 6.31763E-07 | 4.95934E-05 | 8.74017E-06 | Tnf/Il6/Il1b | 3 | 6.1994 | 166.7126 |
| GO:0045428 | regulation of nitric oxide biosynthetic process | 3/9 | 72/29008 | 1.21845E-06 | 7.90886E-05 | 1.39383E-05 | Tnf/Il6/Il1b | 3 | 5.9142 | 134.2963 |
| GO:0060968 | regulation of gene silencing | 3/9 | 74/29008 | 1.32395E-06 | 7.90886E-05 | 1.39383E-05 | Tnf/Il6/Nfkb1 | 3 | 5.8781 | 130.6667 |
| GO:0080164 | regulation of nitric oxide metabolic process | 3/9 | 75/29008 | 1.3789E-06 | 7.90886E-05 | 1.39383E-05 | Tnf/Il6/Il1b | 3 | 5.8605 | 128.9244 |
| GO:0071222 | cellular response to lipopolysaccharide | 4/9 | 303/29008 | 1.41109E-06 | 7.90886E-05 | 1.39383E-05 | Tnf/Il6/Il1b/Nfkb1 | 4 | 5.8504 | 42.5493 |
| GO:0032677 | regulation of interleukin-8 production | 3/9 | 76/29008 | 1.43534E-06 | 7.90886E-05 | 1.39383E-05 | Tnf/Il6/Il1b | 3 | 5.8430 | 127.2281 |
| GO:0032637 | interleukin-8 production | 3/9 | 77/29008 | 1.4933E-06 | 7.90886E-05 | 1.39383E-05 | Tnf/Il6/Il1b | 3 | 5.8259 | 125.5758 |
| GO:0009615 | response to virus | 4/9 | 312/29008 | 1.5853E-06 | 7.90886E-05 | 1.39383E-05 | Tnf/Il6/Il1b/Nfkb1 | 4 | 5.7999 | 41.3219 |
| GO:0071219 | cellular response to molecule of bacterial origin | 4/9 | 313/29008 | 1.60559E-06 | 7.90886E-05 | 1.39383E-05 | Tnf/Il6/Il1b/Nfkb1 | 4 | 5.7944 | 41.1899 |
| GO:0071347 | cellular response to interleukin-1 | 3/9 | 80/29008 | 1.67645E-06 | 7.90886E-05 | 1.39383E-05 | Il6/Il1b/Nfkb1 | 3 | 5.7756 | 120.8667 |
| GO:0014015 | positive regulation of gliogenesis | 3/9 | 82/29008 | 1.80648E-06 | 7.90886E-05 | 1.39383E-05 | Tnf/Il6/Il1b | 3 | 5.7432 | 117.9187 |
| GO:0032722 | positive regulation of chemokine production | 3/9 | 83/29008 | 1.87393E-06 | 7.90886E-05 | 1.39383E-05 | Tnf/Il6/Il1b | 3 | 5.7272 | 116.4980 |
| GO:0051384 | response to glucocorticoid | 3/9 | 83/29008 | 1.87393E-06 | 7.90886E-05 | 1.39383E-05 | Fbxo32/Tnf/Il6 | 3 | 5.7272 | 116.4980 |
| GO:0006809 | nitric oxide biosynthetic process | 3/9 | 84/29008 | 1.94303E-06 | 7.90886E-05 | 1.39383E-05 | Tnf/Il6/Il1b | 3 | 5.7115 | 115.1111 |
| GO:0071216 | cellular response to biotic stimulus | 4/9 | 337/29008 | 2.15341E-06 | 8.45214E-05 | 1.48958E-05 | Tnf/Il6/Il1b/Nfkb1 | 4 | 5.6669 | 38.2565 |
| GO:0050727 | regulation of inflammatory response | 4/9 | 342/29008 | 2.28309E-06 | 8.47696E-05 | 1.49395E-05 | Tnf/Il6/Il1b/Nfkb1 | 4 | 5.6415 | 37.6972 |
| GO:0046209 | nitric oxide metabolic process | 3/9 | 89/29008 | 2.314E-06 | 8.47696E-05 | 1.49395E-05 | Tnf/Il6/Il1b | 3 | 5.6356 | 108.6442 |
| GO:0031960 | response to corticosteroid | 3/9 | 90/29008 | 2.39342E-06 | 8.48507E-05 | 1.49538E-05 | Fbxo32/Tnf/Il6 | 3 | 5.6210 | 107.4370 |
| GO:2001057 | reactive nitrogen species metabolic process | 3/9 | 91/29008 | 2.47463E-06 | 8.49881E-05 | 1.4978E-05 | Tnf/Il6/Il1b | 3 | 5.6065 | 106.2564 |
| GO:0051341 | regulation of oxidoreductase activity | 3/9 | 95/29008 | 2.81774E-06 | 9.38393E-05 | 1.65379E-05 | Tnf/Il1b/Nfkb1 | 3 | 5.5501 | 101.7825 |
| GO:0002718 | regulation of cytokine production involved in immune response | 3/9 | 97/29008 | 3.00053E-06 | 9.42167E-05 | 1.66044E-05 | Tnf/Il6/Il1b | 3 | 5.5228 | 99.6838 |
| GO:0070555 | response to interleukin-1 | 3/9 | 97/29008 | 3.00053E-06 | 9.42167E-05 | 1.66044E-05 | Il6/Il1b/Nfkb1 | 3 | 5.5228 | 99.6838 |
| GO:0062012 | regulation of small molecule metabolic process | 4/9 | 369/29008 | 3.08643E-06 | 9.42217E-05 | 1.66053E-05 | Tnf/Il6/Il1b/Nfkb1 | 4 | 5.5105 | 34.9389 |
| GO:0032611 | interleukin-1 beta production | 3/9 | 99/29008 | 3.19102E-06 | 9.4782E-05 | 1.67041E-05 | Tnf/Il6/Il1b | 3 | 5.4961 | 97.6700 |
| GO:0032496 | response to lipopolysaccharide | 4/9 | 388/29008 | 3.76597E-06 | 0.000108916 | 1.9195E-05 | Tnf/Il6/Il1b/Nfkb1 | 4 | 5.4241 | 33.2279 |
| GO:0045833 | negative regulation of lipid metabolic process | 3/9 | 106/29008 | 3.9206E-06 | 0.000110481 | 1.94707E-05 | Tnf/Il1b/Nfkb1 | 3 | 5.4066 | 91.2201 |
| GO:0032755 | positive regulation of interleukin-6 production | 3/9 | 109/29008 | 4.26438E-06 | 0.000114695 | 2.02135E-05 | Tnf/Il6/Il1b | 3 | 5.3701 | 88.7095 |
| GO:0002526 | acute inflammatory response | 3/9 | 110/29008 | 4.38326E-06 | 0.000114695 | 2.02135E-05 | Tnf/Il6/Il1b | 3 | 5.3582 | 87.9030 |
| GO:0032642 | regulation of chemokine production | 3/9 | 110/29008 | 4.38326E-06 | 0.000114695 | 2.02135E-05 | Tnf/Il6/Il1b | 3 | 5.3582 | 87.9030 |
| GO:0002367 | cytokine production involved in immune response | 3/9 | 111/29008 | 4.50432E-06 | 0.000114771 | 2.02268E-05 | Tnf/Il6/Il1b | 3 | 5.3464 | 87.1111 |
| GO:0002237 | response to molecule of bacterial origin | 4/9 | 409/29008 | 4.64003E-06 | 0.000114771 | 2.02268E-05 | Tnf/Il6/Il1b/Nfkb1 | 4 | 5.3335 | 31.5219 |
| GO:0031620 | regulation of fever generation | 2/9 | 11/29008 | 4.69944E-06 | 0.000114771 | 2.02268E-05 | Tnf/Il1b | 2 | 5.3280 | 586.0202 |
| GO:0032602 | chemokine production | 3/9 | 114/29008 | 4.88074E-06 | 0.000116607 | 2.05505E-05 | Tnf/Il6/Il1b | 3 | 5.3115 | 84.8187 |
| GO:0042362 | fat-soluble vitamin biosynthetic process | 2/9 | 12/29008 | 5.63841E-06 | 0.00013039 | 2.29796E-05 | Tnf/Nfkb1 | 2 | 5.2488 | 537.1852 |
| GO:0032612 | interleukin-1 production | 3/9 | 120/29008 | 5.69494E-06 | 0.00013039 | 2.29796E-05 | Tnf/Il6/Il1b | 3 | 5.2445 | 80.5778 |
| GO:0031652 | positive regulation of heat generation | 2/9 | 13/29008 | 6.66251E-06 | 0.000149431 | 2.63352E-05 | Tnf/Il1b | 2 | 5.1764 | 495.8632 |
| GO:0014013 | regulation of gliogenesis | 3/9 | 128/29008 | 6.9139E-06 | 0.000151968 | 2.67823E-05 | Tnf/Il6/Il1b | 3 | 5.1603 | 75.5417 |
| GO:0001660 | fever generation | 2/9 | 14/29008 | 7.77168E-06 | 0.000161152 | 2.8401E-05 | Tnf/Il1b | 2 | 5.1095 | 460.4444 |
| GO:0030213 | hyaluronan biosynthetic process | 2/9 | 14/29008 | 7.77168E-06 | 0.000161152 | 2.8401E-05 | Il1b/Nfkb1 | 2 | 5.1095 | 460.4444 |
| GO:0034116 | positive regulation of heterotypic cell-cell adhesion | 2/9 | 14/29008 | 7.77168E-06 | 0.000161152 | 2.8401E-05 | Tnf/Il1b | 2 | 5.1095 | 460.4444 |
| GO:0050729 | positive regulation of inflammatory response | 3/9 | 134/29008 | 7.93349E-06 | 0.000161461 | 2.84554E-05 | Tnf/Il6/Il1b | 3 | 5.1005 | 72.1592 |
| GO:0042359 | vitamin D metabolic process | 2/9 | 15/29008 | 8.96588E-06 | 0.000175955 | 3.10098E-05 | Tnf/Nfkb1 | 2 | 5.0474 | 429.7481 |
| GO:1903799 | negative regulation of production of miRNAs involved in gene silencing by miRNA | 2/9 | 15/29008 | 8.96588E-06 | 0.000175955 | 3.10098E-05 | Tnf/Il6 | 2 | 5.0474 | 429.7481 |
| GO:0031650 | regulation of heat generation | 2/9 | 16/29008 | 1.02451E-05 | 0.000194126 | 3.42122E-05 | Tnf/Il1b | 2 | 4.9895 | 402.8889 |
| GO:0051044 | positive regulation of membrane protein ectodomain proteolysis | 2/9 | 16/29008 | 1.02451E-05 | 0.000194126 | 3.42122E-05 | Tnf/Il1b | 2 | 4.9895 | 402.8889 |
| GO:0035195 | gene silencing by miRNA | 3/9 | 149/29008 | 1.09065E-05 | 0.000203157 | 3.58037E-05 | Tnf/Il6/Nfkb1 | 3 | 4.9623 | 64.8949 |
| GO:0002824 | positive regulation of adaptive immune response based on somatic recombination of immune receptors built from immunoglobulin superfamily domains | 3/9 | 152/29008 | 1.15779E-05 | 0.000212068 | 3.73743E-05 | Tnf/Il6/Il1b | 3 | 4.9364 | 63.6140 |
| GO:0035194 | post-transcriptional gene silencing by RNA | 3/9 | 155/29008 | 1.22761E-05 | 0.000220624 | 3.8882E-05 | Tnf/Il6/Nfkb1 | 3 | 4.9109 | 62.3828 |
| GO:0002708 | positive regulation of lymphocyte mediated immunity | 3/9 | 158/29008 | 1.30015E-05 | 0.000220624 | 3.8882E-05 | Tnf/Il6/Il1b | 3 | 4.8860 | 61.1983 |
| GO:0030656 | regulation of vitamin metabolic process | 2/9 | 18/29008 | 1.30583E-05 | 0.000220624 | 3.8882E-05 | Tnf/Nfkb1 | 2 | 4.8841 | 358.1235 |
| GO:0030730 | sequestering of triglyceride | 2/9 | 18/29008 | 1.30583E-05 | 0.000220624 | 3.8882E-05 | Tnf/Il1b | 2 | 4.8841 | 358.1235 |
| GO:0060965 | negative regulation of gene silencing by miRNA | 2/9 | 18/29008 | 1.30583E-05 | 0.000220624 | 3.8882E-05 | Tnf/Il6 | 2 | 4.8841 | 358.1235 |
| GO:0002821 | positive regulation of adaptive immune response | 3/9 | 159/29008 | 1.32495E-05 | 0.000220624 | 3.8882E-05 | Tnf/Il6/Il1b | 3 | 4.8778 | 60.8134 |
| GO:0016441 | posttranscriptional gene silencing | 3/9 | 161/29008 | 1.37548E-05 | 0.000225619 | 3.97624E-05 | Tnf/Il6/Nfkb1 | 3 | 4.8615 | 60.0580 |
| GO:1901550 | regulation of endothelial cell development | 2/9 | 19/29008 | 1.45922E-05 | 0.000232417 | 4.09605E-05 | Tnf/Il1b | 2 | 4.8359 | 339.2749 |
| GO:1903140 | regulation of establishment of endothelial barrier | 2/9 | 19/29008 | 1.45922E-05 | 0.000232417 | 4.09605E-05 | Tnf/Il1b | 2 | 4.8359 | 339.2749 |
| GO:0032675 | regulation of interleukin-6 production | 3/9 | 166/29008 | 1.50733E-05 | 0.000236651 | 4.17065E-05 | Tnf/Il6/Il1b | 3 | 4.8218 | 58.2490 |
| GO:0007254 | JNK cascade | 3/9 | 170/29008 | 1.61863E-05 | 0.000240754 | 4.24297E-05 | Tnf/Il1b/Nfkb1 | 3 | 4.7909 | 56.8784 |
| GO:0009110 | vitamin biosynthetic process | 2/9 | 20/29008 | 1.62109E-05 | 0.000240754 | 4.24297E-05 | Tnf/Nfkb1 | 2 | 4.7902 | 322.3111 |
| GO:0060149 | negative regulation of posttranscriptional gene silencing | 2/9 | 20/29008 | 1.62109E-05 | 0.000240754 | 4.24297E-05 | Tnf/Il6 | 2 | 4.7902 | 322.3111 |
| GO:0060967 | negative regulation of gene silencing by RNA | 2/9 | 20/29008 | 1.62109E-05 | 0.000240754 | 4.24297E-05 | Tnf/Il6 | 2 | 4.7902 | 322.3111 |
| GO:0032635 | interleukin-6 production | 3/9 | 174/29008 | 1.73522E-05 | 0.000250922 | 4.42217E-05 | Tnf/Il6/Il1b | 3 | 4.7606 | 55.5709 |
| GO:0071356 | cellular response to tumor necrosis factor | 3/9 | 174/29008 | 1.73522E-05 | 0.000250922 | 4.42217E-05 | Tnf/Il6/Nfkb1 | 3 | 4.7606 | 55.5709 |
| GO:0002922 | positive regulation of humoral immune response | 2/9 | 21/29008 | 1.79144E-05 | 0.000255688 | 4.50616E-05 | Tnf/Il1b | 2 | 4.7468 | 306.9630 |
| GO:0002700 | regulation of production of molecular mediator of immune response | 3/9 | 177/29008 | 1.82622E-05 | 0.000257309 | 4.53474E-05 | Tnf/Il6/Il1b | 3 | 4.7384 | 54.6290 |
| GO:0002705 | positive regulation of leukocyte mediated immunity | 3/9 | 183/29008 | 2.01755E-05 | 0.000280669 | 4.94642E-05 | Tnf/Il6/Il1b | 3 | 4.6952 | 52.8379 |
| GO:0031047 | gene silencing by RNA | 3/9 | 184/29008 | 2.05067E-05 | 0.000281711 | 4.96478E-05 | Tnf/Il6/Nfkb1 | 3 | 4.6881 | 52.5507 |
| GO:0050796 | regulation of insulin secretion | 3/9 | 186/29008 | 2.11798E-05 | 0.000285683 | 5.03479E-05 | Tnf/Il6/Il1b | 3 | 4.6741 | 51.9857 |
| GO:0031649 | heat generation | 2/9 | 23/29008 | 2.15757E-05 | 0.000285683 | 5.03479E-05 | Tnf/Il1b | 2 | 4.6660 | 280.2705 |
| GO:0051043 | regulation of membrane protein ectodomain proteolysis | 2/9 | 23/29008 | 2.15757E-05 | 0.000285683 | 5.03479E-05 | Tnf/Il1b | 2 | 4.6660 | 280.2705 |
| GO:0035743 | CD4-positive, alpha-beta T cell cytokine production | 2/9 | 24/29008 | 2.35333E-05 | 0.000304272 | 5.3624E-05 | Il6/Il1b | 2 | 4.6283 | 268.5926 |
| GO:0070498 | interleukin-1-mediated signaling pathway | 2/9 | 24/29008 | 2.35333E-05 | 0.000304272 | 5.3624E-05 | Il6/Il1b | 2 | 4.6283 | 268.5926 |
| GO:0034612 | response to tumor necrosis factor | 3/9 | 196/29008 | 2.47649E-05 | 0.000308875 | 5.44351E-05 | Tnf/Il6/Nfkb1 | 3 | 4.6062 | 49.3333 |
| GO:0002726 | positive regulation of T cell cytokine production | 2/9 | 25/29008 | 2.55756E-05 | 0.000308875 | 5.44351E-05 | Il6/Il1b | 2 | 4.5922 | 257.8489 |
| GO:0034114 | regulation of heterotypic cell-cell adhesion | 2/9 | 25/29008 | 2.55756E-05 | 0.000308875 | 5.44351E-05 | Tnf/Il1b | 2 | 4.5922 | 257.8489 |
| GO:0060969 | negative regulation of gene silencing | 2/9 | 25/29008 | 2.55756E-05 | 0.000308875 | 5.44351E-05 | Tnf/Il6 | 2 | 4.5922 | 257.8489 |
| GO:1903798 | regulation of production of miRNAs involved in gene silencing by miRNA | 2/9 | 25/29008 | 2.55756E-05 | 0.000308875 | 5.44351E-05 | Tnf/Il6 | 2 | 4.5922 | 257.8489 |
| GO:1904996 | positive regulation of leukocyte adhesion to vascular endothelial cell | 2/9 | 25/29008 | 2.55756E-05 | 0.000308875 | 5.44351E-05 | Tnf/Il6 | 2 | 4.5922 | 257.8489 |
| GO:0046890 | regulation of lipid biosynthetic process | 3/9 | 202/29008 | 2.70967E-05 | 0.000323687 | 5.70456E-05 | Tnf/Il1b/Nfkb1 | 3 | 4.5671 | 47.8680 |
| GO:0010575 | positive regulation of vascular endothelial growth factor production | 2/9 | 26/29008 | 2.77024E-05 | 0.000323883 | 5.70801E-05 | Il6/Il1b | 2 | 4.5575 | 247.9316 |
| GO:0070920 | regulation of production of small RNA involved in gene silencing by RNA | 2/9 | 26/29008 | 2.77024E-05 | 0.000323883 | 5.70801E-05 | Tnf/Il6 | 2 | 4.5575 | 247.9316 |
| GO:0043491 | protein kinase B signaling | 3/9 | 205/29008 | 2.83149E-05 | 0.000327559 | 5.7728E-05 | Tnf/Il6/Il1b | 3 | 4.5480 | 47.1675 |
| GO:0010829 | negative regulation of glucose transmembrane transport | 2/9 | 27/29008 | 2.99138E-05 | 0.000342451 | 6.03525E-05 | Tnf/Il1b | 2 | 4.5241 | 238.7490 |
| GO:0050995 | negative regulation of lipid catabolic process | 2/9 | 28/29008 | 3.22097E-05 | 0.00035756 | 6.30153E-05 | Tnf/Il1b | 2 | 4.4920 | 230.2222 |
| GO:0060445 | branching involved in salivary gland morphogenesis | 2/9 | 28/29008 | 3.22097E-05 | 0.00035756 | 6.30153E-05 | Tnf/Il6 | 2 | 4.4920 | 230.2222 |
| GO:1900017 | positive regulation of cytokine production involved in inflammatory response | 2/9 | 28/29008 | 3.22097E-05 | 0.00035756 | 6.30153E-05 | Tnf/Il6 | 2 | 4.4920 | 230.2222 |
| GO:0002822 | regulation of adaptive immune response based on somatic recombination of immune receptors built from immunoglobulin superfamily domains | 3/9 | 220/29008 | 3.49498E-05 | 0.000384098 | 6.76922E-05 | Tnf/Il6/Il1b | 3 | 4.4566 | 43.9515 |
| GO:0002706 | regulation of lymphocyte mediated immunity | 3/9 | 225/29008 | 3.73696E-05 | 0.00039861 | 7.02497E-05 | Tnf/Il6/Il1b | 3 | 4.4275 | 42.9748 |
| GO:0048545 | response to steroid hormone | 3/9 | 226/29008 | 3.78664E-05 | 0.00039861 | 7.02497E-05 | Fbxo32/Tnf/Il6 | 3 | 4.4217 | 42.7847 |
| GO:0030212 | hyaluronan metabolic process | 2/9 | 31/29008 | 3.96039E-05 | 0.00039861 | 7.02497E-05 | Il1b/Nfkb1 | 2 | 4.4023 | 207.9427 |
| GO:0032770 | positive regulation of monooxygenase activity | 2/9 | 31/29008 | 3.96039E-05 | 0.00039861 | 7.02497E-05 | Tnf/Il1b | 2 | 4.4023 | 207.9427 |
| GO:0035094 | response to nicotine | 2/9 | 31/29008 | 3.96039E-05 | 0.00039861 | 7.02497E-05 | Tnf/Nfkb1 | 2 | 4.4023 | 207.9427 |
| GO:0071354 | cellular response to interleukin-6 | 2/9 | 31/29008 | 3.96039E-05 | 0.00039861 | 7.02497E-05 | Il6/Nfkb1 | 2 | 4.4023 | 207.9427 |
| GO:1901889 | negative regulation of cell junction assembly | 2/9 | 31/29008 | 3.96039E-05 | 0.00039861 | 7.02497E-05 | Tnf/Il1b | 2 | 4.4023 | 207.9427 |
| GO:2000637 | positive regulation of gene silencing by miRNA | 2/9 | 31/29008 | 3.96039E-05 | 0.00039861 | 7.02497E-05 | Il6/Nfkb1 | 2 | 4.4023 | 207.9427 |
| GO:0030073 | insulin secretion | 3/9 | 230/29008 | 3.98973E-05 | 0.00039861 | 7.02497E-05 | Tnf/Il6/Il1b | 3 | 4.3991 | 42.0406 |
| GO:0090276 | regulation of peptide hormone secretion | 3/9 | 230/29008 | 3.98973E-05 | 0.00039861 | 7.02497E-05 | Tnf/Il6/Il1b | 3 | 4.3991 | 42.0406 |
| GO:0010574 | regulation of vascular endothelial growth factor production | 2/9 | 32/29008 | 4.22374E-05 | 0.000411308 | 7.24875E-05 | Il6/Il1b | 2 | 4.3743 | 201.4444 |
| GO:0060148 | positive regulation of posttranscriptional gene silencing | 2/9 | 32/29008 | 4.22374E-05 | 0.000411308 | 7.24875E-05 | Il6/Nfkb1 | 2 | 4.3743 | 201.4444 |
| GO:0002791 | regulation of peptide secretion | 3/9 | 235/29008 | 4.2535E-05 | 0.000411308 | 7.24875E-05 | Tnf/Il6/Il1b | 3 | 4.3713 | 41.1461 |
| GO:0002819 | regulation of adaptive immune response | 3/9 | 236/29008 | 4.30759E-05 | 0.000411308 | 7.24875E-05 | Tnf/Il6/Il1b | 3 | 4.3658 | 40.9718 |
| GO:0090087 | regulation of peptide transport | 3/9 | 237/29008 | 4.36214E-05 | 0.000411308 | 7.24875E-05 | Tnf/Il6/Il1b | 3 | 4.3603 | 40.7989 |
| GO:0051403 | stress-activated MAPK cascade | 3/9 | 238/29008 | 4.41714E-05 | 0.000411308 | 7.24875E-05 | Tnf/Il1b/Nfkb1 | 3 | 4.3549 | 40.6275 |
| GO:0070741 | response to interleukin-6 | 2/9 | 33/29008 | 4.49551E-05 | 0.000411308 | 7.24875E-05 | Il6/Nfkb1 | 2 | 4.3472 | 195.3401 |
| GO:1901099 | negative regulation of signal transduction in absence of ligand | 2/9 | 33/29008 | 4.49551E-05 | 0.000411308 | 7.24875E-05 | Tnf/Il1b | 2 | 4.3472 | 195.3401 |
| GO:1904994 | regulation of leukocyte adhesion to vascular endothelial cell | 2/9 | 33/29008 | 4.49551E-05 | 0.000411308 | 7.24875E-05 | Tnf/Il6 | 2 | 4.3472 | 195.3401 |
| GO:2001240 | negative regulation of extrinsic apoptotic signaling pathway in absence of ligand | 2/9 | 33/29008 | 4.49551E-05 | 0.000411308 | 7.24875E-05 | Tnf/Il1b | 2 | 4.3472 | 195.3401 |
| GO:1903039 | positive regulation of leukocyte cell-cell adhesion | 3/9 | 240/29008 | 4.5285E-05 | 0.000411308 | 7.24875E-05 | Tnf/Il6/Il1b | 3 | 4.3440 | 40.2889 |
| GO:0016458 | gene silencing | 3/9 | 243/29008 | 4.69898E-05 | 0.000423294 | 7.45999E-05 | Tnf/Il6/Nfkb1 | 3 | 4.3280 | 39.7915 |
| GO:0001763 | morphogenesis of a branching structure | 3/9 | 245/29008 | 4.81495E-05 | 0.000430214 | 7.58196E-05 | Tnf/Il6/Il1b | 3 | 4.3174 | 39.4667 |
| GO:0031098 | stress-activated protein kinase signaling cascade | 3/9 | 248/29008 | 4.99242E-05 | 0.000442473 | 7.798E-05 | Tnf/Il1b/Nfkb1 | 3 | 4.3017 | 38.9892 |
| GO:0006775 | fat-soluble vitamin metabolic process | 2/9 | 36/29008 | 5.36138E-05 | 0.000471372 | 8.30731E-05 | Tnf/Nfkb1 | 2 | 4.2707 | 179.0617 |
| GO:0002724 | regulation of T cell cytokine production | 2/9 | 37/29008 | 5.66683E-05 | 0.000490382 | 8.64233E-05 | Il6/Il1b | 2 | 4.2467 | 174.2222 |
| GO:0007435 | salivary gland morphogenesis | 2/9 | 37/29008 | 5.66683E-05 | 0.000490382 | 8.64233E-05 | Tnf/Il6 | 2 | 4.2467 | 174.2222 |
| GO:0051091 | positive regulation of DNA-binding transcription factor activity | 3/9 | 267/29008 | 6.21699E-05 | 0.000533787 | 9.40728E-05 | Tnf/Il6/Il1b | 3 | 4.2064 | 36.2147 |
| GO:0031349 | positive regulation of defense response | 3/9 | 273/29008 | 6.64104E-05 | 0.000565775 | 9.97104E-05 | Tnf/Il6/Il1b | 3 | 4.1778 | 35.4188 |
| GO:0007431 | salivary gland development | 2/9 | 41/29008 | 6.97269E-05 | 0.00058946 | 0.000103885 | Tnf/Il6 | 2 | 4.1566 | 157.2249 |
| GO:0030072 | peptide hormone secretion | 3/9 | 285/29008 | 7.54522E-05 | 0.000632993 | 0.000111557 | Tnf/Il6/Il1b | 3 | 4.1223 | 33.9275 |
| GO:0045601 | regulation of endothelial cell differentiation | 2/9 | 43/29008 | 7.67599E-05 | 0.000639084 | 0.00011263 | Tnf/Il1b | 2 | 4.1149 | 149.9121 |
| GO:0022409 | positive regulation of cell-cell adhesion | 3/9 | 288/29008 | 7.78323E-05 | 0.000643141 | 0.000113345 | Tnf/Il6/Il1b | 3 | 4.1088 | 33.5741 |
| GO:0006024 | glycosaminoglycan biosynthetic process | 2/9 | 44/29008 | 8.04022E-05 | 0.000659418 | 0.000116214 | Il1b/Nfkb1 | 2 | 4.0947 | 146.5051 |
| GO:0001818 | negative regulation of cytokine production | 3/9 | 292/29008 | 8.10816E-05 | 0.000660064 | 0.000116328 | Tnf/Il6/Nfkb1 | 3 | 4.0911 | 33.1142 |
| GO:0002790 | peptide secretion | 3/9 | 293/29008 | 8.19076E-05 | 0.000661886 | 0.000116649 | Tnf/Il6/Il1b | 3 | 4.0867 | 33.0011 |
| GO:0006509 | membrane protein ectodomain proteolysis | 2/9 | 45/29008 | 8.41283E-05 | 0.000671251 | 0.000118299 | Tnf/Il1b | 2 | 4.0751 | 143.2494 |
| GO:0002699 | positive regulation of immune effector process | 3/9 | 296/29008 | 8.44187E-05 | 0.000671251 | 0.000118299 | Tnf/Il6/Il1b | 3 | 4.0736 | 32.6667 |
| GO:0002703 | regulation of leukocyte mediated immunity | 3/9 | 297/29008 | 8.52668E-05 | 0.000671251 | 0.000118299 | Tnf/Il6/Il1b | 3 | 4.0692 | 32.5567 |
| GO:0050708 | regulation of protein secretion | 3/9 | 298/29008 | 8.61205E-05 | 0.000671251 | 0.000118299 | Tnf/Il6/Il1b | 3 | 4.0649 | 32.4474 |
| GO:0050769 | positive regulation of neurogenesis | 3/9 | 298/29008 | 8.61205E-05 | 0.000671251 | 0.000118299 | Tnf/Il6/Il1b | 3 | 4.0649 | 32.4474 |
| GO:0002369 | T cell cytokine production | 2/9 | 46/29008 | 8.79381E-05 | 0.000680592 | 0.000119945 | Il6/Il1b | 2 | 4.0558 | 140.1353 |
| GO:0046883 | regulation of hormone secretion | 3/9 | 302/29008 | 8.95913E-05 | 0.000688538 | 0.000121346 | Tnf/Il6/Il1b | 3 | 4.0477 | 32.0177 |
| GO:2001239 | regulation of extrinsic apoptotic signaling pathway in absence of ligand | 2/9 | 47/29008 | 9.18317E-05 | 0.000700855 | 0.000123516 | Tnf/Il1b | 2 | 4.0370 | 137.1537 |
| GO:0002920 | regulation of humoral immune response | 2/9 | 48/29008 | 9.5809E-05 | 0.000721192 | 0.000127101 | Tnf/Il1b | 2 | 4.0186 | 134.2963 |
| GO:0010718 | positive regulation of epithelial to mesenchymal transition | 2/9 | 48/29008 | 9.5809E-05 | 0.000721192 | 0.000127101 | Il6/Il1b | 2 | 4.0186 | 134.2963 |
| GO:0035196 | production of miRNAs involved in gene silencing by miRNA | 2/9 | 49/29008 | 9.98699E-05 | 0.000746646 | 0.000131587 | Tnf/Il6 | 2 | 4.0006 | 131.5556 |
| GO:0015833 | peptide transport | 3/9 | 314/29008 | 0.000100551 | 0.00074666 | 0.000131589 | Tnf/Il6/Il1b | 3 | 3.9976 | 30.7941 |
| GO:0031050 | dsRNA processing | 2/9 | 51/29008 | 0.000108242 | 0.000787804 | 0.00013884 | Tnf/Il6 | 2 | 3.9656 | 126.3965 |
| GO:0061756 | leukocyte adhesion to vascular endothelial cell | 2/9 | 51/29008 | 0.000108242 | 0.000787804 | 0.00013884 | Tnf/Il6 | 2 | 3.9656 | 126.3965 |
| GO:0070918 | production of small RNA involved in gene silencing by RNA | 2/9 | 51/29008 | 0.000108242 | 0.000787804 | 0.00013884 | Tnf/Il6 | 2 | 3.9656 | 126.3965 |
| GO:0006023 | aminoglycan biosynthetic process | 2/9 | 52/29008 | 0.000112554 | 0.000808476 | 0.000142483 | Il1b/Nfkb1 | 2 | 3.9486 | 123.9658 |
| GO:0061028 | establishment of endothelial barrier | 2/9 | 52/29008 | 0.000112554 | 0.000808476 | 0.000142483 | Tnf/Il1b | 2 | 3.9486 | 123.9658 |
| GO:1903037 | regulation of leukocyte cell-cell adhesion | 3/9 | 333/29008 | 0.000119641 | 0.000853804 | 0.000150472 | Tnf/Il6/Il1b | 3 | 3.9221 | 29.0370 |
| GO:0035272 | exocrine system development | 2/9 | 54/29008 | 0.000121428 | 0.000860961 | 0.000151733 | Tnf/Il6 | 2 | 3.9157 | 119.3745 |
| GO:0042886 | amide transport | 3/9 | 343/29008 | 0.000130577 | 0.000908655 | 0.000160139 | Tnf/Il6/Il1b | 3 | 3.8841 | 28.1905 |
| GO:0034113 | heterotypic cell-cell adhesion | 2/9 | 56/29008 | 0.000130635 | 0.000908655 | 0.000160139 | Tnf/Il1b | 2 | 3.8839 | 115.1111 |
| GO:0043507 | positive regulation of JUN kinase activity | 2/9 | 56/29008 | 0.000130635 | 0.000908655 | 0.000160139 | Tnf/Il1b | 2 | 3.8839 | 115.1111 |
| GO:0042063 | gliogenesis | 3/9 | 344/29008 | 0.000131706 | 0.000910343 | 0.000160436 | Tnf/Il6/Il1b | 3 | 3.8804 | 28.1085 |
| GO:0033619 | membrane protein proteolysis | 2/9 | 58/29008 | 0.000140175 | 0.000956848 | 0.000168632 | Tnf/Il1b | 2 | 3.8533 | 111.1418 |
| GO:0051353 | positive regulation of oxidoreductase activity | 2/9 | 58/29008 | 0.000140175 | 0.000956848 | 0.000168632 | Tnf/Il1b | 2 | 3.8533 | 111.1418 |
| GO:0032731 | positive regulation of interleukin-1 beta production | 2/9 | 59/29008 | 0.00014507 | 0.000978111 | 0.000172379 | Tnf/Il6 | 2 | 3.8384 | 109.2580 |
| GO:1900015 | regulation of cytokine production involved in inflammatory response | 2/9 | 59/29008 | 0.00014507 | 0.000978111 | 0.000172379 | Tnf/Il6 | 2 | 3.8384 | 109.2580 |
| GO:0051962 | positive regulation of nervous system development | 3/9 | 359/29008 | 0.000149401 | 0.001001171 | 0.000176443 | Tnf/Il6/Il1b | 3 | 3.8256 | 26.9341 |
| GO:0031663 | lipopolysaccharide-mediated signaling pathway | 2/9 | 61/29008 | 0.00015511 | 0.001033126 | 0.000182075 | Tnf/Il1b | 2 | 3.8094 | 105.6758 |
| GO:0046879 | hormone secretion | 3/9 | 367/29008 | 0.000159443 | 0.001054609 | 0.000185861 | Tnf/Il6/Il1b | 3 | 3.7974 | 26.3470 |
| GO:0002534 | cytokine production involved in inflammatory response | 2/9 | 62/29008 | 0.000160254 | 0.001054609 | 0.000185861 | Tnf/Il6 | 2 | 3.7952 | 103.9713 |
| GO:0007159 | leukocyte cell-cell adhesion | 3/9 | 373/29008 | 0.000167257 | 0.001094142 | 0.000192828 | Tnf/Il6/Il1b | 3 | 3.7766 | 25.9231 |
| GO:0009914 | hormone transport | 3/9 | 375/29008 | 0.000169917 | 0.00110496 | 0.000194735 | Tnf/Il6/Il1b | 3 | 3.7698 | 25.7849 |
| GO:0010720 | positive regulation of cell development | 3/9 | 378/29008 | 0.000173957 | 0.001122409 | 0.00019781 | Tnf/Il6/Il1b | 3 | 3.7596 | 25.5802 |
| GO:0019216 | regulation of lipid metabolic process | 3/9 | 379/29008 | 0.000175317 | 0.001122409 | 0.00019781 | Tnf/Il1b/Nfkb1 | 3 | 3.7562 | 25.5128 |
| GO:0002720 | positive regulation of cytokine production involved in immune response | 2/9 | 65/29008 | 0.000176186 | 0.001122409 | 0.00019781 | Il6/Il1b | 2 | 3.7540 | 99.1726 |
| GO:0002440 | production of molecular mediator of immune response | 3/9 | 380/29008 | 0.000176685 | 0.001122409 | 0.00019781 | Tnf/Il6/Il1b | 3 | 3.7528 | 25.4456 |
| GO:0060688 | regulation of morphogenesis of a branching structure | 2/9 | 66/29008 | 0.000181663 | 0.00114437 | 0.00020168 | Tnf/Il1b | 2 | 3.7407 | 97.6700 |
| GO:1901214 | regulation of neuron death | 3/9 | 384/29008 | 0.000182225 | 0.00114437 | 0.00020168 | Tnf/Il6/Il1b | 3 | 3.7394 | 25.1806 |
| GO:0050994 | regulation of lipid catabolic process | 2/9 | 68/29008 | 0.000192865 | 0.001197505 | 0.000211044 | Tnf/Il1b | 2 | 3.7147 | 94.7974 |
| GO:1901224 | positive regulation of NIK/NF-kappaB signaling | 2/9 | 68/29008 | 0.000192865 | 0.001197505 | 0.000211044 | Tnf/Il1b | 2 | 3.7147 | 94.7974 |
| GO:0009306 | protein secretion | 3/9 | 393/29008 | 0.000195101 | 0.001204581 | 0.000212292 | Tnf/Il6/Il1b | 3 | 3.7097 | 24.6039 |
| GO:0035592 | establishment of protein localization to extracellular region | 3/9 | 394/29008 | 0.000196567 | 0.001206854 | 0.000212692 | Tnf/Il6/Il1b | 3 | 3.7065 | 24.5415 |
| GO:1902895 | positive regulation of pri-miRNA transcription by RNA polymerase II | 2/9 | 69/29008 | 0.00019859 | 0.001212502 | 0.000213687 | Tnf/Nfkb1 | 2 | 3.7020 | 93.4235 |
| GO:0019221 | cytokine-mediated signaling pathway | 3/9 | 397/29008 | 0.000201009 | 0.001220488 | 0.000215095 | Tnf/Il6/Il1b | 3 | 3.6968 | 24.3560 |
| GO:0071692 | protein localization to extracellular region | 3/9 | 401/29008 | 0.000207032 | 0.001250155 | 0.000220323 | Tnf/Il6/Il1b | 3 | 3.6840 | 24.1131 |
| GO:0032732 | positive regulation of interleukin-1 production | 2/9 | 71/29008 | 0.000210289 | 0.001256017 | 0.000221356 | Tnf/Il6 | 2 | 3.6772 | 90.7919 |
| GO:1903510 | mucopolysaccharide metabolic process | 2/9 | 71/29008 | 0.000210289 | 0.001256017 | 0.000221356 | Il1b/Nfkb1 | 2 | 3.6772 | 90.7919 |
| GO:0043506 | regulation of JUN kinase activity | 2/9 | 72/29008 | 0.000216262 | 0.001277807 | 0.000225196 | Tnf/Il1b | 2 | 3.6650 | 89.5309 |
| GO:0098586 | cellular response to virus | 2/9 | 72/29008 | 0.000216262 | 0.001277807 | 0.000225196 | Il6/Nfkb1 | 2 | 3.6650 | 89.5309 |
| GO:0001885 | endothelial cell development | 2/9 | 74/29008 | 0.000228457 | 0.001342642 | 0.000236623 | Tnf/Il1b | 2 | 3.6412 | 87.1111 |
| GO:0006766 | vitamin metabolic process | 2/9 | 76/29008 | 0.000240982 | 0.00140872 | 0.000248268 | Tnf/Nfkb1 | 2 | 3.6180 | 84.8187 |
| GO:0002697 | regulation of immune effector process | 3/9 | 424/29008 | 0.00024396 | 0.001413485 | 0.000249108 | Tnf/Il6/Il1b | 3 | 3.6127 | 22.8050 |
| GO:0031331 | positive regulation of cellular catabolic process | 3/9 | 425/29008 | 0.000245656 | 0.001413485 | 0.000249108 | Tnf/Il6/Il1b | 3 | 3.6097 | 22.7514 |
| GO:0051090 | regulation of DNA-binding transcription factor activity | 3/9 | 425/29008 | 0.000245656 | 0.001413485 | 0.000249108 | Tnf/Il6/Il1b | 3 | 3.6097 | 22.7514 |
| GO:0032103 | positive regulation of response to external stimulus | 3/9 | 427/29008 | 0.000249071 | 0.001425669 | 0.000251255 | Tnf/Il6/Il1b | 3 | 3.6037 | 22.6448 |
| GO:0070997 | neuron death | 3/9 | 428/29008 | 0.00025079 | 0.001428071 | 0.000251679 | Tnf/Il6/Il1b | 3 | 3.6007 | 22.5919 |
| GO:0038034 | signal transduction in absence of ligand | 2/9 | 80/29008 | 0.000267022 | 0.00150491 | 0.00026522 | Tnf/Il1b | 2 | 3.5735 | 80.5778 |
| GO:0097192 | extrinsic apoptotic signaling pathway in absence of ligand | 2/9 | 80/29008 | 0.000267022 | 0.00150491 | 0.00026522 | Tnf/Il1b | 2 | 3.5735 | 80.5778 |
| GO:0002711 | positive regulation of T cell mediated immunity | 2/9 | 81/29008 | 0.000273738 | 0.001534889 | 0.000270504 | Il6/Il1b | 2 | 3.5627 | 79.5830 |
| GO:0050810 | regulation of steroid biosynthetic process | 2/9 | 82/29008 | 0.000280536 | 0.001565023 | 0.000275815 | Tnf/Nfkb1 | 2 | 3.5520 | 78.6125 |
| GO:1902893 | regulation of pri-miRNA transcription by RNA polymerase II | 2/9 | 83/29008 | 0.000287417 | 0.00159531 | 0.000281152 | Tnf/Nfkb1 | 2 | 3.5415 | 77.6653 |
| GO:0050767 | regulation of neurogenesis | 3/9 | 451/29008 | 0.000292484 | 0.001596289 | 0.000281325 | Tnf/Il6/Il1b | 3 | 3.5339 | 21.4398 |
| GO:0032729 | positive regulation of interferon-gamma production | 2/9 | 84/29008 | 0.00029438 | 0.001596289 | 0.000281325 | Tnf/Il1b | 2 | 3.5311 | 76.7407 |
| GO:0043154 | negative regulation of cysteine-type endopeptidase activity involved in apoptotic process | 2/9 | 84/29008 | 0.00029438 | 0.001596289 | 0.000281325 | Tnf/Il6 | 2 | 3.5311 | 76.7407 |
| GO:0050766 | positive regulation of phagocytosis | 2/9 | 84/29008 | 0.00029438 | 0.001596289 | 0.000281325 | Tnf/Il1b | 2 | 3.5311 | 76.7407 |
| GO:0010563 | negative regulation of phosphorus metabolic process | 3/9 | 453/29008 | 0.000296308 | 0.001596289 | 0.000281325 | Tnf/Il6/Il1b | 3 | 3.5283 | 21.3451 |
| GO:0045936 | negative regulation of phosphate metabolic process | 3/9 | 453/29008 | 0.000296308 | 0.001596289 | 0.000281325 | Tnf/Il6/Il1b | 3 | 3.5283 | 21.3451 |
| GO:0043525 | positive regulation of neuron apoptotic process | 2/9 | 85/29008 | 0.000301425 | 0.001608086 | 0.000283404 | Tnf/Il1b | 2 | 3.5208 | 75.8379 |
| GO:0061614 | pri-miRNA transcription by RNA polymerase II | 2/9 | 85/29008 | 0.000301425 | 0.001608086 | 0.000283404 | Tnf/Nfkb1 | 2 | 3.5208 | 75.8379 |
| GO:0022407 | regulation of cell-cell adhesion | 3/9 | 457/29008 | 0.000304055 | 0.001614281 | 0.000284496 | Tnf/Il6/Il1b | 3 | 3.5170 | 21.1583 |
| GO:0002637 | regulation of immunoglobulin production | 2/9 | 87/29008 | 0.000315761 | 0.001652483 | 0.000291228 | Tnf/Il6 | 2 | 3.5006 | 74.0945 |
| GO:0022904 | respiratory electron transport chain | 2/9 | 87/29008 | 0.000315761 | 0.001652483 | 0.000291228 | Ndufb8/Sdhb | 2 | 3.5006 | 74.0945 |
| GO:0046888 | negative regulation of hormone secretion | 2/9 | 87/29008 | 0.000315761 | 0.001652483 | 0.000291228 | Il6/Il1b | 2 | 3.5006 | 74.0945 |
| GO:0019915 | lipid storage | 2/9 | 89/29008 | 0.000330426 | 0.001721032 | 0.000303309 | Tnf/Il1b | 2 | 3.4809 | 72.4295 |
| GO:0045785 | positive regulation of cell adhesion | 3/9 | 471/29008 | 0.0003322 | 0.001722114 | 0.0003035 | Tnf/Il6/Il1b | 3 | 3.4786 | 20.5294 |
| GO:0010827 | regulation of glucose transmembrane transport | 2/9 | 90/29008 | 0.000337881 | 0.001743339 | 0.00030724 | Tnf/Il1b | 2 | 3.4712 | 71.6247 |
| GO:0002449 | lymphocyte mediated immunity | 3/9 | 477/29008 | 0.000344762 | 0.001765649 | 0.000311172 | Tnf/Il6/Il1b | 3 | 3.4625 | 20.2711 |
| GO:0030203 | glycosaminoglycan metabolic process | 2/9 | 91/29008 | 0.000345418 | 0.001765649 | 0.000311172 | Il1b/Nfkb1 | 2 | 3.4617 | 70.8376 |
| GO:0002460 | adaptive immune response based on somatic recombination of immune receptors built from immunoglobulin superfamily domains | 3/9 | 480/29008 | 0.000351157 | 0.001786675 | 0.000314878 | Tnf/Il6/Il1b | 3 | 3.4545 | 20.1444 |
| GO:0033108 | mitochondrial respiratory chain complex assembly | 2/9 | 92/29008 | 0.000353037 | 0.001787963 | 0.000315105 | Tfam/Ndufb8 | 2 | 3.4522 | 70.0676 |
| GO:0010717 | regulation of epithelial to mesenchymal transition | 2/9 | 93/29008 | 0.000360738 | 0.00181028 | 0.000319038 | Il6/Il1b | 2 | 3.4428 | 69.3142 |
| GO:0032651 | regulation of interleukin-1 beta production | 2/9 | 93/29008 | 0.000360738 | 0.00181028 | 0.000319038 | Tnf/Il6 | 2 | 3.4428 | 69.3142 |
| GO:0002532 | production of molecular mediator involved in inflammatory response | 2/9 | 94/29008 | 0.000368521 | 0.0018326 | 0.000322972 | Tnf/Il6 | 2 | 3.4335 | 68.5768 |
| GO:2000117 | negative regulation of cysteine-type endopeptidase activity | 2/9 | 94/29008 | 0.000368521 | 0.0018326 | 0.000322972 | Tnf/Il6 | 2 | 3.4335 | 68.5768 |
| GO:0022900 | electron transport chain | 2/9 | 95/29008 | 0.000376386 | 0.001863278 | 0.000328378 | Ndufb8/Sdhb | 2 | 3.4244 | 67.8550 |
| GO:0009896 | positive regulation of catabolic process | 3/9 | 494/29008 | 0.000382019 | 0.001882685 | 0.000331798 | Tnf/Il6/Il1b | 3 | 3.4179 | 19.5735 |
| GO:1901222 | regulation of NIK/NF-kappaB signaling | 2/9 | 96/29008 | 0.000384332 | 0.001885629 | 0.000332317 | Tnf/Il1b | 2 | 3.4153 | 67.1481 |
| GO:0046330 | positive regulation of JNK cascade | 2/9 | 97/29008 | 0.00039236 | 0.001916461 | 0.000337751 | Tnf/Il1b | 2 | 3.4063 | 66.4559 |
| GO:0001819 | positive regulation of cytokine production | 3/9 | 500/29008 | 0.000395766 | 0.001924541 | 0.000339175 | Tnf/Il6/Il1b | 3 | 3.4026 | 19.3387 |
| GO:0062014 | negative regulation of small molecule metabolic process | 2/9 | 98/29008 | 0.00040047 | 0.001938838 | 0.000341695 | Il6/Nfkb1 | 2 | 3.3974 | 65.7778 |
| GO:2001237 | negative regulation of extrinsic apoptotic signaling pathway | 2/9 | 103/29008 | 0.000442242 | 0.002131682 | 0.000375681 | Tnf/Il1b | 2 | 3.3543 | 62.5847 |
| GO:0042102 | positive regulation of T cell proliferation | 2/9 | 104/29008 | 0.00045084 | 0.002163641 | 0.000381313 | Il6/Il1b | 2 | 3.3460 | 61.9829 |
| GO:0048661 | positive regulation of smooth muscle cell proliferation | 2/9 | 105/29008 | 0.000459521 | 0.00219571 | 0.000386965 | Tnf/Il6 | 2 | 3.3377 | 61.3926 |
| GO:0046889 | positive regulation of lipid biosynthetic process | 2/9 | 106/29008 | 0.000468282 | 0.002227888 | 0.000392636 | Tnf/Il1b | 2 | 3.3295 | 60.8134 |
| GO:0006022 | aminoglycan metabolic process | 2/9 | 108/29008 | 0.000486049 | 0.002292567 | 0.000404035 | Il1b/Nfkb1 | 2 | 3.3133 | 59.6872 |
| GO:0043255 | regulation of carbohydrate biosynthetic process | 2/9 | 108/29008 | 0.000486049 | 0.002292567 | 0.000404035 | Il6/Nfkb1 | 2 | 3.3133 | 59.6872 |
| GO:0002709 | regulation of T cell mediated immunity | 2/9 | 111/29008 | 0.000513309 | 0.002410796 | 0.000424871 | Il6/Il1b | 2 | 3.2896 | 58.0741 |
| GO:0032652 | regulation of interleukin-1 production | 2/9 | 114/29008 | 0.000541298 | 0.002531432 | 0.000446131 | Tnf/Il6 | 2 | 3.2666 | 56.5458 |
| GO:0050764 | regulation of phagocytosis | 2/9 | 115/29008 | 0.00055079 | 0.002564907 | 0.000452031 | Tnf/Il1b | 2 | 3.2590 | 56.0541 |
| GO:0019218 | regulation of steroid metabolic process | 2/9 | 116/29008 | 0.000560363 | 0.002565994 | 0.000452222 | Tnf/Nfkb1 | 2 | 3.2515 | 55.5709 |
| GO:0038061 | NIK/NF-kappaB signaling | 2/9 | 116/29008 | 0.000560363 | 0.002565994 | 0.000452222 | Tnf/Il1b | 2 | 3.2515 | 55.5709 |
| GO:0051897 | positive regulation of protein kinase B signaling | 2/9 | 116/29008 | 0.000560363 | 0.002565994 | 0.000452222 | Tnf/Il6 | 2 | 3.2515 | 55.5709 |
| GO:1904659 | glucose transmembrane transport | 2/9 | 116/29008 | 0.000560363 | 0.002565994 | 0.000452222 | Tnf/Il1b | 2 | 3.2515 | 55.5709 |
| GO:0008645 | hexose transmembrane transport | 2/9 | 117/29008 | 0.000570016 | 0.002588627 | 0.000456211 | Tnf/Il1b | 2 | 3.2441 | 55.0959 |
| GO:0045446 | endothelial cell differentiation | 2/9 | 117/29008 | 0.000570016 | 0.002588627 | 0.000456211 | Tnf/Il1b | 2 | 3.2441 | 55.0959 |
| GO:0015749 | monosaccharide transmembrane transport | 2/9 | 119/29008 | 0.000589566 | 0.002666391 | 0.000469916 | Tnf/Il1b | 2 | 3.2295 | 54.1699 |
| GO:0034219 | carbohydrate transmembrane transport | 2/9 | 120/29008 | 0.000599462 | 0.002700037 | 0.000475846 | Tnf/Il1b | 2 | 3.2222 | 53.7185 |
| GO:0032649 | regulation of interferon-gamma production | 2/9 | 123/29008 | 0.000629635 | 0.002824362 | 0.000497756 | Tnf/Il1b | 2 | 3.2009 | 52.4083 |
| GO:0002702 | positive regulation of production of molecular mediator of immune response | 2/9 | 125/29008 | 0.000650153 | 0.002904546 | 0.000511888 | Il6/Il1b | 2 | 3.1870 | 51.5698 |
| GO:0043123 | positive regulation of I-kappaB kinase/NF-kappaB signaling | 2/9 | 126/29008 | 0.000660533 | 0.002938971 | 0.000517955 | Tnf/Il1b | 2 | 3.1801 | 51.1605 |
| GO:1901216 | positive regulation of neuron death | 2/9 | 129/29008 | 0.000692156 | 0.003067256 | 0.000540563 | Tnf/Il1b | 2 | 3.1598 | 49.9707 |
| GO:0033138 | positive regulation of peptidyl-serine phosphorylation | 2/9 | 130/29008 | 0.000702858 | 0.003089762 | 0.00054453 | Tnf/Il6 | 2 | 3.1531 | 49.5863 |
| GO:0043406 | positive regulation of MAP kinase activity | 2/9 | 130/29008 | 0.000702858 | 0.003089762 | 0.00054453 | Tnf/Il1b | 2 | 3.1531 | 49.5863 |
| GO:0032874 | positive regulation of stress-activated MAPK cascade | 2/9 | 131/29008 | 0.00071364 | 0.003112262 | 0.000548495 | Tnf/Il1b | 2 | 3.1465 | 49.2078 |
| GO:0045727 | positive regulation of translation | 2/9 | 131/29008 | 0.00071364 | 0.003112262 | 0.000548495 | Tnf/Il6 | 2 | 3.1465 | 49.2078 |
| GO:0003158 | endothelium development | 2/9 | 133/29008 | 0.000735445 | 0.003182102 | 0.000560803 | Tnf/Il1b | 2 | 3.1334 | 48.4678 |
| GO:0070304 | positive regulation of stress-activated protein kinase signaling cascade | 2/9 | 133/29008 | 0.000735445 | 0.003182102 | 0.000560803 | Tnf/Il1b | 2 | 3.1334 | 48.4678 |
| GO:0032609 | interferon-gamma production | 2/9 | 135/29008 | 0.000757571 | 0.003264982 | 0.00057541 | Tnf/Il1b | 2 | 3.1206 | 47.7498 |
| GO:0034763 | negative regulation of transmembrane transport | 2/9 | 140/29008 | 0.000814288 | 0.003495712 | 0.000616073 | Tnf/Il1b | 2 | 3.0892 | 46.0444 |
| GO:0046328 | regulation of JNK cascade | 2/9 | 143/29008 | 0.000849278 | 0.003617659 | 0.000637565 | Tnf/Il1b | 2 | 3.0710 | 45.0785 |
| GO:0050671 | positive regulation of lymphocyte proliferation | 2/9 | 143/29008 | 0.000849278 | 0.003617659 | 0.000637565 | Il6/Il1b | 2 | 3.0710 | 45.0785 |
| GO:0002456 | T cell mediated immunity | 2/9 | 145/29008 | 0.000873003 | 0.003690118 | 0.000650334 | Il6/Il1b | 2 | 3.0590 | 44.4567 |
| GO:0032946 | positive regulation of mononuclear cell proliferation | 2/9 | 145/29008 | 0.000873003 | 0.003690118 | 0.000650334 | Il6/Il1b | 2 | 3.0590 | 44.4567 |
| GO:0001837 | epithelial to mesenchymal transition | 2/9 | 152/29008 | 0.000958554 | 0.004020803 | 0.000708613 | Il6/Il1b | 2 | 3.0184 | 42.4094 |
| GO:0051092 | positive regulation of NF-kappaB transcription factor activity | 2/9 | 152/29008 | 0.000958554 | 0.004020803 | 0.000708613 | Tnf/Il1b | 2 | 3.0184 | 42.4094 |
| GO:0007043 | cell-cell junction assembly | 2/9 | 153/29008 | 0.000971093 | 0.004057914 | 0.000715154 | Tnf/Il1b | 2 | 3.0127 | 42.1322 |
| GO:0022612 | gland morphogenesis | 2/9 | 155/29008 | 0.000996411 | 0.004147938 | 0.000731019 | Tnf/Il6 | 2 | 3.0016 | 41.5885 |
| GO:0008643 | carbohydrate transport | 2/9 | 156/29008 | 0.001009189 | 0.004185277 | 0.0007376 | Tnf/Il1b | 2 | 2.9960 | 41.3219 |
| GO:0030856 | regulation of epithelial cell differentiation | 2/9 | 157/29008 | 0.001022046 | 0.004206848 | 0.000741401 | Tnf/Il1b | 2 | 2.9905 | 41.0587 |
| GO:0070665 | positive regulation of leukocyte proliferation | 2/9 | 157/29008 | 0.001022046 | 0.004206848 | 0.000741401 | Il6/Il1b | 2 | 2.9905 | 41.0587 |
| GO:0045667 | regulation of osteoblast differentiation | 2/9 | 158/29008 | 0.001034982 | 0.0042442 | 0.000747984 | Tnf/Il6 | 2 | 2.9851 | 40.7989 |
| GO:0034250 | positive regulation of cellular amide metabolic process | 2/9 | 159/29008 | 0.001047998 | 0.004281598 | 0.000754575 | Tnf/Il6 | 2 | 2.9796 | 40.5423 |
| GO:0009060 | aerobic respiration | 2/9 | 161/29008 | 0.001074267 | 0.004372665 | 0.000770624 | Ndufb8/Sdhb | 2 | 2.9689 | 40.0386 |
| GO:0033135 | regulation of peptidyl-serine phosphorylation | 2/9 | 166/29008 | 0.001141323 | 0.00461145 | 0.000812707 | Tnf/Il6 | 2 | 2.9426 | 38.8327 |
| GO:0062013 | positive regulation of small molecule metabolic process | 2/9 | 166/29008 | 0.001141323 | 0.00461145 | 0.000812707 | Tnf/Il1b | 2 | 2.9426 | 38.8327 |
| GO:0006694 | steroid biosynthetic process | 2/9 | 168/29008 | 0.001168699 | 0.004687591 | 0.000826126 | Tnf/Nfkb1 | 2 | 2.9323 | 38.3704 |
| GO:2001236 | regulation of extrinsic apoptotic signaling pathway | 2/9 | 168/29008 | 0.001168699 | 0.004687591 | 0.000826126 | Tnf/Il1b | 2 | 2.9323 | 38.3704 |
| GO:1903531 | negative regulation of secretion by cell | 2/9 | 170/29008 | 0.001196389 | 0.004781207 | 0.000842624 | Il6/Il1b | 2 | 2.9221 | 37.9190 |
| GO:0051896 | regulation of protein kinase B signaling | 2/9 | 173/29008 | 0.001238516 | 0.004931627 | 0.000869134 | Tnf/Il6 | 2 | 2.9071 | 37.2614 |
| GO:0048660 | regulation of smooth muscle cell proliferation | 2/9 | 178/29008 | 0.0013103 | 0.005198626 | 0.000916189 | Tnf/Il6 | 2 | 2.8826 | 36.2147 |
| GO:0042129 | regulation of T cell proliferation | 2/9 | 183/29008 | 0.001384045 | 0.005451846 | 0.000960816 | Il6/Il1b | 2 | 2.8588 | 35.2253 |
| GO:0045834 | positive regulation of lipid metabolic process | 2/9 | 183/29008 | 0.001384045 | 0.005451846 | 0.000960816 | Tnf/Il1b | 2 | 2.8588 | 35.2253 |
| GO:0048659 | smooth muscle cell proliferation | 2/9 | 184/29008 | 0.001399029 | 0.005491187 | 0.000967749 | Tnf/Il6 | 2 | 2.8542 | 35.0338 |
| GO:0010951 | negative regulation of endopeptidase activity | 2/9 | 187/29008 | 0.00144445 | 0.005648806 | 0.000995527 | Tnf/Il6 | 2 | 2.8403 | 34.4718 |
| GO:0001659 | temperature homeostasis | 2/9 | 188/29008 | 0.001459746 | 0.005648806 | 0.000995527 | Tnf/Il1b | 2 | 2.8357 | 34.2884 |
| GO:1902107 | positive regulation of leukocyte differentiation | 2/9 | 188/29008 | 0.001459746 | 0.005648806 | 0.000995527 | Tnf/Il6 | 2 | 2.8357 | 34.2884 |
| GO:1903708 | positive regulation of hemopoiesis | 2/9 | 188/29008 | 0.001459746 | 0.005648806 | 0.000995527 | Tnf/Il6 | 2 | 2.8357 | 34.2884 |
| GO:0043122 | regulation of I-kappaB kinase/NF-kappaB signaling | 2/9 | 190/29008 | 0.001490573 | 0.005747861 | 0.001012984 | Tnf/Il1b | 2 | 2.8266 | 33.9275 |
| GO:0006109 | regulation of carbohydrate metabolic process | 2/9 | 197/29008 | 0.001600924 | 0.006149073 | 0.001083693 | Il6/Nfkb1 | 2 | 2.7956 | 32.7219 |
| GO:0016051 | carbohydrate biosynthetic process | 2/9 | 198/29008 | 0.001616999 | 0.006149073 | 0.001083693 | Il6/Nfkb1 | 2 | 2.7913 | 32.5567 |
| GO:0032872 | regulation of stress-activated MAPK cascade | 2/9 | 198/29008 | 0.001616999 | 0.006149073 | 0.001083693 | Tnf/Il1b | 2 | 2.7913 | 32.5567 |
| GO:0043405 | regulation of MAP kinase activity | 2/9 | 198/29008 | 0.001616999 | 0.006149073 | 0.001083693 | Tnf/Il1b | 2 | 2.7913 | 32.5567 |
| GO:0051048 | negative regulation of secretion | 2/9 | 199/29008 | 0.001633152 | 0.006189084 | 0.001090744 | Il6/Il1b | 2 | 2.7870 | 32.3931 |
| GO:0070302 | regulation of stress-activated protein kinase signaling cascade | 2/9 | 201/29008 | 0.001665691 | 0.006290704 | 0.001108653 | Tnf/Il1b | 2 | 2.7784 | 32.0708 |
| GO:0006469 | negative regulation of protein kinase activity | 2/9 | 203/29008 | 0.001698541 | 0.006392796 | 0.001126646 | Il6/Il1b | 2 | 2.7699 | 31.7548 |
| GO:0009743 | response to carbohydrate | 2/9 | 204/29008 | 0.001715082 | 0.00643302 | 0.001133735 | Il1b/Nfkb1 | 2 | 2.7657 | 31.5991 |
| GO:0071902 | positive regulation of protein serine/threonine kinase activity | 2/9 | 206/29008 | 0.001748396 | 0.006535671 | 0.001151826 | Tnf/Il1b | 2 | 2.7574 | 31.2923 |
| GO:0043281 | regulation of cysteine-type endopeptidase activity involved in apoptotic process | 2/9 | 210/29008 | 0.001815952 | 0.006765192 | 0.001192276 | Tnf/Il6 | 2 | 2.7409 | 30.6963 |
| GO:0045216 | cell-cell junction organization | 2/9 | 213/29008 | 0.001867431 | 0.006933467 | 0.001221932 | Tnf/Il1b | 2 | 2.7288 | 30.2640 |
| GO:0045333 | cellular respiration | 2/9 | 215/29008 | 0.001902135 | 0.00703854 | 0.00124045 | Ndufb8/Sdhb | 2 | 2.7208 | 29.9824 |
| GO:0050870 | positive regulation of T cell activation | 2/9 | 216/29008 | 0.001919603 | 0.00707934 | 0.00124764 | Il6/Il1b | 2 | 2.7168 | 29.8436 |
| GO:0001649 | osteoblast differentiation | 2/9 | 220/29008 | 0.001990243 | 0.007290923 | 0.001284929 | Tnf/Il6 | 2 | 2.7011 | 29.3010 |
| GO:1901888 | regulation of cell junction assembly | 2/9 | 220/29008 | 0.001990243 | 0.007290923 | 0.001284929 | Tnf/Il1b | 2 | 2.7011 | 29.3010 |
| GO:0042098 | T cell proliferation | 2/9 | 221/29008 | 0.002008095 | 0.007307604 | 0.001287869 | Il6/Il1b | 2 | 2.6972 | 29.1684 |
| GO:0045732 | positive regulation of protein catabolic process | 2/9 | 221/29008 | 0.002008095 | 0.007307604 | 0.001287869 | Tnf/Il1b | 2 | 2.6972 | 29.1684 |
| GO:0006006 | glucose metabolic process | 2/9 | 225/29008 | 0.002080271 | 0.007545275 | 0.001329755 | Tnf/Il6 | 2 | 2.6819 | 28.6499 |
| GO:0033673 | negative regulation of kinase activity | 2/9 | 226/29008 | 0.002098507 | 0.007561506 | 0.001332615 | Il6/Il1b | 2 | 2.6781 | 28.5231 |
| GO:0061138 | morphogenesis of a branching epithelium | 2/9 | 226/29008 | 0.002098507 | 0.007561506 | 0.001332615 | Tnf/Il6 | 2 | 2.6781 | 28.5231 |
| GO:0070374 | positive regulation of ERK1 and ERK2 cascade | 2/9 | 227/29008 | 0.00211682 | 0.007602564 | 0.001339851 | Il6/Il1b | 2 | 2.6743 | 28.3975 |
| GO:0007249 | I-kappaB kinase/NF-kappaB signaling | 2/9 | 228/29008 | 0.002135208 | 0.007643629 | 0.001347089 | Tnf/Il1b | 2 | 2.6706 | 28.2729 |
| GO:1901617 | organic hydroxy compound biosynthetic process | 2/9 | 230/29008 | 0.002172216 | 0.007750861 | 0.001365987 | Tnf/Nfkb1 | 2 | 2.6631 | 28.0271 |
| GO:0048762 | mesenchymal cell differentiation | 2/9 | 237/29008 | 0.002304147 | 0.008195009 | 0.001444262 | Il6/Il1b | 2 | 2.6375 | 27.1992 |
| GO:0097191 | extrinsic apoptotic signaling pathway | 2/9 | 238/29008 | 0.0023233 | 0.008236472 | 0.001451569 | Tnf/Il1b | 2 | 2.6339 | 27.0850 |
| GO:2000116 | regulation of cysteine-type endopeptidase activity | 2/9 | 239/29008 | 0.002342528 | 0.008277937 | 0.001458877 | Tnf/Il6 | 2 | 2.6303 | 26.9716 |
| GO:0045664 | regulation of neuron differentiation | 2/9 | 241/29008 | 0.002381213 | 0.008360873 | 0.001473493 | Il6/Il1b | 2 | 2.6232 | 26.7478 |
| GO:0050670 | regulation of lymphocyte proliferation | 2/9 | 241/29008 | 0.002381213 | 0.008360873 | 0.001473493 | Il6/Il1b | 2 | 2.6232 | 26.7478 |
| GO:2001234 | negative regulation of apoptotic signaling pathway | 2/9 | 242/29008 | 0.00240067 | 0.008402345 | 0.001480802 | Tnf/Il1b | 2 | 2.6197 | 26.6373 |
| GO:0032944 | regulation of mononuclear cell proliferation | 2/9 | 245/29008 | 0.002459496 | 0.008580907 | 0.001512271 | Il6/Il1b | 2 | 2.6092 | 26.3111 |
| GO:0033002 | muscle cell proliferation | 2/9 | 255/29008 | 0.002660506 | 0.009194643 | 0.001620434 | Tnf/Il6 | 2 | 2.5750 | 25.2793 |
| GO:0051607 | defense response to virus | 2/9 | 255/29008 | 0.002660506 | 0.009194643 | 0.001620434 | Il6/Il1b | 2 | 2.5750 | 25.2793 |
| GO:0140546 | defense response to symbiont | 2/9 | 255/29008 | 0.002660506 | 0.009194643 | 0.001620434 | Il6/Il1b | 2 | 2.5750 | 25.2793 |
| GO:0019318 | hexose metabolic process | 2/9 | 256/29008 | 0.002681023 | 0.009236503 | 0.001627811 | Tnf/Il6 | 2 | 2.5717 | 25.1806 |
| GO:0051348 | negative regulation of transferase activity | 2/9 | 259/29008 | 0.002743026 | 0.009420581 | 0.001660253 | Il6/Il1b | 2 | 2.5618 | 24.8889 |
| GO:0010466 | negative regulation of peptidase activity | 2/9 | 261/29008 | 0.002784738 | 0.009504433 | 0.001675031 | Tnf/Il6 | 2 | 2.5552 | 24.6982 |
| GO:0070663 | regulation of leukocyte proliferation | 2/9 | 261/29008 | 0.002784738 | 0.009504433 | 0.001675031 | Il6/Il1b | 2 | 2.5552 | 24.6982 |
| GO:0071375 | cellular response to peptide hormone stimulus | 2/9 | 264/29008 | 0.002847871 | 0.009689814 | 0.001707702 | Il1b/Nfkb1 | 2 | 2.5455 | 24.4175 |
| GO:0002064 | epithelial cell development | 2/9 | 266/29008 | 0.002890335 | 0.009803944 | 0.001727815 | Tnf/Il1b | 2 | 2.5391 | 24.2339 |
| GO:0005996 | monosaccharide metabolic process | 2/9 | 273/29008 | 0.003041321 | 0.010284346 | 0.00181248 | Tnf/Il6 | 2 | 2.5169 | 23.6125 |
| GO:0010692 | regulation of alkaline phosphatase activity | 1/9 | 10/29008 | 0.003098744 | 0.010316498 | 0.001818146 | Tnf | 1 | 2.5088 | 322.3111 |
| GO:0035744 | T-helper 1 cell cytokine production | 1/9 | 10/29008 | 0.003098744 | 0.010316498 | 0.001818146 | Il1b | 1 | 2.5088 | 322.3111 |
| GO:0051971 | positive regulation of transmission of nerve impulse | 1/9 | 10/29008 | 0.003098744 | 0.010316498 | 0.001818146 | Il6 | 1 | 2.5088 | 322.3111 |
| GO:0061043 | regulation of vascular wound healing | 1/9 | 10/29008 | 0.003098744 | 0.010316498 | 0.001818146 | Tnf | 1 | 2.5088 | 322.3111 |
| GO:0090091 | positive regulation of extracellular matrix disassembly | 1/9 | 10/29008 | 0.003098744 | 0.010316498 | 0.001818146 | Il6 | 1 | 2.5088 | 322.3111 |
| GO:0043523 | regulation of neuron apoptotic process | 2/9 | 276/29008 | 0.003107153 | 0.010316498 | 0.001818146 | Tnf/Il1b | 2 | 2.5076 | 23.3559 |
| GO:0002377 | immunoglobulin production | 2/9 | 277/29008 | 0.003129246 | 0.010327451 | 0.001820077 | Tnf/Il6 | 2 | 2.5046 | 23.2716 |
| GO:1903829 | positive regulation of cellular protein localization | 2/9 | 277/29008 | 0.003129246 | 0.010327451 | 0.001820077 | Tnf/Il6 | 2 | 2.5046 | 23.2716 |
| GO:0002676 | regulation of chronic inflammatory response | 1/9 | 11/29008 | 0.003408149 | 0.010951917 | 0.00193013 | Tnf | 1 | 2.4675 | 293.0101 |
| GO:0014889 | muscle atrophy | 1/9 | 11/29008 | 0.003408149 | 0.010951917 | 0.00193013 | Fbxo32 | 1 | 2.4675 | 293.0101 |
| GO:0031053 | primary miRNA processing | 1/9 | 11/29008 | 0.003408149 | 0.010951917 | 0.00193013 | Il6 | 1 | 2.4675 | 293.0101 |
| GO:0032353 | negative regulation of hormone biosynthetic process | 1/9 | 11/29008 | 0.003408149 | 0.010951917 | 0.00193013 | Nfkb1 | 1 | 2.4675 | 293.0101 |
| GO:0033084 | regulation of immature T cell proliferation in thymus | 1/9 | 11/29008 | 0.003408149 | 0.010951917 | 0.00193013 | Il1b | 1 | 2.4675 | 293.0101 |
| GO:0060693 | regulation of branching involved in salivary gland morphogenesis | 1/9 | 11/29008 | 0.003408149 | 0.010951917 | 0.00193013 | Tnf | 1 | 2.4675 | 293.0101 |
| GO:0071864 | positive regulation of cell proliferation in bone marrow | 1/9 | 11/29008 | 0.003408149 | 0.010951917 | 0.00193013 | Il6 | 1 | 2.4675 | 293.0101 |
| GO:0097421 | liver regeneration | 1/9 | 11/29008 | 0.003408149 | 0.010951917 | 0.00193013 | Il6 | 1 | 2.4675 | 293.0101 |
| GO:2001280 | positive regulation of unsaturated fatty acid biosynthetic process | 1/9 | 11/29008 | 0.003408149 | 0.010951917 | 0.00193013 | Il1b | 1 | 2.4675 | 293.0101 |
| GO:0060485 | mesenchyme development | 2/9 | 293/29008 | 0.003492863 | 0.011191418 | 0.001972339 | Il6/Il1b | 2 | 2.4568 | 22.0008 |
| GO:0030217 | T cell differentiation | 2/9 | 299/29008 | 0.003634114 | 0.011540953 | 0.00203394 | Il6/Il1b | 2 | 2.4396 | 21.5593 |
| GO:0045765 | regulation of angiogenesis | 2/9 | 302/29008 | 0.003705737 | 0.011540953 | 0.00203394 | Tnf/Il1b | 2 | 2.4311 | 21.3451 |
| GO:0001781 | neutrophil apoptotic process | 1/9 | 12/29008 | 0.003717468 | 0.011540953 | 0.00203394 | Il6 | 1 | 2.4298 | 268.5926 |
| GO:0014874 | response to stimulus involved in regulation of muscle adaptation | 1/9 | 12/29008 | 0.003717468 | 0.011540953 | 0.00203394 | Fbxo32 | 1 | 2.4298 | 268.5926 |
| GO:0031392 | regulation of prostaglandin biosynthetic process | 1/9 | 12/29008 | 0.003717468 | 0.011540953 | 0.00203394 | Il1b | 1 | 2.4298 | 268.5926 |
| GO:0032351 | negative regulation of hormone metabolic process | 1/9 | 12/29008 | 0.003717468 | 0.011540953 | 0.00203394 | Nfkb1 | 1 | 2.4298 | 268.5926 |
| GO:0033080 | immature T cell proliferation in thymus | 1/9 | 12/29008 | 0.003717468 | 0.011540953 | 0.00203394 | Il1b | 1 | 2.4298 | 268.5926 |
| GO:0034112 | positive regulation of homotypic cell-cell adhesion | 1/9 | 12/29008 | 0.003717468 | 0.011540953 | 0.00203394 | Il6 | 1 | 2.4298 | 268.5926 |
| GO:0051798 | positive regulation of hair follicle development | 1/9 | 12/29008 | 0.003717468 | 0.011540953 | 0.00203394 | Tnf | 1 | 2.4298 | 268.5926 |
| GO:1903800 | positive regulation of production of miRNAs involved in gene silencing by miRNA | 1/9 | 12/29008 | 0.003717468 | 0.011540953 | 0.00203394 | Il6 | 1 | 2.4298 | 268.5926 |
| GO:2000551 | regulation of T-helper 2 cell cytokine production | 1/9 | 12/29008 | 0.003717468 | 0.011540953 | 0.00203394 | Il6 | 1 | 2.4298 | 268.5926 |
| GO:0015980 | energy derivation by oxidation of organic compounds | 2/9 | 305/29008 | 0.003778023 | 0.011695908 | 0.002061249 | Ndufb8/Sdhb | 2 | 2.4227 | 21.1352 |
| GO:1901342 | regulation of vasculature development | 2/9 | 306/29008 | 0.003802266 | 0.011737893 | 0.002068648 | Tnf/Il1b | 2 | 2.4200 | 21.0661 |
| GO:1901653 | cellular response to peptide | 2/9 | 308/29008 | 0.003850971 | 0.011832474 | 0.002085317 | Il1b/Nfkb1 | 2 | 2.4144 | 20.9293 |
| GO:0051402 | neuron apoptotic process | 2/9 | 309/29008 | 0.003875434 | 0.011832474 | 0.002085317 | Tnf/Il1b | 2 | 2.4117 | 20.8616 |
| GO:0009895 | negative regulation of catabolic process | 2/9 | 311/29008 | 0.003924581 | 0.011832474 | 0.002085317 | Tnf/Il1b | 2 | 2.4062 | 20.7274 |
| GO:0030198 | extracellular matrix organization | 2/9 | 311/29008 | 0.003924581 | 0.011832474 | 0.002085317 | Tnf/Il6 | 2 | 2.4062 | 20.7274 |
| GO:0043062 | extracellular structure organization | 2/9 | 312/29008 | 0.003949264 | 0.011832474 | 0.002085317 | Tnf/Il6 | 2 | 2.4035 | 20.6610 |
| GO:0045229 | external encapsulating structure organization | 2/9 | 313/29008 | 0.003974021 | 0.011832474 | 0.002085317 | Tnf/Il6 | 2 | 2.4008 | 20.5950 |
| GO:0032725 | positive regulation of granulocyte macrophage colony-stimulating factor production | 1/9 | 13/29008 | 0.004026702 | 0.011832474 | 0.002085317 | Il1b | 1 | 2.3951 | 247.9316 |
| GO:0033083 | regulation of immature T cell proliferation | 1/9 | 13/29008 | 0.004026702 | 0.011832474 | 0.002085317 | Il1b | 1 | 2.3951 | 247.9316 |
| GO:0045628 | regulation of T-helper 2 cell differentiation | 1/9 | 13/29008 | 0.004026702 | 0.011832474 | 0.002085317 | Il6 | 1 | 2.3951 | 247.9316 |
| GO:0048711 | positive regulation of astrocyte differentiation | 1/9 | 13/29008 | 0.004026702 | 0.011832474 | 0.002085317 | Il1b | 1 | 2.3951 | 247.9316 |
| GO:0070091 | glucagon secretion | 1/9 | 13/29008 | 0.004026702 | 0.011832474 | 0.002085317 | Il6 | 1 | 2.3951 | 247.9316 |
| GO:0070092 | regulation of glucagon secretion | 1/9 | 13/29008 | 0.004026702 | 0.011832474 | 0.002085317 | Il6 | 1 | 2.3951 | 247.9316 |
| GO:0071803 | positive regulation of podosome assembly | 1/9 | 13/29008 | 0.004026702 | 0.011832474 | 0.002085317 | Tnf | 1 | 2.3951 | 247.9316 |
| GO:0071863 | regulation of cell proliferation in bone marrow | 1/9 | 13/29008 | 0.004026702 | 0.011832474 | 0.002085317 | Il6 | 1 | 2.3951 | 247.9316 |
| GO:0072540 | T-helper 17 cell lineage commitment | 1/9 | 13/29008 | 0.004026702 | 0.011832474 | 0.002085317 | Il6 | 1 | 2.3951 | 247.9316 |
| GO:1902510 | regulation of apoptotic DNA fragmentation | 1/9 | 13/29008 | 0.004026702 | 0.011832474 | 0.002085317 | Il6 | 1 | 2.3951 | 247.9316 |
| GO:1902931 | negative regulation of alcohol biosynthetic process | 1/9 | 13/29008 | 0.004026702 | 0.011832474 | 0.002085317 | Nfkb1 | 1 | 2.3951 | 247.9316 |
| GO:2000343 | positive regulation of chemokine (C-X-C motif) ligand 2 production | 1/9 | 13/29008 | 0.004026702 | 0.011832474 | 0.002085317 | Tnf | 1 | 2.3951 | 247.9316 |
| GO:0051222 | positive regulation of protein transport | 2/9 | 317/29008 | 0.00407378 | 0.011938891 | 0.002104072 | Tnf/Il6 | 2 | 2.3900 | 20.3351 |
| GO:1902105 | regulation of leukocyte differentiation | 2/9 | 320/29008 | 0.004149367 | 0.012128071 | 0.002137412 | Tnf/Il6 | 2 | 2.3820 | 20.1444 |
| GO:0070372 | regulation of ERK1 and ERK2 cascade | 2/9 | 326/29008 | 0.004302513 | 0.012441513 | 0.002192652 | Il6/Il1b | 2 | 2.3663 | 19.7737 |
| GO:0032769 | negative regulation of monooxygenase activity | 1/9 | 14/29008 | 0.00433585 | 0.012441513 | 0.002192652 | Nfkb1 | 1 | 2.3629 | 230.2222 |
| GO:0033079 | immature T cell proliferation | 1/9 | 14/29008 | 0.00433585 | 0.012441513 | 0.002192652 | Il1b | 1 | 2.3629 | 230.2222 |
| GO:0045188 | regulation of circadian sleep/wake cycle, non-REM sleep | 1/9 | 14/29008 | 0.00433585 | 0.012441513 | 0.002192652 | Il6 | 1 | 2.3629 | 230.2222 |
| GO:0048755 | branching morphogenesis of a nerve | 1/9 | 14/29008 | 0.00433585 | 0.012441513 | 0.002192652 | Il1b | 1 | 2.3629 | 230.2222 |
| GO:0071838 | cell proliferation in bone marrow | 1/9 | 14/29008 | 0.00433585 | 0.012441513 | 0.002192652 | Il6 | 1 | 2.3629 | 230.2222 |
| GO:2001279 | regulation of unsaturated fatty acid biosynthetic process | 1/9 | 14/29008 | 0.00433585 | 0.012441513 | 0.002192652 | Il1b | 1 | 2.3629 | 230.2222 |
| GO:0016042 | lipid catabolic process | 2/9 | 328/29008 | 0.004354144 | 0.012461471 | 0.002196169 | Tnf/Il1b | 2 | 2.3611 | 19.6531 |
| GO:0046651 | lymphocyte proliferation | 2/9 | 330/29008 | 0.004406067 | 0.012577318 | 0.002216586 | Il6/Il1b | 2 | 2.3559 | 19.5340 |
| GO:1904951 | positive regulation of establishment of protein localization | 2/9 | 333/29008 | 0.004484495 | 0.012768031 | 0.002250197 | Tnf/Il6 | 2 | 2.3483 | 19.3580 |
| GO:0032943 | mononuclear cell proliferation | 2/9 | 334/29008 | 0.004510783 | 0.012809691 | 0.002257539 | Il6/Il1b | 2 | 2.3457 | 19.3001 |
| GO:0051235 | maintenance of location | 2/9 | 335/29008 | 0.004537143 | 0.012851341 | 0.002264879 | Tnf/Il1b | 2 | 2.3432 | 19.2425 |
| GO:0008202 | steroid metabolic process | 2/9 | 337/29008 | 0.004590081 | 0.012858337 | 0.002266112 | Tnf/Nfkb1 | 2 | 2.3382 | 19.1283 |
| GO:0050863 | regulation of T cell activation | 2/9 | 339/29008 | 0.004643309 | 0.012858337 | 0.002266112 | Il6/Il1b | 2 | 2.3332 | 19.0154 |
| GO:0010715 | regulation of extracellular matrix disassembly | 1/9 | 15/29008 | 0.004644913 | 0.012858337 | 0.002266112 | Il6 | 1 | 2.3330 | 214.8741 |
| GO:0016264 | gap junction assembly | 1/9 | 15/29008 | 0.004644913 | 0.012858337 | 0.002266112 | Il1b | 1 | 2.3330 | 214.8741 |
| GO:0032308 | positive regulation of prostaglandin secretion | 1/9 | 15/29008 | 0.004644913 | 0.012858337 | 0.002266112 | Il1b | 1 | 2.3330 | 214.8741 |
| GO:0032645 | regulation of granulocyte macrophage colony-stimulating factor production | 1/9 | 15/29008 | 0.004644913 | 0.012858337 | 0.002266112 | Il1b | 1 | 2.3330 | 214.8741 |
| GO:0035745 | T-helper 2 cell cytokine production | 1/9 | 15/29008 | 0.004644913 | 0.012858337 | 0.002266112 | Il6 | 1 | 2.3330 | 214.8741 |
| GO:0042748 | circadian sleep/wake cycle, non-REM sleep | 1/9 | 15/29008 | 0.004644913 | 0.012858337 | 0.002266112 | Il6 | 1 | 2.3330 | 214.8741 |
| GO:1903624 | regulation of DNA catabolic process | 1/9 | 15/29008 | 0.004644913 | 0.012858337 | 0.002266112 | Il6 | 1 | 2.3330 | 214.8741 |
| GO:0018105 | peptidyl-serine phosphorylation | 2/9 | 340/29008 | 0.004670031 | 0.012895388 | 0.002272642 | Tnf/Il6 | 2 | 2.3307 | 18.9595 |
| GO:0001933 | negative regulation of protein phosphorylation | 2/9 | 349/29008 | 0.004913781 | 0.013416818 | 0.002364537 | Il6/Il1b | 2 | 2.3086 | 18.4706 |
| GO:0070371 | ERK1 and ERK2 cascade | 2/9 | 349/29008 | 0.004913781 | 0.013416818 | 0.002364537 | Il6/Il1b | 2 | 2.3086 | 18.4706 |
| GO:0071900 | regulation of protein serine/threonine kinase activity | 2/9 | 349/29008 | 0.004913781 | 0.013416818 | 0.002364537 | Tnf/Il1b | 2 | 2.3086 | 18.4706 |
| GO:0032604 | granulocyte macrophage colony-stimulating factor production | 1/9 | 16/29008 | 0.004953891 | 0.013416818 | 0.002364537 | Il1b | 1 | 2.3051 | 201.4444 |
| GO:0035729 | cellular response to hepatocyte growth factor stimulus | 1/9 | 16/29008 | 0.004953891 | 0.013416818 | 0.002364537 | Il6 | 1 | 2.3051 | 201.4444 |
| GO:0061042 | vascular wound healing | 1/9 | 16/29008 | 0.004953891 | 0.013416818 | 0.002364537 | Tnf | 1 | 2.3051 | 201.4444 |
| GO:2000628 | regulation of miRNA metabolic process | 1/9 | 16/29008 | 0.004953891 | 0.013416818 | 0.002364537 | Nfkb1 | 1 | 2.3051 | 201.4444 |
| GO:0043434 | response to peptide hormone | 2/9 | 351/29008 | 0.00496874 | 0.013416818 | 0.002364537 | Il1b/Nfkb1 | 2 | 2.3038 | 18.3653 |
| GO:0045862 | positive regulation of proteolysis | 2/9 | 351/29008 | 0.00496874 | 0.013416818 | 0.002364537 | Tnf/Il1b | 2 | 2.3038 | 18.3653 |
| GO:0052548 | regulation of endopeptidase activity | 2/9 | 357/29008 | 0.00513534 | 0.013705686 | 0.002415446 | Tnf/Il6 | 2 | 2.2894 | 18.0566 |
| GO:0045860 | positive regulation of protein kinase activity | 2/9 | 358/29008 | 0.005163358 | 0.013705686 | 0.002415446 | Tnf/Il1b | 2 | 2.2871 | 18.0062 |
| GO:0045861 | negative regulation of proteolysis | 2/9 | 358/29008 | 0.005163358 | 0.013705686 | 0.002415446 | Tnf/Il6 | 2 | 2.2871 | 18.0062 |
| GO:0070661 | leukocyte proliferation | 2/9 | 358/29008 | 0.005163358 | 0.013705686 | 0.002415446 | Il6/Il1b | 2 | 2.2871 | 18.0062 |
| GO:0042060 | wound healing | 2/9 | 361/29008 | 0.005247841 | 0.013705686 | 0.002415446 | Tnf/Il6 | 2 | 2.2800 | 17.8566 |
| GO:0002295 | T-helper cell lineage commitment | 1/9 | 17/29008 | 0.005262784 | 0.013705686 | 0.002415446 | Il6 | 1 | 2.2788 | 189.5948 |
| GO:0010713 | negative regulation of collagen metabolic process | 1/9 | 17/29008 | 0.005262784 | 0.013705686 | 0.002415446 | Il6 | 1 | 2.2788 | 189.5948 |
| GO:0032306 | regulation of prostaglandin secretion | 1/9 | 17/29008 | 0.005262784 | 0.013705686 | 0.002415446 | Il1b | 1 | 2.2788 | 189.5948 |
| GO:0032966 | negative regulation of collagen biosynthetic process | 1/9 | 17/29008 | 0.005262784 | 0.013705686 | 0.002415446 | Il6 | 1 | 2.2788 | 189.5948 |
| GO:0035728 | response to hepatocyte growth factor | 1/9 | 17/29008 | 0.005262784 | 0.013705686 | 0.002415446 | Il6 | 1 | 2.2788 | 189.5948 |
| GO:0045064 | T-helper 2 cell differentiation | 1/9 | 17/29008 | 0.005262784 | 0.013705686 | 0.002415446 | Il6 | 1 | 2.2788 | 189.5948 |
| GO:0048143 | astrocyte activation | 1/9 | 17/29008 | 0.005262784 | 0.013705686 | 0.002415446 | Il1b | 1 | 2.2788 | 189.5948 |
| GO:0070886 | positive regulation of calcineurin-NFAT signaling cascade | 1/9 | 17/29008 | 0.005262784 | 0.013705686 | 0.002415446 | Tnf | 1 | 2.2788 | 189.5948 |
| GO:0071801 | regulation of podosome assembly | 1/9 | 17/29008 | 0.005262784 | 0.013705686 | 0.002415446 | Tnf | 1 | 2.2788 | 189.5948 |
| GO:0106058 | positive regulation of calcineurin-mediated signaling | 1/9 | 17/29008 | 0.005262784 | 0.013705686 | 0.002415446 | Tnf | 1 | 2.2788 | 189.5948 |
| GO:0018209 | peptidyl-serine modification | 2/9 | 365/29008 | 0.005361486 | 0.013929723 | 0.002454929 | Tnf/Il6 | 2 | 2.2707 | 17.6609 |
| GO:0002827 | positive regulation of T-helper 1 type immune response | 1/9 | 18/29008 | 0.005571591 | 0.014306492 | 0.00252133 | Il1b | 1 | 2.2540 | 179.0617 |
| GO:0002830 | positive regulation of type 2 immune response | 1/9 | 18/29008 | 0.005571591 | 0.014306492 | 0.00252133 | Il6 | 1 | 2.2540 | 179.0617 |
| GO:0070102 | interleukin-6-mediated signaling pathway | 1/9 | 18/29008 | 0.005571591 | 0.014306492 | 0.00252133 | Il6 | 1 | 2.2540 | 179.0617 |
| GO:0070486 | leukocyte aggregation | 1/9 | 18/29008 | 0.005571591 | 0.014306492 | 0.00252133 | Il1b | 1 | 2.2540 | 179.0617 |
| GO:1900221 | regulation of amyloid-beta clearance | 1/9 | 18/29008 | 0.005571591 | 0.014306492 | 0.00252133 | Tnf | 1 | 2.2540 | 179.0617 |
| GO:0050900 | leukocyte migration | 2/9 | 373/29008 | 0.005592198 | 0.014325934 | 0.002524756 | Tnf/Il1b | 2 | 2.2524 | 17.2821 |
| GO:0051346 | negative regulation of hydrolase activity | 2/9 | 374/29008 | 0.005621357 | 0.014367144 | 0.002532019 | Tnf/Il6 | 2 | 2.2502 | 17.2359 |
| GO:0010623 | programmed cell death involved in cell development | 1/9 | 19/29008 | 0.005880313 | 0.014771218 | 0.002603232 | Il1b | 1 | 2.2306 | 169.6374 |
| GO:0045721 | negative regulation of gluconeogenesis | 1/9 | 19/29008 | 0.005880313 | 0.014771218 | 0.002603232 | Il6 | 1 | 2.2306 | 169.6374 |
| GO:0045779 | negative regulation of bone resorption | 1/9 | 19/29008 | 0.005880313 | 0.014771218 | 0.002603232 | Il6 | 1 | 2.2306 | 169.6374 |
| GO:0045837 | negative regulation of membrane potential | 1/9 | 19/29008 | 0.005880313 | 0.014771218 | 0.002603232 | Il6 | 1 | 2.2306 | 169.6374 |
| GO:0051956 | negative regulation of amino acid transport | 1/9 | 19/29008 | 0.005880313 | 0.014771218 | 0.002603232 | Il1b | 1 | 2.2306 | 169.6374 |
| GO:1902004 | positive regulation of amyloid-beta formation | 1/9 | 19/29008 | 0.005880313 | 0.014771218 | 0.002603232 | Tnf | 1 | 2.2306 | 169.6374 |
| GO:1904385 | cellular response to angiotensin | 1/9 | 19/29008 | 0.005880313 | 0.014771218 | 0.002603232 | Nfkb1 | 1 | 2.2306 | 169.6374 |
| GO:0006909 | phagocytosis | 2/9 | 383/29008 | 0.005886982 | 0.014771218 | 0.002603232 | Tnf/Il1b | 2 | 2.2301 | 16.8309 |
| GO:0042176 | regulation of protein catabolic process | 2/9 | 389/29008 | 0.006067248 | 0.015047912 | 0.002651995 | Tnf/Il1b | 2 | 2.2170 | 16.5713 |
| GO:0006417 | regulation of translation | 2/9 | 390/29008 | 0.00609754 | 0.015047912 | 0.002651995 | Tnf/Il6 | 2 | 2.2148 | 16.5288 |
| GO:0002544 | chronic inflammatory response | 1/9 | 20/29008 | 0.00618895 | 0.015047912 | 0.002651995 | Tnf | 1 | 2.2084 | 161.1556 |
| GO:0002863 | positive regulation of inflammatory response to antigenic stimulus | 1/9 | 20/29008 | 0.00618895 | 0.015047912 | 0.002651995 | Tnf | 1 | 2.2084 | 161.1556 |
| GO:0002923 | regulation of humoral immune response mediated by circulating immunoglobulin | 1/9 | 20/29008 | 0.00618895 | 0.015047912 | 0.002651995 | Tnf | 1 | 2.2084 | 161.1556 |
| GO:0006309 | apoptotic DNA fragmentation | 1/9 | 20/29008 | 0.00618895 | 0.015047912 | 0.002651995 | Il6 | 1 | 2.2084 | 161.1556 |
| GO:0006390 | mitochondrial transcription | 1/9 | 20/29008 | 0.00618895 | 0.015047912 | 0.002651995 | Tfam | 1 | 2.2084 | 161.1556 |
| GO:0032305 | positive regulation of icosanoid secretion | 1/9 | 20/29008 | 0.00618895 | 0.015047912 | 0.002651995 | Il1b | 1 | 2.2084 | 161.1556 |
| GO:0032310 | prostaglandin secretion | 1/9 | 20/29008 | 0.00618895 | 0.015047912 | 0.002651995 | Il1b | 1 | 2.2084 | 161.1556 |
| GO:0032695 | negative regulation of interleukin-12 production | 1/9 | 20/29008 | 0.00618895 | 0.015047912 | 0.002651995 | Nfkb1 | 1 | 2.2084 | 161.1556 |
| GO:0043373 | CD4-positive, alpha-beta T cell lineage commitment | 1/9 | 20/29008 | 0.00618895 | 0.015047912 | 0.002651995 | Il6 | 1 | 2.2084 | 161.1556 |
| GO:0045948 | positive regulation of translational initiation | 1/9 | 20/29008 | 0.00618895 | 0.015047912 | 0.002651995 | Tnf | 1 | 2.2084 | 161.1556 |
| GO:0048635 | negative regulation of muscle organ development | 1/9 | 20/29008 | 0.00618895 | 0.015047912 | 0.002651995 | Il6 | 1 | 2.2084 | 161.1556 |
| GO:0071639 | positive regulation of monocyte chemotactic protein-1 production | 1/9 | 20/29008 | 0.00618895 | 0.015047912 | 0.002651995 | Il1b | 1 | 2.2084 | 161.1556 |
| GO:0042326 | negative regulation of phosphorylation | 2/9 | 396/29008 | 0.006280765 | 0.015237442 | 0.002685397 | Il6/Il1b | 2 | 2.2020 | 16.2783 |
| GO:0032102 | negative regulation of response to external stimulus | 2/9 | 398/29008 | 0.006342403 | 0.015319342 | 0.002699831 | Tnf/Nfkb1 | 2 | 2.1977 | 16.1965 |
| GO:1903706 | regulation of hemopoiesis | 2/9 | 398/29008 | 0.006342403 | 0.015319342 | 0.002699831 | Tnf/Il6 | 2 | 2.1977 | 16.1965 |
| GO:0022411 | cellular component disassembly | 2/9 | 402/29008 | 0.006466519 | 0.015390081 | 0.002712298 | Tnf/Il6 | 2 | 2.1893 | 16.0354 |
| GO:2001233 | regulation of apoptotic signaling pathway | 2/9 | 402/29008 | 0.006466519 | 0.015390081 | 0.002712298 | Tnf/Il1b | 2 | 2.1893 | 16.0354 |
| GO:0007252 | I-kappaB phosphorylation | 1/9 | 21/29008 | 0.006497502 | 0.015390081 | 0.002712298 | Tnf | 1 | 2.1873 | 153.4815 |
| GO:0010666 | positive regulation of cardiac muscle cell apoptotic process | 1/9 | 21/29008 | 0.006497502 | 0.015390081 | 0.002712298 | Fbxo32 | 1 | 2.1873 | 153.4815 |
| GO:0035994 | response to muscle stretch | 1/9 | 21/29008 | 0.006497502 | 0.015390081 | 0.002712298 | Nfkb1 | 1 | 2.1873 | 153.4815 |
| GO:0046851 | negative regulation of bone remodeling | 1/9 | 21/29008 | 0.006497502 | 0.015390081 | 0.002712298 | Il6 | 1 | 2.1873 | 153.4815 |
| GO:0051797 | regulation of hair follicle development | 1/9 | 21/29008 | 0.006497502 | 0.015390081 | 0.002712298 | Tnf | 1 | 2.1873 | 153.4815 |
| GO:1990776 | response to angiotensin | 1/9 | 21/29008 | 0.006497502 | 0.015390081 | 0.002712298 | Nfkb1 | 1 | 2.1873 | 153.4815 |
| GO:0050678 | regulation of epithelial cell proliferation | 2/9 | 403/29008 | 0.006497723 | 0.015390081 | 0.002712298 | Tnf/Il6 | 2 | 2.1872 | 15.9956 |
| GO:0006979 | response to oxidative stress | 2/9 | 407/29008 | 0.006623239 | 0.015653633 | 0.002758746 | Il6/Nfkb1 | 2 | 2.1789 | 15.8384 |
| GO:1901652 | response to peptide | 2/9 | 412/29008 | 0.006781704 | 0.015780084 | 0.002781031 | Il1b/Nfkb1 | 2 | 2.1687 | 15.6462 |
| GO:0002363 | alpha-beta T cell lineage commitment | 1/9 | 22/29008 | 0.006805969 | 0.015780084 | 0.002781031 | Il6 | 1 | 2.1671 | 146.5051 |
| GO:0010663 | positive regulation of striated muscle cell apoptotic process | 1/9 | 22/29008 | 0.006805969 | 0.015780084 | 0.002781031 | Fbxo32 | 1 | 2.1671 | 146.5051 |
| GO:0045624 | positive regulation of T-helper cell differentiation | 1/9 | 22/29008 | 0.006805969 | 0.015780084 | 0.002781031 | Il6 | 1 | 2.1671 | 146.5051 |
| GO:0046325 | negative regulation of glucose import | 1/9 | 22/29008 | 0.006805969 | 0.015780084 | 0.002781031 | Tnf | 1 | 2.1671 | 146.5051 |
| GO:0051602 | response to electrical stimulus | 1/9 | 22/29008 | 0.006805969 | 0.015780084 | 0.002781031 | Fbxo32 | 1 | 2.1671 | 146.5051 |
| GO:0060055 | angiogenesis involved in wound healing | 1/9 | 22/29008 | 0.006805969 | 0.015780084 | 0.002781031 | Tnf | 1 | 2.1671 | 146.5051 |
| GO:0071800 | podosome assembly | 1/9 | 22/29008 | 0.006805969 | 0.015780084 | 0.002781031 | Tnf | 1 | 2.1671 | 146.5051 |
| GO:2000810 | regulation of bicellular tight junction assembly | 1/9 | 22/29008 | 0.006805969 | 0.015780084 | 0.002781031 | Tnf | 1 | 2.1671 | 146.5051 |
| GO:0006959 | humoral immune response | 2/9 | 416/29008 | 0.006909731 | 0.015986935 | 0.002817486 | Tnf/Il1b | 2 | 2.1605 | 15.4957 |
| GO:0001503 | ossification | 2/9 | 417/29008 | 0.006941911 | 0.016027647 | 0.002824661 | Tnf/Il6 | 2 | 2.1585 | 15.4586 |
| GO:0032740 | positive regulation of interleukin-17 production | 1/9 | 23/29008 | 0.00711435 | 0.016288898 | 0.002870703 | Il6 | 1 | 2.1479 | 140.1353 |
| GO:0046716 | muscle cell cellular homeostasis | 1/9 | 23/29008 | 0.00711435 | 0.016288898 | 0.002870703 | Il6 | 1 | 2.1479 | 140.1353 |
| GO:0051969 | regulation of transmission of nerve impulse | 1/9 | 23/29008 | 0.00711435 | 0.016288898 | 0.002870703 | Il6 | 1 | 2.1479 | 140.1353 |
| GO:0071359 | cellular response to dsRNA | 1/9 | 23/29008 | 0.00711435 | 0.016288898 | 0.002870703 | Nfkb1 | 1 | 2.1479 | 140.1353 |
| GO:0006091 | generation of precursor metabolites and energy | 2/9 | 428/29008 | 0.007300472 | 0.016680287 | 0.00293968 | Ndufb8/Sdhb | 2 | 2.1366 | 15.0613 |
| GO:0015732 | prostaglandin transport | 1/9 | 24/29008 | 0.007422647 | 0.016784956 | 0.002958126 | Il1b | 1 | 2.1294 | 134.2963 |
| GO:0030449 | regulation of complement activation | 1/9 | 24/29008 | 0.007422647 | 0.016784956 | 0.002958126 | Il1b | 1 | 2.1294 | 134.2963 |
| GO:0032515 | negative regulation of phosphoprotein phosphatase activity | 1/9 | 24/29008 | 0.007422647 | 0.016784956 | 0.002958126 | Tnf | 1 | 2.1294 | 134.2963 |
| GO:0043369 | CD4-positive or CD8-positive, alpha-beta T cell lineage commitment | 1/9 | 24/29008 | 0.007422647 | 0.016784956 | 0.002958126 | Il6 | 1 | 2.1294 | 134.2963 |
| GO:2000010 | positive regulation of protein localization to cell surface | 1/9 | 24/29008 | 0.007422647 | 0.016784956 | 0.002958126 | Tnf | 1 | 2.1294 | 134.2963 |
| GO:0033674 | positive regulation of kinase activity | 2/9 | 435/29008 | 0.007533 | 0.016999522 | 0.002995941 | Tnf/Il1b | 2 | 2.1230 | 14.8189 |
| GO:0051251 | positive regulation of lymphocyte activation | 2/9 | 436/29008 | 0.007566494 | 0.017040117 | 0.003003095 | Il6/Il1b | 2 | 2.1211 | 14.7849 |
| GO:0010876 | lipid localization | 2/9 | 437/29008 | 0.007600057 | 0.017069798 | 0.003008326 | Tnf/Il1b | 2 | 2.1192 | 14.7511 |
| GO:0034329 | cell junction assembly | 2/9 | 439/29008 | 0.007667388 | 0.017069798 | 0.003008326 | Tnf/Il1b | 2 | 2.1154 | 14.6839 |
| GO:0002719 | negative regulation of cytokine production involved in immune response | 1/9 | 25/29008 | 0.007730858 | 0.017069798 | 0.003008326 | Tnf | 1 | 2.1118 | 128.9244 |
| GO:0010894 | negative regulation of steroid biosynthetic process | 1/9 | 25/29008 | 0.007730858 | 0.017069798 | 0.003008326 | Nfkb1 | 1 | 2.1118 | 128.9244 |
| GO:0014048 | regulation of glutamate secretion | 1/9 | 25/29008 | 0.007730858 | 0.017069798 | 0.003008326 | Il1b | 1 | 2.1118 | 128.9244 |
| GO:0032303 | regulation of icosanoid secretion | 1/9 | 25/29008 | 0.007730858 | 0.017069798 | 0.003008326 | Il1b | 1 | 2.1118 | 128.9244 |
| GO:1902993 | positive regulation of amyloid precursor protein catabolic process | 1/9 | 25/29008 | 0.007730858 | 0.017069798 | 0.003008326 | Tnf | 1 | 2.1118 | 128.9244 |
| GO:2000193 | positive regulation of fatty acid transport | 1/9 | 25/29008 | 0.007730858 | 0.017069798 | 0.003008326 | Il1b | 1 | 2.1118 | 128.9244 |
| GO:0030098 | lymphocyte differentiation | 2/9 | 441/29008 | 0.007734995 | 0.017069798 | 0.003008326 | Il6/Il1b | 2 | 2.1115 | 14.6173 |
| GO:0052547 | regulation of peptidase activity | 2/9 | 441/29008 | 0.007734995 | 0.017069798 | 0.003008326 | Tnf/Il6 | 2 | 2.1115 | 14.6173 |
| GO:0034248 | regulation of cellular amide metabolic process | 2/9 | 448/29008 | 0.007973775 | 0.017425727 | 0.003071054 | Tnf/Il6 | 2 | 2.0983 | 14.3889 |
| GO:0006925 | inflammatory cell apoptotic process | 1/9 | 26/29008 | 0.008038984 | 0.017425727 | 0.003071054 | Il6 | 1 | 2.0948 | 123.9658 |
| GO:0045939 | negative regulation of steroid metabolic process | 1/9 | 26/29008 | 0.008038984 | 0.017425727 | 0.003071054 | Nfkb1 | 1 | 2.0948 | 123.9658 |
| GO:0051354 | negative regulation of oxidoreductase activity | 1/9 | 26/29008 | 0.008038984 | 0.017425727 | 0.003071054 | Nfkb1 | 1 | 2.0948 | 123.9658 |
| GO:0071549 | cellular response to dexamethasone stimulus | 1/9 | 26/29008 | 0.008038984 | 0.017425727 | 0.003071054 | Fbxo32 | 1 | 2.0948 | 123.9658 |
| GO:0072567 | chemokine (C-X-C motif) ligand 2 production | 1/9 | 26/29008 | 0.008038984 | 0.017425727 | 0.003071054 | Tnf | 1 | 2.0948 | 123.9658 |
| GO:0090330 | regulation of platelet aggregation | 1/9 | 26/29008 | 0.008038984 | 0.017425727 | 0.003071054 | Il6 | 1 | 2.0948 | 123.9658 |
| GO:1903055 | positive regulation of extracellular matrix organization | 1/9 | 26/29008 | 0.008038984 | 0.017425727 | 0.003071054 | Il6 | 1 | 2.0948 | 123.9658 |
| GO:2000341 | regulation of chemokine (C-X-C motif) ligand 2 production | 1/9 | 26/29008 | 0.008038984 | 0.017425727 | 0.003071054 | Tnf | 1 | 2.0948 | 123.9658 |
| GO:0034660 | ncRNA metabolic process | 2/9 | 453/29008 | 0.008146384 | 0.017623771 | 0.003105957 | Il6/Nfkb1 | 2 | 2.0890 | 14.2301 |
| GO:0001780 | neutrophil homeostasis | 1/9 | 27/29008 | 0.008347025 | 0.01791676 | 0.003157592 | Il6 | 1 | 2.0785 | 119.3745 |
| GO:0031100 | animal organ regeneration | 1/9 | 27/29008 | 0.008347025 | 0.01791676 | 0.003157592 | Il6 | 1 | 2.0785 | 119.3745 |
| GO:0033198 | response to ATP | 1/9 | 27/29008 | 0.008347025 | 0.01791676 | 0.003157592 | Il1b | 1 | 2.0785 | 119.3745 |
| GO:0045723 | positive regulation of fatty acid biosynthetic process | 1/9 | 27/29008 | 0.008347025 | 0.01791676 | 0.003157592 | Il1b | 1 | 2.0785 | 119.3745 |
| GO:0007346 | regulation of mitotic cell cycle | 2/9 | 462/29008 | 0.00846137 | 0.018126795 | 0.003194608 | Tnf/Il1b | 2 | 2.0726 | 13.9529 |
| GO:0000737 | DNA catabolic process, endonucleolytic | 1/9 | 28/29008 | 0.008654982 | 0.018221886 | 0.003211366 | Il6 | 1 | 2.0627 | 115.1111 |
| GO:0010893 | positive regulation of steroid biosynthetic process | 1/9 | 28/29008 | 0.008654982 | 0.018221886 | 0.003211366 | Tnf | 1 | 2.0627 | 115.1111 |
| GO:0034104 | negative regulation of tissue remodeling | 1/9 | 28/29008 | 0.008654982 | 0.018221886 | 0.003211366 | Il6 | 1 | 2.0627 | 115.1111 |
| GO:0045187 | regulation of circadian sleep/wake cycle, sleep | 1/9 | 28/29008 | 0.008654982 | 0.018221886 | 0.003211366 | Il6 | 1 | 2.0627 | 115.1111 |
| GO:0071605 | monocyte chemotactic protein-1 production | 1/9 | 28/29008 | 0.008654982 | 0.018221886 | 0.003211366 | Il1b | 1 | 2.0627 | 115.1111 |
| GO:0071637 | regulation of monocyte chemotactic protein-1 production | 1/9 | 28/29008 | 0.008654982 | 0.018221886 | 0.003211366 | Il1b | 1 | 2.0627 | 115.1111 |
| GO:0090023 | positive regulation of neutrophil chemotaxis | 1/9 | 28/29008 | 0.008654982 | 0.018221886 | 0.003211366 | Il1b | 1 | 2.0627 | 115.1111 |
| GO:1900745 | positive regulation of p38MAPK cascade | 1/9 | 28/29008 | 0.008654982 | 0.018221886 | 0.003211366 | Il1b | 1 | 2.0627 | 115.1111 |
| GO:2000178 | negative regulation of neural precursor cell proliferation | 1/9 | 28/29008 | 0.008654982 | 0.018221886 | 0.003211366 | Il1b | 1 | 2.0627 | 115.1111 |
| GO:0050673 | epithelial cell proliferation | 2/9 | 471/29008 | 0.008781852 | 0.018453643 | 0.00325221 | Tnf/Il6 | 2 | 2.0564 | 13.6862 |
| GO:0002313 | mature B cell differentiation involved in immune response | 1/9 | 29/29008 | 0.008962853 | 0.018691034 | 0.003294048 | Il6 | 1 | 2.0476 | 111.1418 |
| GO:0006120 | mitochondrial electron transport, NADH to ubiquinone | 1/9 | 29/29008 | 0.008962853 | 0.018691034 | 0.003294048 | Ndufb8 | 1 | 2.0476 | 111.1418 |
| GO:0032891 | negative regulation of organic acid transport | 1/9 | 29/29008 | 0.008962853 | 0.018691034 | 0.003294048 | Il1b | 1 | 2.0476 | 111.1418 |
| GO:0071548 | response to dexamethasone | 1/9 | 29/29008 | 0.008962853 | 0.018691034 | 0.003294048 | Fbxo32 | 1 | 2.0476 | 111.1418 |
| GO:0048732 | gland development | 2/9 | 477/29008 | 0.008998546 | 0.018729929 | 0.003300902 | Tnf/Il6 | 2 | 2.0458 | 13.5141 |
| GO:0001516 | prostaglandin biosynthetic process | 1/9 | 30/29008 | 0.009270639 | 0.018972872 | 0.003343718 | Il1b | 1 | 2.0329 | 107.4370 |
| GO:0010586 | miRNA metabolic process | 1/9 | 30/29008 | 0.009270639 | 0.018972872 | 0.003343718 | Nfkb1 | 1 | 2.0329 | 107.4370 |
| GO:0030262 | apoptotic nuclear changes | 1/9 | 30/29008 | 0.009270639 | 0.018972872 | 0.003343718 | Il6 | 1 | 2.0329 | 107.4370 |
| GO:0032682 | negative regulation of chemokine production | 1/9 | 30/29008 | 0.009270639 | 0.018972872 | 0.003343718 | Il6 | 1 | 2.0329 | 107.4370 |
| GO:0045454 | cell redox homeostasis | 1/9 | 30/29008 | 0.009270639 | 0.018972872 | 0.003343718 | Il6 | 1 | 2.0329 | 107.4370 |
| GO:0046457 | prostanoid biosynthetic process | 1/9 | 30/29008 | 0.009270639 | 0.018972872 | 0.003343718 | Il1b | 1 | 2.0329 | 107.4370 |
| GO:0046885 | regulation of hormone biosynthetic process | 1/9 | 30/29008 | 0.009270639 | 0.018972872 | 0.003343718 | Nfkb1 | 1 | 2.0329 | 107.4370 |
| GO:0050802 | circadian sleep/wake cycle, sleep | 1/9 | 30/29008 | 0.009270639 | 0.018972872 | 0.003343718 | Il6 | 1 | 2.0329 | 107.4370 |
| GO:0071624 | positive regulation of granulocyte chemotaxis | 1/9 | 30/29008 | 0.009270639 | 0.018972872 | 0.003343718 | Il1b | 1 | 2.0329 | 107.4370 |
| GO:1903131 | mononuclear cell differentiation | 2/9 | 489/29008 | 0.009439197 | 0.019244233 | 0.003391542 | Il6/Il1b | 2 | 2.0251 | 13.1825 |
| GO:0002360 | T cell lineage commitment | 1/9 | 31/29008 | 0.00957834 | 0.019244233 | 0.003391542 | Il6 | 1 | 2.0187 | 103.9713 |
| GO:0002825 | regulation of T-helper 1 type immune response | 1/9 | 31/29008 | 0.00957834 | 0.019244233 | 0.003391542 | Il1b | 1 | 2.0187 | 103.9713 |
| GO:0006921 | cellular component disassembly involved in execution phase of apoptosis | 1/9 | 31/29008 | 0.00957834 | 0.019244233 | 0.003391542 | Il6 | 1 | 2.0187 | 103.9713 |
| GO:0042634 | regulation of hair cycle | 1/9 | 31/29008 | 0.00957834 | 0.019244233 | 0.003391542 | Tnf | 1 | 2.0187 | 103.9713 |
| GO:0042749 | regulation of circadian sleep/wake cycle | 1/9 | 31/29008 | 0.00957834 | 0.019244233 | 0.003391542 | Il6 | 1 | 2.0187 | 103.9713 |
| GO:0045662 | negative regulation of myoblast differentiation | 1/9 | 31/29008 | 0.00957834 | 0.019244233 | 0.003391542 | Tnf | 1 | 2.0187 | 103.9713 |
| GO:0072539 | T-helper 17 cell differentiation | 1/9 | 31/29008 | 0.00957834 | 0.019244233 | 0.003391542 | Il6 | 1 | 2.0187 | 103.9713 |
| GO:0072574 | hepatocyte proliferation | 1/9 | 31/29008 | 0.00957834 | 0.019244233 | 0.003391542 | Il6 | 1 | 2.0187 | 103.9713 |
| GO:0072575 | epithelial cell proliferation involved in liver morphogenesis | 1/9 | 31/29008 | 0.00957834 | 0.019244233 | 0.003391542 | Il6 | 1 | 2.0187 | 103.9713 |
| GO:0002696 | positive regulation of leukocyte activation | 2/9 | 500/29008 | 0.009851588 | 0.019718087 | 0.003475052 | Il6/Il1b | 2 | 2.0065 | 12.8924 |
| GO:0006099 | tricarboxylic acid cycle | 1/9 | 32/29008 | 0.009885956 | 0.019718087 | 0.003475052 | Sdhb | 1 | 2.0050 | 100.7222 |
| GO:0043372 | positive regulation of CD4-positive, alpha-beta T cell differentiation | 1/9 | 32/29008 | 0.009885956 | 0.019718087 | 0.003475052 | Il6 | 1 | 2.0050 | 100.7222 |
| GO:0048535 | lymph node development | 1/9 | 32/29008 | 0.009885956 | 0.019718087 | 0.003475052 | Nfkb1 | 1 | 2.0050 | 100.7222 |
| GO:0002828 | regulation of type 2 immune response | 1/9 | 33/29008 | 0.010193487 | 0.020076421 | 0.003538204 | Il6 | 1 | 1.9917 | 97.6700 |
| GO:0010614 | negative regulation of cardiac muscle hypertrophy | 1/9 | 33/29008 | 0.010193487 | 0.020076421 | 0.003538204 | Fbxo32 | 1 | 1.9917 | 97.6700 |
| GO:0022410 | circadian sleep/wake cycle process | 1/9 | 33/29008 | 0.010193487 | 0.020076421 | 0.003538204 | Il6 | 1 | 1.9917 | 97.6700 |
| GO:0050996 | positive regulation of lipid catabolic process | 1/9 | 33/29008 | 0.010193487 | 0.020076421 | 0.003538204 | Il1b | 1 | 1.9917 | 97.6700 |
| GO:0070266 | necroptotic process | 1/9 | 33/29008 | 0.010193487 | 0.020076421 | 0.003538204 | Tnf | 1 | 1.9917 | 97.6700 |
| GO:0072576 | liver morphogenesis | 1/9 | 33/29008 | 0.010193487 | 0.020076421 | 0.003538204 | Il6 | 1 | 1.9917 | 97.6700 |
| GO:1903792 | negative regulation of anion transport | 1/9 | 33/29008 | 0.010193487 | 0.020076421 | 0.003538204 | Il1b | 1 | 1.9917 | 97.6700 |
| GO:0006308 | DNA catabolic process | 1/9 | 34/29008 | 0.010500934 | 0.02049827 | 0.003612549 | Il6 | 1 | 1.9788 | 94.7974 |
| GO:0014047 | glutamate secretion | 1/9 | 34/29008 | 0.010500934 | 0.02049827 | 0.003612549 | Il1b | 1 | 1.9788 | 94.7974 |
| GO:0035308 | negative regulation of protein dephosphorylation | 1/9 | 34/29008 | 0.010500934 | 0.02049827 | 0.003612549 | Tnf | 1 | 1.9788 | 94.7974 |
| GO:1904707 | positive regulation of vascular associated smooth muscle cell proliferation | 1/9 | 34/29008 | 0.010500934 | 0.02049827 | 0.003612549 | Tnf | 1 | 1.9788 | 94.7974 |
| GO:2000272 | negative regulation of signaling receptor activity | 1/9 | 34/29008 | 0.010500934 | 0.02049827 | 0.003612549 | Tnf | 1 | 1.9788 | 94.7974 |
| GO:0014002 | astrocyte development | 1/9 | 35/29008 | 0.010808295 | 0.020730046 | 0.003653396 | Il1b | 1 | 1.9662 | 92.0889 |
| GO:0014741 | negative regulation of muscle hypertrophy | 1/9 | 35/29008 | 0.010808295 | 0.020730046 | 0.003653396 | Fbxo32 | 1 | 1.9662 | 92.0889 |
| GO:0043243 | positive regulation of protein-containing complex disassembly | 1/9 | 35/29008 | 0.010808295 | 0.020730046 | 0.003653396 | Tnf | 1 | 1.9662 | 92.0889 |
| GO:0043537 | negative regulation of blood vessel endothelial cell migration | 1/9 | 35/29008 | 0.010808295 | 0.020730046 | 0.003653396 | Tnf | 1 | 1.9662 | 92.0889 |
| GO:0045672 | positive regulation of osteoclast differentiation | 1/9 | 35/29008 | 0.010808295 | 0.020730046 | 0.003653396 | Tnf | 1 | 1.9662 | 92.0889 |
| GO:0048710 | regulation of astrocyte differentiation | 1/9 | 35/29008 | 0.010808295 | 0.020730046 | 0.003653396 | Il1b | 1 | 1.9662 | 92.0889 |
| GO:0050999 | regulation of nitric-oxide synthase activity | 1/9 | 35/29008 | 0.010808295 | 0.020730046 | 0.003653396 | Il1b | 1 | 1.9662 | 92.0889 |
| GO:0090022 | regulation of neutrophil chemotaxis | 1/9 | 35/29008 | 0.010808295 | 0.020730046 | 0.003653396 | Il1b | 1 | 1.9662 | 92.0889 |
| GO:1990089 | response to nerve growth factor | 1/9 | 35/29008 | 0.010808295 | 0.020730046 | 0.003653396 | Nfkb1 | 1 | 1.9662 | 92.0889 |
| GO:1990090 | cellular response to nerve growth factor stimulus | 1/9 | 35/29008 | 0.010808295 | 0.020730046 | 0.003653396 | Nfkb1 | 1 | 1.9662 | 92.0889 |
| GO:0022617 | extracellular matrix disassembly | 1/9 | 36/29008 | 0.011115572 | 0.021134971 | 0.003724759 | Il6 | 1 | 1.9541 | 89.5309 |
| GO:0042832 | defense response to protozoan | 1/9 | 36/29008 | 0.011115572 | 0.021134971 | 0.003724759 | Il6 | 1 | 1.9541 | 89.5309 |
| GO:0050901 | leukocyte tethering or rolling | 1/9 | 36/29008 | 0.011115572 | 0.021134971 | 0.003724759 | Tnf | 1 | 1.9541 | 89.5309 |
| GO:0060441 | epithelial tube branching involved in lung morphogenesis | 1/9 | 36/29008 | 0.011115572 | 0.021134971 | 0.003724759 | Tnf | 1 | 1.9541 | 89.5309 |
| GO:0097242 | amyloid-beta clearance | 1/9 | 36/29008 | 0.011115572 | 0.021134971 | 0.003724759 | Tnf | 1 | 1.9541 | 89.5309 |
| GO:0002335 | mature B cell differentiation | 1/9 | 37/29008 | 0.011422764 | 0.021459175 | 0.003781896 | Il6 | 1 | 1.9422 | 87.1111 |
| GO:0010661 | positive regulation of muscle cell apoptotic process | 1/9 | 37/29008 | 0.011422764 | 0.021459175 | 0.003781896 | Fbxo32 | 1 | 1.9422 | 87.1111 |
| GO:0010923 | negative regulation of phosphatase activity | 1/9 | 37/29008 | 0.011422764 | 0.021459175 | 0.003781896 | Tnf | 1 | 1.9422 | 87.1111 |
| GO:0034110 | regulation of homotypic cell-cell adhesion | 1/9 | 37/29008 | 0.011422764 | 0.021459175 | 0.003781896 | Il6 | 1 | 1.9422 | 87.1111 |
| GO:0051953 | negative regulation of amine transport | 1/9 | 37/29008 | 0.011422764 | 0.021459175 | 0.003781896 | Il1b | 1 | 1.9422 | 87.1111 |
| GO:1902624 | positive regulation of neutrophil migration | 1/9 | 37/29008 | 0.011422764 | 0.021459175 | 0.003781896 | Il1b | 1 | 1.9422 | 87.1111 |
| GO:2000191 | regulation of fatty acid transport | 1/9 | 37/29008 | 0.011422764 | 0.021459175 | 0.003781896 | Il1b | 1 | 1.9422 | 87.1111 |
| GO:0002701 | negative regulation of production of molecular mediator of immune response | 1/9 | 38/29008 | 0.011729871 | 0.021849369 | 0.003850662 | Tnf | 1 | 1.9307 | 84.8187 |
| GO:0030866 | cortical actin cytoskeleton organization | 1/9 | 38/29008 | 0.011729871 | 0.021849369 | 0.003850662 | Tnf | 1 | 1.9307 | 84.8187 |
| GO:0033028 | myeloid cell apoptotic process | 1/9 | 38/29008 | 0.011729871 | 0.021849369 | 0.003850662 | Il6 | 1 | 1.9307 | 84.8187 |
| GO:0045622 | regulation of T-helper cell differentiation | 1/9 | 38/29008 | 0.011729871 | 0.021849369 | 0.003850662 | Il6 | 1 | 1.9307 | 84.8187 |
| GO:1902003 | regulation of amyloid-beta formation | 1/9 | 38/29008 | 0.011729871 | 0.021849369 | 0.003850662 | Tnf | 1 | 1.9307 | 84.8187 |
| GO:0002861 | regulation of inflammatory response to antigenic stimulus | 1/9 | 39/29008 | 0.012036893 | 0.022195545 | 0.003911671 | Tnf | 1 | 1.9195 | 82.6439 |
| GO:0014823 | response to activity | 1/9 | 39/29008 | 0.012036893 | 0.022195545 | 0.003911671 | Il6 | 1 | 1.9195 | 82.6439 |
| GO:0032743 | positive regulation of interleukin-2 production | 1/9 | 39/29008 | 0.012036893 | 0.022195545 | 0.003911671 | Il1b | 1 | 1.9195 | 82.6439 |
| GO:0032885 | regulation of polysaccharide biosynthetic process | 1/9 | 39/29008 | 0.012036893 | 0.022195545 | 0.003911671 | Nfkb1 | 1 | 1.9195 | 82.6439 |
| GO:0042745 | circadian sleep/wake cycle | 1/9 | 39/29008 | 0.012036893 | 0.022195545 | 0.003911671 | Il6 | 1 | 1.9195 | 82.6439 |
| GO:0070884 | regulation of calcineurin-NFAT signaling cascade | 1/9 | 39/29008 | 0.012036893 | 0.022195545 | 0.003911671 | Tnf | 1 | 1.9195 | 82.6439 |
| GO:0001562 | response to protozoan | 1/9 | 40/29008 | 0.01234383 | 0.022497296 | 0.003964851 | Il6 | 1 | 1.9086 | 80.5778 |
| GO:0010543 | regulation of platelet activation | 1/9 | 40/29008 | 0.01234383 | 0.022497296 | 0.003964851 | Il6 | 1 | 1.9086 | 80.5778 |
| GO:0030431 | sleep | 1/9 | 40/29008 | 0.01234383 | 0.022497296 | 0.003964851 | Il6 | 1 | 1.9086 | 80.5778 |
| GO:0032660 | regulation of interleukin-17 production | 1/9 | 40/29008 | 0.01234383 | 0.022497296 | 0.003964851 | Il6 | 1 | 1.9086 | 80.5778 |
| GO:0050850 | positive regulation of calcium-mediated signaling | 1/9 | 40/29008 | 0.01234383 | 0.022497296 | 0.003964851 | Tnf | 1 | 1.9086 | 80.5778 |
| GO:0106056 | regulation of calcineurin-mediated signaling | 1/9 | 40/29008 | 0.01234383 | 0.022497296 | 0.003964851 | Tnf | 1 | 1.9086 | 80.5778 |
| GO:2000516 | positive regulation of CD4-positive, alpha-beta T cell activation | 1/9 | 40/29008 | 0.01234383 | 0.022497296 | 0.003964851 | Il6 | 1 | 1.9086 | 80.5778 |
| GO:0006636 | unsaturated fatty acid biosynthetic process | 1/9 | 41/29008 | 0.012650683 | 0.022866941 | 0.004029996 | Il1b | 1 | 1.8979 | 78.6125 |
| GO:0032733 | positive regulation of interleukin-10 production | 1/9 | 41/29008 | 0.012650683 | 0.022866941 | 0.004029996 | Il6 | 1 | 1.8979 | 78.6125 |
| GO:0042092 | type 2 immune response | 1/9 | 41/29008 | 0.012650683 | 0.022866941 | 0.004029996 | Il6 | 1 | 1.8979 | 78.6125 |
| GO:0043368 | positive T cell selection | 1/9 | 41/29008 | 0.012650683 | 0.022866941 | 0.004029996 | Il6 | 1 | 1.8979 | 78.6125 |
| GO:0046627 | negative regulation of insulin receptor signaling pathway | 1/9 | 41/29008 | 0.012650683 | 0.022866941 | 0.004029996 | Il1b | 1 | 1.8979 | 78.6125 |
| GO:0001774 | microglial cell activation | 1/9 | 42/29008 | 0.01295745 | 0.023306445 | 0.004107453 | Tnf | 1 | 1.8875 | 76.7407 |
| GO:0072538 | T-helper 17 type immune response | 1/9 | 42/29008 | 0.01295745 | 0.023306445 | 0.004107453 | Il6 | 1 | 1.8875 | 76.7407 |
| GO:1900744 | regulation of p38MAPK cascade | 1/9 | 42/29008 | 0.01295745 | 0.023306445 | 0.004107453 | Il1b | 1 | 1.8875 | 76.7407 |
| GO:0097300 | programmed necrotic cell death | 1/9 | 43/29008 | 0.013264134 | 0.023780233 | 0.004190951 | Tnf | 1 | 1.8773 | 74.9561 |
| GO:2000008 | regulation of protein localization to cell surface | 1/9 | 43/29008 | 0.013264134 | 0.023780233 | 0.004190951 | Tnf | 1 | 1.8773 | 74.9561 |
| GO:0032620 | interleukin-17 production | 1/9 | 44/29008 | 0.013570732 | 0.024250788 | 0.004273881 | Il6 | 1 | 1.8674 | 73.2525 |
| GO:0045940 | positive regulation of steroid metabolic process | 1/9 | 44/29008 | 0.013570732 | 0.024250788 | 0.004273881 | Tnf | 1 | 1.8674 | 73.2525 |
| GO:0045840 | positive regulation of mitotic nuclear division | 1/9 | 45/29008 | 0.013877246 | 0.024638276 | 0.00434217 | Il1b | 1 | 1.8577 | 71.6247 |
| GO:0050691 | regulation of defense response to virus by host | 1/9 | 45/29008 | 0.013877246 | 0.024638276 | 0.00434217 | Il1b | 1 | 1.8577 | 71.6247 |
| GO:0071385 | cellular response to glucocorticoid stimulus | 1/9 | 45/29008 | 0.013877246 | 0.024638276 | 0.00434217 | Fbxo32 | 1 | 1.8577 | 71.6247 |
| GO:1902991 | regulation of amyloid precursor protein catabolic process | 1/9 | 45/29008 | 0.013877246 | 0.024638276 | 0.00434217 | Tnf | 1 | 1.8577 | 71.6247 |
| GO:0032881 | regulation of polysaccharide metabolic process | 1/9 | 46/29008 | 0.014183675 | 0.025060866 | 0.004416646 | Nfkb1 | 1 | 1.8482 | 70.0676 |
| GO:0032965 | regulation of collagen biosynthetic process | 1/9 | 46/29008 | 0.014183675 | 0.025060866 | 0.004416646 | Il6 | 1 | 1.8482 | 70.0676 |
| GO:1900077 | negative regulation of cellular response to insulin stimulus | 1/9 | 46/29008 | 0.014183675 | 0.025060866 | 0.004416646 | Il1b | 1 | 1.8482 | 70.0676 |
| GO:1903053 | regulation of extracellular matrix organization | 1/9 | 47/29008 | 0.014490019 | 0.025561045 | 0.004504796 | Il6 | 1 | 1.8389 | 68.5768 |
| GO:0000959 | mitochondrial RNA metabolic process | 1/9 | 48/29008 | 0.014796279 | 0.025893488 | 0.004563385 | Tfam | 1 | 1.8298 | 67.1481 |
| GO:0034205 | amyloid-beta formation | 1/9 | 48/29008 | 0.014796279 | 0.025893488 | 0.004563385 | Tnf | 1 | 1.8298 | 67.1481 |
| GO:0042304 | regulation of fatty acid biosynthetic process | 1/9 | 48/29008 | 0.014796279 | 0.025893488 | 0.004563385 | Il1b | 1 | 1.8298 | 67.1481 |
| GO:0045740 | positive regulation of DNA replication | 1/9 | 48/29008 | 0.014796279 | 0.025893488 | 0.004563385 | Il6 | 1 | 1.8298 | 67.1481 |
| GO:0045923 | positive regulation of fatty acid metabolic process | 1/9 | 48/29008 | 0.014796279 | 0.025893488 | 0.004563385 | Il1b | 1 | 1.8298 | 67.1481 |
| GO:0010677 | negative regulation of cellular carbohydrate metabolic process | 1/9 | 49/29008 | 0.015102454 | 0.026179175 | 0.004613733 | Il6 | 1 | 1.8210 | 65.7778 |
| GO:0032309 | icosanoid secretion | 1/9 | 49/29008 | 0.015102454 | 0.026179175 | 0.004613733 | Il1b | 1 | 1.8210 | 65.7778 |
| GO:0033173 | calcineurin-NFAT signaling cascade | 1/9 | 49/29008 | 0.015102454 | 0.026179175 | 0.004613733 | Tnf | 1 | 1.8210 | 65.7778 |
| GO:0071384 | cellular response to corticosteroid stimulus | 1/9 | 49/29008 | 0.015102454 | 0.026179175 | 0.004613733 | Fbxo32 | 1 | 1.8210 | 65.7778 |
| GO:1902622 | regulation of neutrophil migration | 1/9 | 49/29008 | 0.015102454 | 0.026179175 | 0.004613733 | Il1b | 1 | 1.8210 | 65.7778 |
| GO:1902930 | regulation of alcohol biosynthetic process | 1/9 | 49/29008 | 0.015102454 | 0.026179175 | 0.004613733 | Nfkb1 | 1 | 1.8210 | 65.7778 |
| GO:0035305 | negative regulation of dephosphorylation | 1/9 | 50/29008 | 0.015408545 | 0.026625772 | 0.00469244 | Tnf | 1 | 1.8122 | 64.4622 |
| GO:0042088 | T-helper 1 type immune response | 1/9 | 50/29008 | 0.015408545 | 0.026625772 | 0.00469244 | Il1b | 1 | 1.8122 | 64.4622 |
| GO:0032350 | regulation of hormone metabolic process | 1/9 | 51/29008 | 0.015714551 | 0.02702706 | 0.004763162 | Nfkb1 | 1 | 1.8037 | 63.1983 |
| GO:0046456 | icosanoid biosynthetic process | 1/9 | 51/29008 | 0.015714551 | 0.02702706 | 0.004763162 | Il1b | 1 | 1.8037 | 63.1983 |
| GO:0070527 | platelet aggregation | 1/9 | 51/29008 | 0.015714551 | 0.02702706 | 0.004763162 | Il6 | 1 | 1.8037 | 63.1983 |
| GO:0032715 | negative regulation of interleukin-6 production | 1/9 | 52/29008 | 0.016020472 | 0.027381803 | 0.00482568 | Tnf | 1 | 1.7953 | 61.9829 |
| GO:0042307 | positive regulation of protein import into nucleus | 1/9 | 52/29008 | 0.016020472 | 0.027381803 | 0.00482568 | Il6 | 1 | 1.7953 | 61.9829 |
| GO:0045124 | regulation of bone resorption | 1/9 | 52/29008 | 0.016020472 | 0.027381803 | 0.00482568 | Il6 | 1 | 1.7953 | 61.9829 |
| GO:0051955 | regulation of amino acid transport | 1/9 | 52/29008 | 0.016020472 | 0.027381803 | 0.00482568 | Il1b | 1 | 1.7953 | 61.9829 |
| GO:0010712 | regulation of collagen metabolic process | 1/9 | 53/29008 | 0.016326309 | 0.027604021 | 0.004864844 | Il6 | 1 | 1.7871 | 60.8134 |
| GO:0019646 | aerobic electron transport chain | 1/9 | 53/29008 | 0.016326309 | 0.027604021 | 0.004864844 | Ndufb8 | 1 | 1.7871 | 60.8134 |
| GO:0038066 | p38MAPK cascade | 1/9 | 53/29008 | 0.016326309 | 0.027604021 | 0.004864844 | Il1b | 1 | 1.7871 | 60.8134 |
| GO:0043370 | regulation of CD4-positive, alpha-beta T cell differentiation | 1/9 | 53/29008 | 0.016326309 | 0.027604021 | 0.004864844 | Il6 | 1 | 1.7871 | 60.8134 |
| GO:0046638 | positive regulation of alpha-beta T cell differentiation | 1/9 | 53/29008 | 0.016326309 | 0.027604021 | 0.004864844 | Il6 | 1 | 1.7871 | 60.8134 |
| GO:0097720 | calcineurin-mediated signaling | 1/9 | 53/29008 | 0.016326309 | 0.027604021 | 0.004864844 | Tnf | 1 | 1.7871 | 60.8134 |
| GO:2000351 | regulation of endothelial cell apoptotic process | 1/9 | 53/29008 | 0.016326309 | 0.027604021 | 0.004864844 | Tnf | 1 | 1.7871 | 60.8134 |
| GO:0002714 | positive regulation of B cell mediated immunity | 1/9 | 54/29008 | 0.016632062 | 0.027779082 | 0.004895696 | Tnf | 1 | 1.7791 | 59.6872 |
| GO:0002891 | positive regulation of immunoglobulin mediated immune response | 1/9 | 54/29008 | 0.016632062 | 0.027779082 | 0.004895696 | Tnf | 1 | 1.7791 | 59.6872 |
| GO:0006111 | regulation of gluconeogenesis | 1/9 | 54/29008 | 0.016632062 | 0.027779082 | 0.004895696 | Il6 | 1 | 1.7791 | 59.6872 |
| GO:0006692 | prostanoid metabolic process | 1/9 | 54/29008 | 0.016632062 | 0.027779082 | 0.004895696 | Il1b | 1 | 1.7791 | 59.6872 |
| GO:0006693 | prostaglandin metabolic process | 1/9 | 54/29008 | 0.016632062 | 0.027779082 | 0.004895696 | Il1b | 1 | 1.7791 | 59.6872 |
| GO:0031018 | endocrine pancreas development | 1/9 | 54/29008 | 0.016632062 | 0.027779082 | 0.004895696 | Il6 | 1 | 1.7791 | 59.6872 |
| GO:0032892 | positive regulation of organic acid transport | 1/9 | 54/29008 | 0.016632062 | 0.027779082 | 0.004895696 | Il1b | 1 | 1.7791 | 59.6872 |
| GO:0045912 | negative regulation of carbohydrate metabolic process | 1/9 | 54/29008 | 0.016632062 | 0.027779082 | 0.004895696 | Il6 | 1 | 1.7791 | 59.6872 |
| GO:0010596 | negative regulation of endothelial cell migration | 1/9 | 55/29008 | 0.01693773 | 0.028033984 | 0.004940619 | Tnf | 1 | 1.7711 | 58.6020 |
| GO:0031646 | positive regulation of nervous system process | 1/9 | 55/29008 | 0.01693773 | 0.028033984 | 0.004940619 | Il6 | 1 | 1.7711 | 58.6020 |
| GO:0032964 | collagen biosynthetic process | 1/9 | 55/29008 | 0.01693773 | 0.028033984 | 0.004940619 | Il6 | 1 | 1.7711 | 58.6020 |
| GO:0042531 | positive regulation of tyrosine phosphorylation of STAT protein | 1/9 | 55/29008 | 0.01693773 | 0.028033984 | 0.004940619 | Il6 | 1 | 1.7711 | 58.6020 |
| GO:0045687 | positive regulation of glial cell differentiation | 1/9 | 55/29008 | 0.01693773 | 0.028033984 | 0.004940619 | Il1b | 1 | 1.7711 | 58.6020 |
| GO:0140353 | lipid export from cell | 1/9 | 55/29008 | 0.01693773 | 0.028033984 | 0.004940619 | Il1b | 1 | 1.7711 | 58.6020 |
| GO:0010665 | regulation of cardiac muscle cell apoptotic process | 1/9 | 56/29008 | 0.017243313 | 0.028411396 | 0.005007133 | Fbxo32 | 1 | 1.7634 | 57.5556 |
| GO:0048016 | inositol phosphate-mediated signaling | 1/9 | 56/29008 | 0.017243313 | 0.028411396 | 0.005007133 | Tnf | 1 | 1.7634 | 57.5556 |
| GO:0072577 | endothelial cell apoptotic process | 1/9 | 56/29008 | 0.017243313 | 0.028411396 | 0.005007133 | Tnf | 1 | 1.7634 | 57.5556 |
| GO:0010257 | NADH dehydrogenase complex assembly | 1/9 | 57/29008 | 0.017548812 | 0.02869962 | 0.005057928 | Ndufb8 | 1 | 1.7558 | 56.5458 |
| GO:0032981 | mitochondrial respiratory chain complex I assembly | 1/9 | 57/29008 | 0.017548812 | 0.02869962 | 0.005057928 | Ndufb8 | 1 | 1.7558 | 56.5458 |
| GO:0043666 | regulation of phosphoprotein phosphatase activity | 1/9 | 57/29008 | 0.017548812 | 0.02869962 | 0.005057928 | Tnf | 1 | 1.7558 | 56.5458 |
| GO:0048634 | regulation of muscle organ development | 1/9 | 57/29008 | 0.017548812 | 0.02869962 | 0.005057928 | Il6 | 1 | 1.7558 | 56.5458 |
| GO:0071622 | regulation of granulocyte chemotaxis | 1/9 | 57/29008 | 0.017548812 | 0.02869962 | 0.005057928 | Il1b | 1 | 1.7558 | 56.5458 |
| GO:0045058 | T cell selection | 1/9 | 58/29008 | 0.017854227 | 0.02898345 | 0.00510795 | Il6 | 1 | 1.7483 | 55.5709 |
| GO:0045071 | negative regulation of viral genome replication | 1/9 | 58/29008 | 0.017854227 | 0.02898345 | 0.00510795 | Tnf | 1 | 1.7483 | 55.5709 |
| GO:0051055 | negative regulation of lipid biosynthetic process | 1/9 | 58/29008 | 0.017854227 | 0.02898345 | 0.00510795 | Nfkb1 | 1 | 1.7483 | 55.5709 |
| GO:0071715 | icosanoid transport | 1/9 | 58/29008 | 0.017854227 | 0.02898345 | 0.00510795 | Il1b | 1 | 1.7483 | 55.5709 |
| GO:1904591 | positive regulation of protein import | 1/9 | 58/29008 | 0.017854227 | 0.02898345 | 0.00510795 | Il6 | 1 | 1.7483 | 55.5709 |
| GO:0002639 | positive regulation of immunoglobulin production | 1/9 | 59/29008 | 0.018159557 | 0.029349049 | 0.005172382 | Il6 | 1 | 1.7409 | 54.6290 |
| GO:0010662 | regulation of striated muscle cell apoptotic process | 1/9 | 59/29008 | 0.018159557 | 0.029349049 | 0.005172382 | Fbxo32 | 1 | 1.7409 | 54.6290 |
| GO:1903793 | positive regulation of anion transport | 1/9 | 59/29008 | 0.018159557 | 0.029349049 | 0.005172382 | Il1b | 1 | 1.7409 | 54.6290 |
| GO:0006446 | regulation of translational initiation | 1/9 | 60/29008 | 0.018464803 | 0.029624553 | 0.005220936 | Tnf | 1 | 1.7337 | 53.7185 |
| GO:0010659 | cardiac muscle cell apoptotic process | 1/9 | 60/29008 | 0.018464803 | 0.029624553 | 0.005220936 | Fbxo32 | 1 | 1.7337 | 53.7185 |
| GO:0042987 | amyloid precursor protein catabolic process | 1/9 | 60/29008 | 0.018464803 | 0.029624553 | 0.005220936 | Tnf | 1 | 1.7337 | 53.7185 |
| GO:0070265 | necrotic cell death | 1/9 | 60/29008 | 0.018464803 | 0.029624553 | 0.005220936 | Tnf | 1 | 1.7337 | 53.7185 |
| GO:2001238 | positive regulation of extrinsic apoptotic signaling pathway | 1/9 | 60/29008 | 0.018464803 | 0.029624553 | 0.005220936 | Tnf | 1 | 1.7337 | 53.7185 |
| GO:0035176 | social behavior | 1/9 | 61/29008 | 0.018769965 | 0.03002648 | 0.00529177 | Il1b | 1 | 1.7265 | 52.8379 |
| GO:1904705 | regulation of vascular associated smooth muscle cell proliferation | 1/9 | 61/29008 | 0.018769965 | 0.03002648 | 0.00529177 | Tnf | 1 | 1.7265 | 52.8379 |
| GO:0001960 | negative regulation of cytokine-mediated signaling pathway | 1/9 | 62/29008 | 0.019075042 | 0.030206731 | 0.005323537 | Il6 | 1 | 1.7195 | 51.9857 |
| GO:0042446 | hormone biosynthetic process | 1/9 | 62/29008 | 0.019075042 | 0.030206731 | 0.005323537 | Nfkb1 | 1 | 1.7195 | 51.9857 |
| GO:0043407 | negative regulation of MAP kinase activity | 1/9 | 62/29008 | 0.019075042 | 0.030206731 | 0.005323537 | Il1b | 1 | 1.7195 | 51.9857 |
| GO:0046427 | positive regulation of receptor signaling pathway via JAK-STAT | 1/9 | 62/29008 | 0.019075042 | 0.030206731 | 0.005323537 | Il6 | 1 | 1.7195 | 51.9857 |
| GO:0046850 | regulation of bone remodeling | 1/9 | 62/29008 | 0.019075042 | 0.030206731 | 0.005323537 | Il6 | 1 | 1.7195 | 51.9857 |
| GO:1903078 | positive regulation of protein localization to plasma membrane | 1/9 | 62/29008 | 0.019075042 | 0.030206731 | 0.005323537 | Tnf | 1 | 1.7195 | 51.9857 |
| GO:1990874 | vascular associated smooth muscle cell proliferation | 1/9 | 62/29008 | 0.019075042 | 0.030206731 | 0.005323537 | Tnf | 1 | 1.7195 | 51.9857 |
| GO:0010658 | striated muscle cell apoptotic process | 1/9 | 63/29008 | 0.019380035 | 0.030470184 | 0.005369967 | Fbxo32 | 1 | 1.7126 | 51.1605 |
| GO:0030865 | cortical cytoskeleton organization | 1/9 | 63/29008 | 0.019380035 | 0.030470184 | 0.005369967 | Tnf | 1 | 1.7126 | 51.1605 |
| GO:0032653 | regulation of interleukin-10 production | 1/9 | 63/29008 | 0.019380035 | 0.030470184 | 0.005369967 | Il6 | 1 | 1.7126 | 51.1605 |
| GO:0032655 | regulation of interleukin-12 production | 1/9 | 63/29008 | 0.019380035 | 0.030470184 | 0.005369967 | Nfkb1 | 1 | 1.7126 | 51.1605 |
| GO:0051785 | positive regulation of nuclear division | 1/9 | 63/29008 | 0.019380035 | 0.030470184 | 0.005369967 | Il1b | 1 | 1.7126 | 51.1605 |
| GO:0045661 | regulation of myoblast differentiation | 1/9 | 64/29008 | 0.019684944 | 0.030773475 | 0.005423418 | Tnf | 1 | 1.7059 | 50.3611 |
| GO:0048512 | circadian behavior | 1/9 | 64/29008 | 0.019684944 | 0.030773475 | 0.005423418 | Il6 | 1 | 1.7059 | 50.3611 |
| GO:0050435 | amyloid-beta metabolic process | 1/9 | 64/29008 | 0.019684944 | 0.030773475 | 0.005423418 | Tnf | 1 | 1.7059 | 50.3611 |
| GO:0051703 | biological process involved in intraspecies interaction between organisms | 1/9 | 64/29008 | 0.019684944 | 0.030773475 | 0.005423418 | Il1b | 1 | 1.7059 | 50.3611 |
| GO:0032613 | interleukin-10 production | 1/9 | 65/29008 | 0.019989768 | 0.031029315 | 0.005468506 | Il6 | 1 | 1.6992 | 49.5863 |
| GO:0032615 | interleukin-12 production | 1/9 | 65/29008 | 0.019989768 | 0.031029315 | 0.005468506 | Nfkb1 | 1 | 1.6992 | 49.5863 |
| GO:0042775 | mitochondrial ATP synthesis coupled electron transport | 1/9 | 65/29008 | 0.019989768 | 0.031029315 | 0.005468506 | Ndufb8 | 1 | 1.6992 | 49.5863 |
| GO:0043331 | response to dsRNA | 1/9 | 65/29008 | 0.019989768 | 0.031029315 | 0.005468506 | Nfkb1 | 1 | 1.6992 | 49.5863 |
| GO:0060986 | endocrine hormone secretion | 1/9 | 65/29008 | 0.019989768 | 0.031029315 | 0.005468506 | Il1b | 1 | 1.6992 | 49.5863 |
| GO:0060761 | negative regulation of response to cytokine stimulus | 1/9 | 66/29008 | 0.020294508 | 0.031457919 | 0.005544042 | Il6 | 1 | 1.6926 | 48.8350 |
| GO:0007622 | rhythmic behavior | 1/9 | 67/29008 | 0.020599164 | 0.03179562 | 0.005603557 | Il6 | 1 | 1.6862 | 48.1061 |
| GO:0032663 | regulation of interleukin-2 production | 1/9 | 67/29008 | 0.020599164 | 0.03179562 | 0.005603557 | Il1b | 1 | 1.6862 | 48.1061 |
| GO:0042773 | ATP synthesis coupled electron transport | 1/9 | 67/29008 | 0.020599164 | 0.03179562 | 0.005603557 | Ndufb8 | 1 | 1.6862 | 48.1061 |
| GO:0045668 | negative regulation of osteoblast differentiation | 1/9 | 68/29008 | 0.020903736 | 0.032130357 | 0.00566255 | Tnf | 1 | 1.6798 | 47.3987 |
| GO:0060425 | lung morphogenesis | 1/9 | 68/29008 | 0.020903736 | 0.032130357 | 0.00566255 | Tnf | 1 | 1.6798 | 47.3987 |
| GO:1904894 | positive regulation of receptor signaling pathway via STAT | 1/9 | 68/29008 | 0.020903736 | 0.032130357 | 0.00566255 | Il6 | 1 | 1.6798 | 47.3987 |
| GO:0001937 | negative regulation of endothelial cell proliferation | 1/9 | 69/29008 | 0.021208223 | 0.03241702 | 0.005713071 | Tnf | 1 | 1.6735 | 46.7118 |
| GO:0015800 | acidic amino acid transport | 1/9 | 69/29008 | 0.021208223 | 0.03241702 | 0.005713071 | Il1b | 1 | 1.6735 | 46.7118 |
| GO:0042093 | T-helper cell differentiation | 1/9 | 69/29008 | 0.021208223 | 0.03241702 | 0.005713071 | Il6 | 1 | 1.6735 | 46.7118 |
| GO:1904377 | positive regulation of protein localization to cell periphery | 1/9 | 69/29008 | 0.021208223 | 0.03241702 | 0.005713071 | Tnf | 1 | 1.6735 | 46.7118 |
| GO:0002294 | CD4-positive, alpha-beta T cell differentiation involved in immune response | 1/9 | 70/29008 | 0.021512626 | 0.032655216 | 0.00575505 | Il6 | 1 | 1.6673 | 46.0444 |
| GO:0042509 | regulation of tyrosine phosphorylation of STAT protein | 1/9 | 70/29008 | 0.021512626 | 0.032655216 | 0.00575505 | Il6 | 1 | 1.6673 | 46.0444 |
| GO:0046324 | regulation of glucose import | 1/9 | 70/29008 | 0.021512626 | 0.032655216 | 0.00575505 | Tnf | 1 | 1.6673 | 46.0444 |
| GO:0097194 | execution phase of apoptosis | 1/9 | 70/29008 | 0.021512626 | 0.032655216 | 0.00575505 | Il6 | 1 | 1.6673 | 46.0444 |
| GO:2000514 | regulation of CD4-positive, alpha-beta T cell activation | 1/9 | 70/29008 | 0.021512626 | 0.032655216 | 0.00575505 | Il6 | 1 | 1.6673 | 46.0444 |
| GO:0002293 | alpha-beta T cell differentiation involved in immune response | 1/9 | 71/29008 | 0.021816945 | 0.03307148 | 0.005828411 | Il6 | 1 | 1.6612 | 45.3959 |
| GO:0000271 | polysaccharide biosynthetic process | 1/9 | 72/29008 | 0.02212118 | 0.033257424 | 0.005861181 | Nfkb1 | 1 | 1.6552 | 44.7654 |
| GO:0002287 | alpha-beta T cell activation involved in immune response | 1/9 | 72/29008 | 0.02212118 | 0.033257424 | 0.005861181 | Il6 | 1 | 1.6552 | 44.7654 |
| GO:0002763 | positive regulation of myeloid leukocyte differentiation | 1/9 | 72/29008 | 0.02212118 | 0.033257424 | 0.005861181 | Tnf | 1 | 1.6552 | 44.7654 |
| GO:0010633 | negative regulation of epithelial cell migration | 1/9 | 72/29008 | 0.02212118 | 0.033257424 | 0.005861181 | Tnf | 1 | 1.6552 | 44.7654 |
| GO:0061045 | negative regulation of wound healing | 1/9 | 72/29008 | 0.02212118 | 0.033257424 | 0.005861181 | Tnf | 1 | 1.6552 | 44.7654 |
| GO:1905330 | regulation of morphogenesis of an epithelium | 1/9 | 72/29008 | 0.02212118 | 0.033257424 | 0.005861181 | Tnf | 1 | 1.6552 | 44.7654 |
| GO:0033209 | tumor necrosis factor-mediated signaling pathway | 1/9 | 73/29008 | 0.022425331 | 0.033485651 | 0.005901403 | Tnf | 1 | 1.6493 | 44.1522 |
| GO:0034394 | protein localization to cell surface | 1/9 | 73/29008 | 0.022425331 | 0.033485651 | 0.005901403 | Tnf | 1 | 1.6493 | 44.1522 |
| GO:0046635 | positive regulation of alpha-beta T cell activation | 1/9 | 73/29008 | 0.022425331 | 0.033485651 | 0.005901403 | Il6 | 1 | 1.6493 | 44.1522 |
| GO:0046637 | regulation of alpha-beta T cell differentiation | 1/9 | 73/29008 | 0.022425331 | 0.033485651 | 0.005901403 | Il6 | 1 | 1.6493 | 44.1522 |
| GO:1902117 | positive regulation of organelle assembly | 1/9 | 73/29008 | 0.022425331 | 0.033485651 | 0.005901403 | Tnf | 1 | 1.6493 | 44.1522 |
| GO:0002437 | inflammatory response to antigenic stimulus | 1/9 | 74/29008 | 0.022729398 | 0.03366524 | 0.005933053 | Tnf | 1 | 1.6434 | 43.5556 |
| GO:0007260 | tyrosine phosphorylation of STAT protein | 1/9 | 74/29008 | 0.022729398 | 0.03366524 | 0.005933053 | Il6 | 1 | 1.6434 | 43.5556 |
| GO:0010611 | regulation of cardiac muscle hypertrophy | 1/9 | 74/29008 | 0.022729398 | 0.03366524 | 0.005933053 | Fbxo32 | 1 | 1.6434 | 43.5556 |
| GO:0032623 | interleukin-2 production | 1/9 | 74/29008 | 0.022729398 | 0.03366524 | 0.005933053 | Il1b | 1 | 1.6434 | 43.5556 |
| GO:0046824 | positive regulation of nucleocytoplasmic transport | 1/9 | 74/29008 | 0.022729398 | 0.03366524 | 0.005933053 | Il6 | 1 | 1.6434 | 43.5556 |
| GO:0050688 | regulation of defense response to virus | 1/9 | 74/29008 | 0.022729398 | 0.03366524 | 0.005933053 | Il1b | 1 | 1.6434 | 43.5556 |
| GO:0042982 | amyloid precursor protein metabolic process | 1/9 | 75/29008 | 0.023033381 | 0.034069563 | 0.00600431 | Tnf | 1 | 1.6376 | 42.9748 |
| GO:0002712 | regulation of B cell mediated immunity | 1/9 | 76/29008 | 0.023337279 | 0.034426403 | 0.006067198 | Tnf | 1 | 1.6319 | 42.4094 |
| GO:0002889 | regulation of immunoglobulin mediated immune response | 1/9 | 76/29008 | 0.023337279 | 0.034426403 | 0.006067198 | Tnf | 1 | 1.6319 | 42.4094 |
| GO:0014743 | regulation of muscle hypertrophy | 1/9 | 77/29008 | 0.023641094 | 0.034781208 | 0.006129728 | Fbxo32 | 1 | 1.6263 | 41.8586 |
| GO:0045669 | positive regulation of osteoblast differentiation | 1/9 | 77/29008 | 0.023641094 | 0.034781208 | 0.006129728 | Il6 | 1 | 1.6263 | 41.8586 |
| GO:0002292 | T cell differentiation involved in immune response | 1/9 | 78/29008 | 0.023944825 | 0.034993833 | 0.0061672 | Il6 | 1 | 1.6208 | 41.3219 |
| GO:0006919 | activation of cysteine-type endopeptidase activity involved in apoptotic process | 1/9 | 78/29008 | 0.023944825 | 0.034993833 | 0.0061672 | Tnf | 1 | 1.6208 | 41.3219 |
| GO:0034109 | homotypic cell-cell adhesion | 1/9 | 78/29008 | 0.023944825 | 0.034993833 | 0.0061672 | Il6 | 1 | 1.6208 | 41.3219 |
| GO:0042306 | regulation of protein import into nucleus | 1/9 | 78/29008 | 0.023944825 | 0.034993833 | 0.0061672 | Il6 | 1 | 1.6208 | 41.3219 |
| GO:0045453 | bone resorption | 1/9 | 78/29008 | 0.023944825 | 0.034993833 | 0.0061672 | Il6 | 1 | 1.6208 | 41.3219 |
| GO:0006094 | gluconeogenesis | 1/9 | 79/29008 | 0.024248471 | 0.035296781 | 0.006220591 | Il6 | 1 | 1.6153 | 40.7989 |
| GO:0046626 | regulation of insulin receptor signaling pathway | 1/9 | 79/29008 | 0.024248471 | 0.035296781 | 0.006220591 | Il1b | 1 | 1.6153 | 40.7989 |
| GO:0050709 | negative regulation of protein secretion | 1/9 | 79/29008 | 0.024248471 | 0.035296781 | 0.006220591 | Il1b | 1 | 1.6153 | 40.7989 |
| GO:0045123 | cellular extravasation | 1/9 | 80/29008 | 0.024552034 | 0.03559721 | 0.006273537 | Tnf | 1 | 1.6099 | 40.2889 |
| GO:0045670 | regulation of osteoclast differentiation | 1/9 | 80/29008 | 0.024552034 | 0.03559721 | 0.006273537 | Tnf | 1 | 1.6099 | 40.2889 |
| GO:1900076 | regulation of cellular response to insulin stimulus | 1/9 | 80/29008 | 0.024552034 | 0.03559721 | 0.006273537 | Il1b | 1 | 1.6099 | 40.2889 |
| GO:0045913 | positive regulation of carbohydrate metabolic process | 1/9 | 81/29008 | 0.024855512 | 0.035942379 | 0.006334369 | Nfkb1 | 1 | 1.6046 | 39.7915 |
| GO:0070830 | bicellular tight junction assembly | 1/9 | 81/29008 | 0.024855512 | 0.035942379 | 0.006334369 | Tnf | 1 | 1.6046 | 39.7915 |
| GO:0008625 | extrinsic apoptotic signaling pathway via death domain receptors | 1/9 | 82/29008 | 0.025158907 | 0.036285616 | 0.00639486 | Tnf | 1 | 1.5993 | 39.3062 |
| GO:0019319 | hexose biosynthetic process | 1/9 | 82/29008 | 0.025158907 | 0.036285616 | 0.00639486 | Il6 | 1 | 1.5993 | 39.3062 |
| GO:0048708 | astrocyte differentiation | 1/9 | 83/29008 | 0.025462218 | 0.036626934 | 0.006455013 | Il1b | 1 | 1.5941 | 38.8327 |
| GO:0050848 | regulation of calcium-mediated signaling | 1/9 | 83/29008 | 0.025462218 | 0.036626934 | 0.006455013 | Tnf | 1 | 1.5941 | 38.8327 |
| GO:0034103 | regulation of tissue remodeling | 1/9 | 84/29008 | 0.025765445 | 0.03696635 | 0.00651483 | Il6 | 1 | 1.5890 | 38.3704 |
| GO:1904589 | regulation of protein import | 1/9 | 84/29008 | 0.025765445 | 0.03696635 | 0.00651483 | Il6 | 1 | 1.5890 | 38.3704 |
| GO:0002251 | organ or tissue specific immune response | 1/9 | 85/29008 | 0.026068588 | 0.037206985 | 0.006557239 | Il6 | 1 | 1.5839 | 37.9190 |
| GO:0010921 | regulation of phosphatase activity | 1/9 | 85/29008 | 0.026068588 | 0.037206985 | 0.006557239 | Tnf | 1 | 1.5839 | 37.9190 |
| GO:0043648 | dicarboxylic acid metabolic process | 1/9 | 85/29008 | 0.026068588 | 0.037206985 | 0.006557239 | Sdhb | 1 | 1.5839 | 37.9190 |
| GO:0050805 | negative regulation of synaptic transmission | 1/9 | 85/29008 | 0.026068588 | 0.037206985 | 0.006557239 | Il1b | 1 | 1.5839 | 37.9190 |
| GO:0030168 | platelet activation | 1/9 | 86/29008 | 0.026371647 | 0.037493455 | 0.006607725 | Il6 | 1 | 1.5789 | 37.4780 |
| GO:0046323 | glucose import | 1/9 | 86/29008 | 0.026371647 | 0.037493455 | 0.006607725 | Tnf | 1 | 1.5789 | 37.4780 |
| GO:0120192 | tight junction assembly | 1/9 | 86/29008 | 0.026371647 | 0.037493455 | 0.006607725 | Tnf | 1 | 1.5789 | 37.4780 |
| GO:0015908 | fatty acid transport | 1/9 | 87/29008 | 0.026674623 | 0.037826336 | 0.006666391 | Il1b | 1 | 1.5739 | 37.0473 |
| GO:0032890 | regulation of organic acid transport | 1/9 | 87/29008 | 0.026674623 | 0.037826336 | 0.006666391 | Il1b | 1 | 1.5739 | 37.0473 |
| GO:0046364 | monosaccharide biosynthetic process | 1/9 | 89/29008 | 0.027280322 | 0.038585681 | 0.006800216 | Il6 | 1 | 1.5642 | 36.2147 |
| GO:0046683 | response to organophosphorus | 1/9 | 89/29008 | 0.027280322 | 0.038585681 | 0.006800216 | Il1b | 1 | 1.5642 | 36.2147 |
| GO:0006835 | dicarboxylic acid transport | 1/9 | 90/29008 | 0.027583046 | 0.038814043 | 0.006840462 | Il1b | 1 | 1.5594 | 35.8123 |
| GO:0043297 | apical junction assembly | 1/9 | 90/29008 | 0.027583046 | 0.038814043 | 0.006840462 | Tnf | 1 | 1.5594 | 35.8123 |
| GO:0045069 | regulation of viral genome replication | 1/9 | 90/29008 | 0.027583046 | 0.038814043 | 0.006840462 | Tnf | 1 | 1.5594 | 35.8123 |
| GO:0045665 | negative regulation of neuron differentiation | 1/9 | 90/29008 | 0.027583046 | 0.038814043 | 0.006840462 | Il1b | 1 | 1.5594 | 35.8123 |
| GO:0031016 | pancreas development | 1/9 | 91/29008 | 0.027885687 | 0.039039961 | 0.006880277 | Il6 | 1 | 1.5546 | 35.4188 |
| GO:0043242 | negative regulation of protein-containing complex disassembly | 1/9 | 91/29008 | 0.027885687 | 0.039039961 | 0.006880277 | Tnf | 1 | 1.5546 | 35.4188 |
| GO:0070301 | cellular response to hydrogen peroxide | 1/9 | 91/29008 | 0.027885687 | 0.039039961 | 0.006880277 | Il6 | 1 | 1.5546 | 35.4188 |
| GO:1903035 | negative regulation of response to wounding | 1/9 | 91/29008 | 0.027885687 | 0.039039961 | 0.006880277 | Tnf | 1 | 1.5546 | 35.4188 |
| GO:0043367 | CD4-positive, alpha-beta T cell differentiation | 1/9 | 92/29008 | 0.028188243 | 0.039263472 | 0.006919667 | Il6 | 1 | 1.5499 | 35.0338 |
| GO:0120193 | tight junction organization | 1/9 | 92/29008 | 0.028188243 | 0.039263472 | 0.006919667 | Tnf | 1 | 1.5499 | 35.0338 |
| GO:1900182 | positive regulation of protein localization to nucleus | 1/9 | 92/29008 | 0.028188243 | 0.039263472 | 0.006919667 | Il6 | 1 | 1.5499 | 35.0338 |
| GO:1904035 | regulation of epithelial cell apoptotic process | 1/9 | 92/29008 | 0.028188243 | 0.039263472 | 0.006919667 | Tnf | 1 | 1.5499 | 35.0338 |
| GO:0019226 | transmission of nerve impulse | 1/9 | 93/29008 | 0.028490716 | 0.039534466 | 0.006967427 | Il6 | 1 | 1.5453 | 34.6571 |
| GO:0035304 | regulation of protein dephosphorylation | 1/9 | 93/29008 | 0.028490716 | 0.039534466 | 0.006967427 | Tnf | 1 | 1.5453 | 34.6571 |
| GO:0043535 | regulation of blood vessel endothelial cell migration | 1/9 | 93/29008 | 0.028490716 | 0.039534466 | 0.006967427 | Tnf | 1 | 1.5453 | 34.6571 |
| GO:0002312 | B cell activation involved in immune response | 1/9 | 94/29008 | 0.028793105 | 0.039903686 | 0.007032497 | Il6 | 1 | 1.5407 | 34.2884 |
| GO:0010660 | regulation of muscle cell apoptotic process | 1/9 | 95/29008 | 0.029095411 | 0.040170674 | 0.00707955 | Fbxo32 | 1 | 1.5362 | 33.9275 |
| GO:0033077 | T cell differentiation in thymus | 1/9 | 95/29008 | 0.029095411 | 0.040170674 | 0.00707955 | Il1b | 1 | 1.5362 | 33.9275 |
| GO:0045685 | regulation of glial cell differentiation | 1/9 | 95/29008 | 0.029095411 | 0.040170674 | 0.00707955 | Il1b | 1 | 1.5362 | 33.9275 |
| GO:0045445 | myoblast differentiation | 1/9 | 97/29008 | 0.029699771 | 0.040902316 | 0.007208492 | Tnf | 1 | 1.5272 | 33.2279 |
| GO:0051781 | positive regulation of cell division | 1/9 | 97/29008 | 0.029699771 | 0.040902316 | 0.007208492 | Il1b | 1 | 1.5272 | 33.2279 |
| GO:0002690 | positive regulation of leukocyte chemotaxis | 1/9 | 98/29008 | 0.030001826 | 0.041266591 | 0.007272691 | Il1b | 1 | 1.5229 | 32.8889 |
| GO:0010657 | muscle cell apoptotic process | 1/9 | 100/29008 | 0.030605684 | 0.041992069 | 0.007400547 | Fbxo32 | 1 | 1.5142 | 32.2311 |
| GO:0032370 | positive regulation of lipid transport | 1/9 | 100/29008 | 0.030605684 | 0.041992069 | 0.007400547 | Il1b | 1 | 1.5142 | 32.2311 |
| GO:0042116 | macrophage activation | 1/9 | 101/29008 | 0.030907488 | 0.042247922 | 0.007445637 | Tnf | 1 | 1.5099 | 31.9120 |
| GO:0043502 | regulation of muscle adaptation | 1/9 | 101/29008 | 0.030907488 | 0.042247922 | 0.007445637 | Fbxo32 | 1 | 1.5099 | 31.9120 |
| GO:0050795 | regulation of behavior | 1/9 | 101/29008 | 0.030907488 | 0.042247922 | 0.007445637 | Il6 | 1 | 1.5099 | 31.9120 |
| GO:0006352 | DNA-templated transcription, initiation | 1/9 | 102/29008 | 0.031209209 | 0.042449159 | 0.007481103 | Tfam | 1 | 1.5057 | 31.5991 |
| GO:0019217 | regulation of fatty acid metabolic process | 1/9 | 102/29008 | 0.031209209 | 0.042449159 | 0.007481103 | Il1b | 1 | 1.5057 | 31.5991 |
| GO:0030593 | neutrophil chemotaxis | 1/9 | 102/29008 | 0.031209209 | 0.042449159 | 0.007481103 | Il1b | 1 | 1.5057 | 31.5991 |
| GO:0048525 | negative regulation of viral process | 1/9 | 102/29008 | 0.031209209 | 0.042449159 | 0.007481103 | Tnf | 1 | 1.5057 | 31.5991 |
| GO:0003300 | cardiac muscle hypertrophy | 1/9 | 104/29008 | 0.031812399 | 0.043162749 | 0.007606863 | Fbxo32 | 1 | 1.4974 | 30.9915 |
| GO:0045582 | positive regulation of T cell differentiation | 1/9 | 104/29008 | 0.031812399 | 0.043162749 | 0.007606863 | Il6 | 1 | 1.4974 | 30.9915 |
| GO:0014074 | response to purine-containing compound | 1/9 | 105/29008 | 0.032113869 | 0.043464461 | 0.007660036 | Il1b | 1 | 1.4933 | 30.6963 |
| GO:0046849 | bone remodeling | 1/9 | 105/29008 | 0.032113869 | 0.043464461 | 0.007660036 | Il6 | 1 | 1.4933 | 30.6963 |
| GO:0050886 | endocrine process | 1/9 | 107/29008 | 0.032716559 | 0.044225705 | 0.007794195 | Il1b | 1 | 1.4852 | 30.1225 |
| GO:0014897 | striated muscle hypertrophy | 1/9 | 108/29008 | 0.033017778 | 0.044468797 | 0.007837037 | Fbxo32 | 1 | 1.4813 | 29.8436 |
| GO:0071901 | negative regulation of protein serine/threonine kinase activity | 1/9 | 108/29008 | 0.033017778 | 0.044468797 | 0.007837037 | Il1b | 1 | 1.4813 | 29.8436 |
| GO:0090263 | positive regulation of canonical Wnt signaling pathway | 1/9 | 108/29008 | 0.033017778 | 0.044468797 | 0.007837037 | Nfkb1 | 1 | 1.4813 | 29.8436 |
| GO:0140053 | mitochondrial gene expression | 1/9 | 109/29008 | 0.033318915 | 0.044819446 | 0.007898834 | Tfam | 1 | 1.4773 | 29.5698 |
| GO:0005976 | polysaccharide metabolic process | 1/9 | 110/29008 | 0.033619968 | 0.044894708 | 0.007912098 | Nfkb1 | 1 | 1.4734 | 29.3010 |
| GO:0006275 | regulation of DNA replication | 1/9 | 110/29008 | 0.033619968 | 0.044894708 | 0.007912098 | Il6 | 1 | 1.4734 | 29.3010 |
| GO:0007088 | regulation of mitotic nuclear division | 1/9 | 110/29008 | 0.033619968 | 0.044894708 | 0.007912098 | Il1b | 1 | 1.4734 | 29.3010 |
| GO:0014896 | muscle hypertrophy | 1/9 | 110/29008 | 0.033619968 | 0.044894708 | 0.007912098 | Fbxo32 | 1 | 1.4734 | 29.3010 |
| GO:0016525 | negative regulation of angiogenesis | 1/9 | 110/29008 | 0.033619968 | 0.044894708 | 0.007912098 | Tnf | 1 | 1.4734 | 29.3010 |
| GO:0032760 | positive regulation of tumor necrosis factor production | 1/9 | 110/29008 | 0.033619968 | 0.044894708 | 0.007912098 | Il6 | 1 | 1.4734 | 29.3010 |
| GO:0046634 | regulation of alpha-beta T cell activation | 1/9 | 111/29008 | 0.033920937 | 0.0451868 | 0.007963576 | Il6 | 1 | 1.4695 | 29.0370 |
| GO:1905477 | positive regulation of protein localization to membrane | 1/9 | 111/29008 | 0.033920937 | 0.0451868 | 0.007963576 | Tnf | 1 | 1.4695 | 29.0370 |
| GO:0045639 | positive regulation of myeloid cell differentiation | 1/9 | 112/29008 | 0.034221824 | 0.045422445 | 0.008005105 | Tnf | 1 | 1.4657 | 28.7778 |
| GO:1903557 | positive regulation of tumor necrosis factor superfamily cytokine production | 1/9 | 112/29008 | 0.034221824 | 0.045422445 | 0.008005105 | Il6 | 1 | 1.4657 | 28.7778 |
| GO:2000181 | negative regulation of blood vessel morphogenesis | 1/9 | 112/29008 | 0.034221824 | 0.045422445 | 0.008005105 | Tnf | 1 | 1.4657 | 28.7778 |
| GO:0006119 | oxidative phosphorylation | 1/9 | 113/29008 | 0.034522627 | 0.045656278 | 0.008046315 | Ndufb8 | 1 | 1.4619 | 28.5231 |
| GO:0008630 | intrinsic apoptotic signaling pathway in response to DNA damage | 1/9 | 113/29008 | 0.034522627 | 0.045656278 | 0.008046315 | Tnf | 1 | 1.4619 | 28.5231 |
| GO:1901343 | negative regulation of vasculature development | 1/9 | 113/29008 | 0.034522627 | 0.045656278 | 0.008046315 | Tnf | 1 | 1.4619 | 28.5231 |
| GO:0001942 | hair follicle development | 1/9 | 115/29008 | 0.035123983 | 0.046284481 | 0.008157027 | Tnf | 1 | 1.4544 | 28.0271 |
| GO:0035710 | CD4-positive, alpha-beta T cell activation | 1/9 | 115/29008 | 0.035123983 | 0.046284481 | 0.008157027 | Il6 | 1 | 1.4544 | 28.0271 |
| GO:1903076 | regulation of protein localization to plasma membrane | 1/9 | 115/29008 | 0.035123983 | 0.046284481 | 0.008157027 | Tnf | 1 | 1.4544 | 28.0271 |
| GO:0030316 | osteoclast differentiation | 1/9 | 116/29008 | 0.035424536 | 0.04651322 | 0.00819734 | Tnf | 1 | 1.4507 | 27.7854 |
| GO:0043534 | blood vessel endothelial cell migration | 1/9 | 116/29008 | 0.035424536 | 0.04651322 | 0.00819734 | Tnf | 1 | 1.4507 | 27.7854 |
| GO:0051952 | regulation of amine transport | 1/9 | 116/29008 | 0.035424536 | 0.04651322 | 0.00819734 | Il1b | 1 | 1.4507 | 27.7854 |
| GO:0006413 | translational initiation | 1/9 | 117/29008 | 0.035725006 | 0.046795926 | 0.008247163 | Tnf | 1 | 1.4470 | 27.5480 |
| GO:0044070 | regulation of anion transport | 1/9 | 117/29008 | 0.035725006 | 0.046795926 | 0.008247163 | Il1b | 1 | 1.4470 | 27.5480 |
| GO:0021782 | glial cell development | 1/9 | 119/29008 | 0.036325696 | 0.0472449 | 0.008326289 | Il1b | 1 | 1.4398 | 27.0850 |
| GO:0022404 | molting cycle process | 1/9 | 119/29008 | 0.036325696 | 0.0472449 | 0.008326289 | Tnf | 1 | 1.4398 | 27.0850 |
| GO:0022405 | hair cycle process | 1/9 | 119/29008 | 0.036325696 | 0.0472449 | 0.008326289 | Tnf | 1 | 1.4398 | 27.0850 |
| GO:0032963 | collagen metabolic process | 1/9 | 119/29008 | 0.036325696 | 0.0472449 | 0.008326289 | Il6 | 1 | 1.4398 | 27.0850 |
| GO:0045666 | positive regulation of neuron differentiation | 1/9 | 119/29008 | 0.036325696 | 0.0472449 | 0.008326289 | Il6 | 1 | 1.4398 | 27.0850 |
| GO:0098773 | skin epidermis development | 1/9 | 119/29008 | 0.036325696 | 0.0472449 | 0.008326289 | Tnf | 1 | 1.4398 | 27.0850 |
| GO:0001776 | leukocyte homeostasis | 1/9 | 120/29008 | 0.036625917 | 0.047410934 | 0.00835555 | Il6 | 1 | 1.4362 | 26.8593 |
| GO:0045621 | positive regulation of lymphocyte differentiation | 1/9 | 120/29008 | 0.036625917 | 0.047410934 | 0.00835555 | Il6 | 1 | 1.4362 | 26.8593 |
| GO:1904019 | epithelial cell apoptotic process | 1/9 | 120/29008 | 0.036625917 | 0.047410934 | 0.00835555 | Tnf | 1 | 1.4362 | 26.8593 |
| GO:2000177 | regulation of neural precursor cell proliferation | 1/9 | 120/29008 | 0.036625917 | 0.047410934 | 0.00835555 | Il1b | 1 | 1.4362 | 26.8593 |
| GO:0015718 | monocarboxylic acid transport | 1/9 | 121/29008 | 0.036926054 | 0.047687113 | 0.008404223 | Il1b | 1 | 1.4327 | 26.6373 |
| GO:0015837 | amine transport | 1/9 | 121/29008 | 0.036926054 | 0.047687113 | 0.008404223 | Il1b | 1 | 1.4327 | 26.6373 |
| GO:0001959 | regulation of cytokine-mediated signaling pathway | 1/9 | 122/29008 | 0.037226108 | 0.047961891 | 0.008452649 | Il6 | 1 | 1.4292 | 26.4189 |
| GO:0042542 | response to hydrogen peroxide | 1/9 | 122/29008 | 0.037226108 | 0.047961891 | 0.008452649 | Il6 | 1 | 1.4292 | 26.4189 |
| GO:1905954 | positive regulation of lipid localization | 1/9 | 123/29008 | 0.037526079 | 0.048291758 | 0.008510783 | Il1b | 1 | 1.4257 | 26.2042 |
| GO:0002286 | T cell activation involved in immune response | 1/9 | 124/29008 | 0.037825967 | 0.048563947 | 0.008558753 | Il6 | 1 | 1.4222 | 25.9928 |
| GO:0002688 | regulation of leukocyte chemotaxis | 1/9 | 124/29008 | 0.037825967 | 0.048563947 | 0.008558753 | Il1b | 1 | 1.4222 | 25.9928 |
| GO:0019079 | viral genome replication | 1/9 | 126/29008 | 0.038425494 | 0.049276101 | 0.008684261 | Tnf | 1 | 1.4154 | 25.5802 |
| GO:0008286 | insulin receptor signaling pathway | 1/9 | 127/29008 | 0.038725133 | 0.049372299 | 0.008701214 | Il1b | 1 | 1.4120 | 25.3788 |
| GO:0043280 | positive regulation of cysteine-type endopeptidase activity involved in apoptotic process | 1/9 | 127/29008 | 0.038725133 | 0.049372299 | 0.008701214 | Tnf | 1 | 1.4120 | 25.3788 |
| GO:0043500 | muscle adaptation | 1/9 | 127/29008 | 0.038725133 | 0.049372299 | 0.008701214 | Fbxo32 | 1 | 1.4120 | 25.3788 |
| GO:0046425 | regulation of receptor signaling pathway via JAK-STAT | 1/9 | 127/29008 | 0.038725133 | 0.049372299 | 0.008701214 | Il6 | 1 | 1.4120 | 25.3788 |
| GO:0071621 | granulocyte chemotaxis | 1/9 | 127/29008 | 0.038725133 | 0.049372299 | 0.008701214 | Il1b | 1 | 1.4120 | 25.3788 |
| GO:0001889 | liver development | 1/9 | 128/29008 | 0.039024689 | 0.049581657 | 0.008738111 | Il6 | 1 | 1.4087 | 25.1806 |
| GO:0042752 | regulation of circadian rhythm | 1/9 | 128/29008 | 0.039024689 | 0.049581657 | 0.008738111 | Il6 | 1 | 1.4087 | 25.1806 |
| GO:1990266 | neutrophil migration | 1/9 | 128/29008 | 0.039024689 | 0.049581657 | 0.008738111 | Il1b | 1 | 1.4087 | 25.1806 |
| GO:0043244 | regulation of protein-containing complex disassembly | 1/9 | 129/29008 | 0.039324162 | 0.049789463 | 0.008774734 | Tnf | 1 | 1.4053 | 24.9854 |
| GO:0046632 | alpha-beta T cell differentiation | 1/9 | 129/29008 | 0.039324162 | 0.049789463 | 0.008774734 | Il6 | 1 | 1.4053 | 24.9854 |
| GO:0046822 | regulation of nucleocytoplasmic transport | 1/9 | 129/29008 | 0.039324162 | 0.049789463 | 0.008774734 | Il6 | 1 | 1.4053 | 24.9854 |
| GO:0002698 | negative regulation of immune effector process | 1/9 | 130/29008 | 0.039623552 | 0.0500532 | 0.008821214 | Tnf | 1 | 1.4020 | 24.7932 |
| GO:0061041 | regulation of wound healing | 1/9 | 130/29008 | 0.039623552 | 0.0500532 | 0.008821214 | Tnf | 1 | 1.4020 | 24.7932 |
| GO:0010906 | regulation of glucose metabolic process | 1/9 | 131/29008 | 0.039922859 | 0.050315622 | 0.008867463 | Il6 | 1 | 1.3988 | 24.6039 |
| GO:0061008 | hepaticobiliary system development | 1/9 | 131/29008 | 0.039922859 | 0.050315622 | 0.008867463 | Il6 | 1 | 1.3988 | 24.6039 |
| GO:0060759 | regulation of response to cytokine stimulus | 1/9 | 132/29008 | 0.040222083 | 0.050576738 | 0.008913481 | Il6 | 1 | 1.3955 | 24.4175 |
| GO:2000027 | regulation of animal organ morphogenesis | 1/9 | 132/29008 | 0.040222083 | 0.050576738 | 0.008913481 | Tnf | 1 | 1.3955 | 24.4175 |
| GO:0033559 | unsaturated fatty acid metabolic process | 1/9 | 133/29008 | 0.040521224 | 0.050778592 | 0.008949055 | Il1b | 1 | 1.3923 | 24.2339 |
| GO:0035303 | regulation of dephosphorylation | 1/9 | 133/29008 | 0.040521224 | 0.050778592 | 0.008949055 | Tnf | 1 | 1.3923 | 24.2339 |
| GO:0090305 | nucleic acid phosphodiester bond hydrolysis | 1/9 | 133/29008 | 0.040521224 | 0.050778592 | 0.008949055 | Il6 | 1 | 1.3923 | 24.2339 |
| GO:1904892 | regulation of receptor signaling pathway via STAT | 1/9 | 135/29008 | 0.041119258 | 0.051469322 | 0.009070787 | Il6 | 1 | 1.3860 | 23.8749 |
| GO:0002761 | regulation of myeloid leukocyte differentiation | 1/9 | 137/29008 | 0.041716961 | 0.052158066 | 0.009192169 | Tnf | 1 | 1.3797 | 23.5264 |
| GO:0042303 | molting cycle | 1/9 | 138/29008 | 0.042015688 | 0.05235288 | 0.009226503 | Tnf | 1 | 1.3766 | 23.3559 |
| GO:0042633 | hair cycle | 1/9 | 138/29008 | 0.042015688 | 0.05235288 | 0.009226503 | Tnf | 1 | 1.3766 | 23.3559 |
| GO:0046165 | alcohol biosynthetic process | 1/9 | 138/29008 | 0.042015688 | 0.05235288 | 0.009226503 | Nfkb1 | 1 | 1.3766 | 23.3559 |
| GO:0030177 | positive regulation of Wnt signaling pathway | 1/9 | 139/29008 | 0.042314332 | 0.052605713 | 0.009271061 | Nfkb1 | 1 | 1.3735 | 23.1878 |
| GO:0051224 | negative regulation of protein transport | 1/9 | 139/29008 | 0.042314332 | 0.052605713 | 0.009271061 | Il1b | 1 | 1.3735 | 23.1878 |
| GO:0034614 | cellular response to reactive oxygen species | 1/9 | 140/29008 | 0.042612893 | 0.052857302 | 0.0093154 | Il6 | 1 | 1.3705 | 23.0222 |
| GO:0071887 | leukocyte apoptotic process | 1/9 | 140/29008 | 0.042612893 | 0.052857302 | 0.0093154 | Il6 | 1 | 1.3705 | 23.0222 |
| GO:0035270 | endocrine system development | 1/9 | 141/29008 | 0.042911372 | 0.053047916 | 0.009348993 | Il6 | 1 | 1.3674 | 22.8589 |
| GO:1901655 | cellular response to ketone | 1/9 | 141/29008 | 0.042911372 | 0.053047916 | 0.009348993 | Fbxo32 | 1 | 1.3674 | 22.8589 |
| GO:1904375 | regulation of protein localization to cell periphery | 1/9 | 141/29008 | 0.042911372 | 0.053047916 | 0.009348993 | Tnf | 1 | 1.3674 | 22.8589 |
| GO:0006690 | icosanoid metabolic process | 1/9 | 142/29008 | 0.043209768 | 0.053356781 | 0.009403427 | Il1b | 1 | 1.3644 | 22.6980 |
| GO:0050728 | negative regulation of inflammatory response | 1/9 | 143/29008 | 0.043508081 | 0.053604688 | 0.009447117 | Nfkb1 | 1 | 1.3614 | 22.5392 |
| GO:0051783 | regulation of nuclear division | 1/9 | 143/29008 | 0.043508081 | 0.053604688 | 0.009447117 | Il1b | 1 | 1.3614 | 22.5392 |
| GO:0090316 | positive regulation of intracellular protein transport | 1/9 | 144/29008 | 0.043806312 | 0.053851383 | 0.009490594 | Il6 | 1 | 1.3585 | 22.3827 |
| GO:1904950 | negative regulation of establishment of protein localization | 1/9 | 144/29008 | 0.043806312 | 0.053851383 | 0.009490594 | Il1b | 1 | 1.3585 | 22.3827 |
| GO:0010469 | regulation of signaling receptor activity | 1/9 | 146/29008 | 0.044402525 | 0.054523324 | 0.009609015 | Tnf | 1 | 1.3526 | 22.0761 |
| GO:2001056 | positive regulation of cysteine-type endopeptidase activity | 1/9 | 148/29008 | 0.044998408 | 0.05519336 | 0.009727099 | Tnf | 1 | 1.3468 | 21.7778 |
| GO:0050921 | positive regulation of chemotaxis | 1/9 | 151/29008 | 0.045891613 | 0.056163567 | 0.009898086 | Il1b | 1 | 1.3383 | 21.3451 |
| GO:1900180 | regulation of protein localization to nucleus | 1/9 | 151/29008 | 0.045891613 | 0.056163567 | 0.009898086 | Il6 | 1 | 1.3383 | 21.3451 |
| GO:0002687 | positive regulation of leukocyte migration | 1/9 | 153/29008 | 0.04648667 | 0.056451769 | 0.009948877 | Il1b | 1 | 1.3327 | 21.0661 |
| GO:0006633 | fatty acid biosynthetic process | 1/9 | 153/29008 | 0.04648667 | 0.056451769 | 0.009948877 | Il1b | 1 | 1.3327 | 21.0661 |
| GO:0010565 | regulation of cellular ketone metabolic process | 1/9 | 153/29008 | 0.04648667 | 0.056451769 | 0.009948877 | Il1b | 1 | 1.3327 | 21.0661 |
| GO:0031099 | regeneration | 1/9 | 153/29008 | 0.04648667 | 0.056451769 | 0.009948877 | Il6 | 1 | 1.3327 | 21.0661 |
| GO:0032368 | regulation of lipid transport | 1/9 | 153/29008 | 0.04648667 | 0.056451769 | 0.009948877 | Il1b | 1 | 1.3327 | 21.0661 |
| GO:0050830 | defense response to Gram-positive bacterium | 1/9 | 153/29008 | 0.04648667 | 0.056451769 | 0.009948877 | Tnf | 1 | 1.3327 | 21.0661 |
| GO:1903900 | regulation of viral life cycle | 1/9 | 153/29008 | 0.04648667 | 0.056451769 | 0.009948877 | Tnf | 1 | 1.3327 | 21.0661 |
| GO:0035637 | multicellular organismal signaling | 1/9 | 155/29008 | 0.047081398 | 0.057110879 | 0.010065037 | Il6 | 1 | 1.3272 | 20.7943 |
| GO:0008360 | regulation of cell shape | 1/9 | 157/29008 | 0.047675796 | 0.057577692 | 0.010147306 | Il6 | 1 | 1.3217 | 20.5294 |
| GO:0071322 | cellular response to carbohydrate stimulus | 1/9 | 157/29008 | 0.047675796 | 0.057577692 | 0.010147306 | Nfkb1 | 1 | 1.3217 | 20.5294 |
| GO:0097530 | granulocyte migration | 1/9 | 157/29008 | 0.047675796 | 0.057577692 | 0.010147306 | Il1b | 1 | 1.3217 | 20.5294 |
| GO:2001235 | positive regulation of apoptotic signaling pathway | 1/9 | 157/29008 | 0.047675796 | 0.057577692 | 0.010147306 | Tnf | 1 | 1.3217 | 20.5294 |
| GO:0007613 | memory | 1/9 | 158/29008 | 0.047972871 | 0.05787287 | 0.010199328 | Il1b | 1 | 1.3190 | 20.3994 |
| GO:2000377 | regulation of reactive oxygen species metabolic process | 1/9 | 160/29008 | 0.048566774 | 0.058525093 | 0.010314273 | Tnf | 1 | 1.3137 | 20.1444 |
| GO:0050768 | negative regulation of neurogenesis | 1/9 | 162/29008 | 0.049160348 | 0.05917549 | 0.010428897 | Il1b | 1 | 1.3084 | 19.8957 |
| GO:0006865 | amino acid transport | 1/9 | 164/29008 | 0.049753593 | 0.059758687 | 0.010531678 | Il1b | 1 | 1.3032 | 19.6531 |
| GO:0010675 | regulation of cellular carbohydrate metabolic process | 1/9 | 164/29008 | 0.049753593 | 0.059758687 | 0.010531678 | Il6 | 1 | 1.3032 | 19.6531 |

**Table S6. Cellular components (CCs)**

| **ID** | **Description** | **Gene**  **Ratio** | **Bg**  **Ratio** | **pvalue** | **p.adjust** | **qvalue** | **geneID** | **Count** | **Enrichment**  **Score** | **Fold**  **Enrichment** |
| --- | --- | --- | --- | --- | --- | --- | --- | --- | --- | --- |
| GO:0005746 | mitochondrial respirasome | 3/9 | 77/28886 | 1.51223E-06 | 3.19439E-05 | 9.99665E-06 | Ndufb8/Sdhb/Uqcrc2 | 3 | 5.8204 | 125.0476 |
| GO:0098803 | respiratory chain complex | 3/9 | 82/28886 | 1.82937E-06 | 3.19439E-05 | 9.99665E-06 | Ndufb8/Sdhb/Uqcrc2 | 3 | 5.7377 | 117.4228 |
| GO:0070469 | respirasome | 3/9 | 92/28886 | 2.59004E-06 | 3.19439E-05 | 9.99665E-06 | Ndufb8/Sdhb/Uqcrc2 | 3 | 5.5867 | 104.6594 |
| GO:1990204 | oxidoreductase complex | 3/9 | 112/28886 | 4.68612E-06 | 4.33466E-05 | 1.35651E-05 | Ndufb8/Sdhb/Uqcrc2 | 3 | 5.3292 | 85.9702 |
| GO:0098800 | inner mitochondrial membrane protein complex | 3/9 | 130/28886 | 7.33503E-06 | 5.42792E-05 | 1.69864E-05 | Ndufb8/Sdhb/Uqcrc2 | 3 | 5.1346 | 74.0667 |
| GO:0098798 | mitochondrial protein-containing complex | 3/9 | 263/28886 | 6.01903E-05 | 0.000371173 | 0.000116157 | Ndufb8/Sdhb/Uqcrc2 | 3 | 4.2205 | 36.6109 |
| GO:0005743 | mitochondrial inner membrane | 3/9 | 456/28886 | 0.000305859 | 0.001616681 | 0.000505931 | Ndufb8/Sdhb/Uqcrc2 | 3 | 3.5145 | 21.1155 |
| GO:0019866 | organelle inner membrane | 3/9 | 500/28886 | 0.00040067 | 0.001853098 | 0.000579917 | Ndufb8/Sdhb/Uqcrc2 | 3 | 3.3972 | 19.2573 |
| GO:0005750 | mitochondrial respiratory chain complex III | 1/9 | 11/28886 | 0.003422523 | 0.012556941 | 0.003929626 | Uqcrc2 | 1 | 2.4657 | 291.7778 |
| GO:0045275 | respiratory chain complex III | 1/9 | 11/28886 | 0.003422523 | 0.012556941 | 0.003929626 | Uqcrc2 | 1 | 2.4657 | 291.7778 |
| GO:0005751 | mitochondrial respiratory chain complex IV | 1/9 | 12/28886 | 0.003733145 | 0.012556941 | 0.003929626 | Uqcrc2 | 1 | 2.4279 | 267.4630 |
| GO:1902495 | transmembrane transporter complex | 2/9 | 363/28886 | 0.005348108 | 0.016298529 | 0.005100536 | Ndufb8/Uqcrc2 | 2 | 2.2718 | 17.6835 |
| GO:1990351 | transporter complex | 2/9 | 376/28886 | 0.00572651 | 0.016298529 | 0.005100536 | Ndufb8/Uqcrc2 | 2 | 2.2421 | 17.0721 |
| GO:0001891 | phagocytic cup | 1/9 | 22/28886 | 0.00683463 | 0.016858755 | 0.005275855 | Tnf | 1 | 2.1653 | 145.8889 |
| GO:0045277 | respiratory chain complex IV | 1/9 | 22/28886 | 0.00683463 | 0.016858755 | 0.005275855 | Uqcrc2 | 1 | 2.1653 | 145.8889 |
| GO:0030141 | secretory granule | 2/9 | 428/28886 | 0.007360134 | 0.017020309 | 0.005326413 | Tnf/Il1b | 2 | 2.1331 | 14.9979 |
| GO:0070069 | cytochrome complex | 1/9 | 33/28886 | 0.010236349 | 0.022279112 | 0.006972126 | Uqcrc2 | 1 | 1.9899 | 97.2593 |
| GO:0009295 | nucleoid | 1/9 | 48/28886 | 0.014858365 | 0.025506145 | 0.007982008 | Tfam | 1 | 1.8280 | 66.8657 |
| GO:0042645 | mitochondrial nucleoid | 1/9 | 48/28886 | 0.014858365 | 0.025506145 | 0.007982008 | Tfam | 1 | 1.8280 | 66.8657 |
| GO:0005747 | mitochondrial respiratory chain complex I | 1/9 | 49/28886 | 0.015165816 | 0.025506145 | 0.007982008 | Ndufb8 | 1 | 1.8191 | 65.5011 |
| GO:0030964 | NADH dehydrogenase complex | 1/9 | 49/28886 | 0.015165816 | 0.025506145 | 0.007982008 | Ndufb8 | 1 | 1.8191 | 65.5011 |
| GO:0045271 | respiratory chain complex I | 1/9 | 49/28886 | 0.015165816 | 0.025506145 | 0.007982008 | Ndufb8 | 1 | 1.8191 | 65.5011 |
| GO:0019005 | SCF ubiquitin ligase complex | 1/9 | 65/28886 | 0.020073447 | 0.032292068 | 0.010105626 | Fbxo32 | 1 | 1.6974 | 49.3778 |
| GO:0030018 | Z disc | 1/9 | 130/28886 | 0.039787921 | 0.061339712 | 0.019195927 | Fbxo32 | 1 | 1.4002 | 24.6889 |
| GO:0031674 | I band | 1/9 | 143/28886 | 0.043688236 | 0.064658589 | 0.020234551 | Fbxo32 | 1 | 1.3596 | 22.4444 |
| GO:0055037 | recycling endosome | 1/9 | 158/28886 | 0.048171095 | 0.068551173 | 0.021452714 | Tnf | 1 | 1.3172 | 20.3136 |

**Table S7. Molecular functions (MFs)**

| **ID** | **Description** | **Gene**  **Ratio** | **Bg**  **Ratio** | **pvalue** | **p.adjust** | **qvalue** | **geneID** | **Count** | **Enrichment**  **Score** | **Fold**  **Enrichment** |
| --- | --- | --- | --- | --- | --- | --- | --- | --- | --- | --- |
| GO:0005125 | cytokine activity | 3/9 | 228/28438 | 4.12289E-05 | 0.000869357 | 0.000166384 | Tnf/Il6/Il1b | 3 | 4.3848 | 41.5760 |
| GO:0001223 | transcription coactivator binding | 2/9 | 35/28438 | 5.26883E-05 | 0.000869357 | 0.000166384 | Nfkb1/Tfam | 2 | 4.2783 | 180.5587 |
| GO:0005126 | cytokine receptor binding | 3/9 | 315/28438 | 0.000107624 | 0.001183863 | 0.000226577 | Tnf/Il6/Il1b | 3 | 3.9681 | 30.0931 |
| GO:0009055 | electron transfer activity | 2/9 | 70/28438 | 0.000212627 | 0.001754175 | 0.000335727 | Ndufb8/Sdhb | 2 | 3.6724 | 90.2794 |
| GO:0001221 | transcription coregulator binding | 2/9 | 90/28438 | 0.000351462 | 0.002279748 | 0.000436315 | Nfkb1/Tfam | 2 | 3.4541 | 70.2173 |
| GO:0048018 | receptor ligand activity | 3/9 | 498/28438 | 0.0004145 | 0.002279748 | 0.000436315 | Tnf/Il6/Il1b | 3 | 3.3825 | 19.0348 |
| GO:0031072 | heat shock protein binding | 2/9 | 147/28438 | 0.000932932 | 0.00416592 | 0.000797305 | Nfkb1/Tfam | 2 | 3.0301 | 42.9902 |
| GO:0070851 | growth factor receptor binding | 2/9 | 153/28438 | 0.00100992 | 0.00416592 | 0.000797305 | Il6/Il1b | 2 | 2.9957 | 41.3043 |
| GO:0005149 | interleukin-1 receptor binding | 1/9 | 17/28438 | 0.005368032 | 0.017714504 | 0.003390336 | Il1b | 1 | 2.2702 | 185.8693 |
| GO:0008301 | DNA binding, bending | 1/9 | 17/28438 | 0.005368032 | 0.017714504 | 0.003390336 | Tfam | 1 | 2.2702 | 185.8693 |
| GO:0008137 | NADH dehydrogenase (ubiquinone) activity | 1/9 | 22/28438 | 0.006941982 | 0.018039006 | 0.003452441 | Ndufb8 | 1 | 2.1585 | 143.6263 |
| GO:0050136 | NADH dehydrogenase (quinone) activity | 1/9 | 22/28438 | 0.006941982 | 0.018039006 | 0.003452441 | Ndufb8 | 1 | 2.1585 | 143.6263 |
| GO:0003954 | NADH dehydrogenase activity | 1/9 | 25/28438 | 0.007885289 | 0.018039006 | 0.003452441 | Ndufb8 | 1 | 2.1032 | 126.3911 |
| GO:0003955 | NAD(P)H dehydrogenase (quinone) activity | 1/9 | 25/28438 | 0.007885289 | 0.018039006 | 0.003452441 | Ndufb8 | 1 | 2.1032 | 126.3911 |
| GO:0051537 | 2 iron, 2 sulfur cluster binding | 1/9 | 26/28438 | 0.008199548 | 0.018039006 | 0.003452441 | Sdhb | 1 | 2.0862 | 121.5299 |
| GO:0005164 | tumor necrosis factor receptor binding | 1/9 | 33/28438 | 0.010396883 | 0.02144357 | 0.004104033 | Tnf | 1 | 1.9831 | 95.7508 |
| GO:0016655 | oxidoreductase activity, acting on NAD(P)H, quinone or similar compound as acceptor | 1/9 | 40/28438 | 0.012589889 | 0.021866649 | 0.004185005 | Ndufb8 | 1 | 1.9000 | 78.9944 |
| GO:0042805 | actinin binding | 1/9 | 40/28438 | 0.012589889 | 0.021866649 | 0.004185005 | Nfkb1 | 1 | 1.9000 | 78.9944 |
| GO:0051539 | 4 iron, 4 sulfur cluster binding | 1/9 | 40/28438 | 0.012589889 | 0.021866649 | 0.004185005 | Sdhb | 1 | 1.9000 | 78.9944 |
| GO:0032813 | tumor necrosis factor receptor superfamily binding | 1/9 | 48/28438 | 0.015090891 | 0.02469541 | 0.004726394 | Tnf | 1 | 1.8213 | 65.8287 |
| GO:0015453 | oxidoreduction-driven active transmembrane transporter activity | 1/9 | 50/28438 | 0.015715261 | 0.02469541 | 0.004726394 | Ndufb8 | 1 | 1.8037 | 63.1956 |
| GO:0016627 | oxidoreductase activity, acting on the CH-CH group of donors | 1/9 | 65/28438 | 0.020386833 | 0.0288863 | 0.005528478 | Sdhb | 1 | 1.6907 | 48.6120 |
| GO:0051536 | iron-sulfur cluster binding | 1/9 | 67/28438 | 0.021008218 | 0.0288863 | 0.005528478 | Sdhb | 1 | 1.6776 | 47.1609 |
| GO:0051540 | metal cluster binding | 1/9 | 67/28438 | 0.021008218 | 0.0288863 | 0.005528478 | Sdhb | 1 | 1.6776 | 47.1609 |
| GO:0016651 | oxidoreductase activity, acting on NAD(P)H | 1/9 | 75/28438 | 0.023490255 | 0.031007136 | 0.00593438 | Ndufb8 | 1 | 1.6291 | 42.1304 |
| GO:0005178 | integrin binding | 1/9 | 142/28438 | 0.044058727 | 0.055920692 | 0.010702525 | Il1b | 1 | 1.3560 | 22.2520 |
| GO:0008083 | growth factor activity | 1/9 | 148/28438 | 0.045881753 | 0.056077699 | 0.010732574 | Il6 | 1 | 1.3384 | 21.3498 |

**Table S8. KEGG pathways**

| **ID** | **Description** | **Gene**  **Ratio** | **Bg**  **Ratio** | **pvalue** | **p.adjust** | **qvalue** | **Gene**  **ID** | **Count** | **Enrichment**  **Score** | **Fold**  **Enrichment** |
| --- | --- | --- | --- | --- | --- | --- | --- | --- | --- | --- |
| mmu04932 | Non-alcoholic fatty liver disease | 7/9 | 162/10525 | 6.30785E-12 | 6.87556E-10 | 2.32395E-10 | Tnf/Il6/Il1b/Nfkb1  /Ndufb8/Sdhb/Uqcrc2 | 7 | 11.2001 | 50.5316 |
| mmu05010 | Alzheimer disease | 7/9 | 389/10525 | 3.01745E-09 | 1.64451E-07 | 5.55846E-08 | Tnf/Il6/Il1b/Nfkb1  /Ndufb8/Sdhb/Uqcrc2 | 7 | 8.5204 | 21.0440 |
| mmu01523 | Antifolate resistance | 4/9 | 29/10525 | 5.80084E-09 | 2.10764E-07 | 7.12384E-08 | Tnf/Il6/Il1b/Nfkb1 | 4 | 8.2365 | 161.3027 |
| mmu05022 | Pathways of neurodegeneration - multiple diseases | 7/9 | 481/10525 | 1.32632E-08 | 3.61423E-07 | 1.22161E-07 | Tnf/Il6/Il1b/Nfkb1  /Ndufb8/Sdhb/Uqcrc2 | 7 | 7.8774 | 17.0189 |
| mmu05020 | Prion disease | 6/9 | 273/10525 | 2.26918E-08 | 4.94682E-07 | 1.67203E-07 | Tnf/Il6/Il1b/Ndufb8  /Sdhb/Uqcrc2 | 6 | 7.6441 | 25.7021 |
| mmu05134 | Legionellosis | 4/9 | 60/10525 | 1.17699E-07 | 2.09502E-06 | 7.08117E-07 | Tnf/Il6/Il1b/Nfkb1 | 4 | 6.9292 | 77.9630 |
| mmu05321 | Inflammatory bowel disease | 4/9 | 62/10525 | 1.34542E-07 | 2.09502E-06 | 7.08117E-07 | Tnf/Il6/Il1b/Nfkb1 | 4 | 6.8711 | 75.4480 |
| mmu05133 | Pertussis | 4/9 | 77/10525 | 3.24522E-07 | 4.42161E-06 | 1.49451E-06 | Tnf/Il6/Il1b/Nfkb1 | 4 | 6.4888 | 60.7504 |
| mmu04657 | IL-17 signaling pathway | 4/9 | 94/10525 | 7.26548E-07 | 8.79931E-06 | 2.97417E-06 | Tnf/Il6/Il1b/Nfkb1 | 4 | 6.1387 | 49.7636 |
| mmu04933 | AGE-RAGE signaling pathway in diabetic complications | 4/9 | 101/10525 | 9.70162E-07 | 9.91289E-06 | 3.35057E-06 | Tnf/Il6/Il1b/Nfkb1 | 4 | 6.0132 | 46.3146 |
| mmu05142 | Chagas disease | 4/9 | 103/10525 | 1.04976E-06 | 9.91289E-06 | 3.35057E-06 | Tnf/Il6/Il1b/Nfkb1 | 4 | 5.9789 | 45.4153 |
| mmu04620 | Toll-like receptor signaling pathway | 4/9 | 104/10525 | 1.09133E-06 | 9.91289E-06 | 3.35057E-06 | Tnf/Il6/Il1b/Nfkb1 | 4 | 5.9620 | 44.9786 |
| mmu05146 | Amoebiasis | 4/9 | 108/10525 | 1.26999E-06 | 1.06484E-05 | 3.59917E-06 | Tnf/Il6/Il1b/Nfkb1 | 4 | 5.8962 | 43.3128 |
| mmu04625 | C-type lectin receptor signaling pathway | 4/9 | 112/10525 | 1.46958E-06 | 1.14417E-05 | 3.86732E-06 | Tnf/Il6/Il1b/Nfkb1 | 4 | 5.8328 | 41.7659 |
| mmu04668 | TNF signaling pathway | 4/9 | 118/10525 | 1.81159E-06 | 1.31642E-05 | 4.44951E-06 | Tnf/Il6/Il1b/Nfkb1 | 4 | 5.7419 | 39.6422 |
| mmu05135 | Yersinia infection | 4/9 | 136/10525 | 3.19646E-06 | 2.17759E-05 | 7.36026E-06 | Tnf/Il6/Il1b/Nfkb1 | 4 | 5.4953 | 34.3954 |
| mmu04936 | Alcoholic liver disease | 4/9 | 141/10525 | 3.69188E-06 | 2.35639E-05 | 7.96462E-06 | Tnf/Il6/Il1b/Nfkb1 | 4 | 5.4328 | 33.1757 |
| mmu05143 | African trypanosomiasis | 3/9 | 39/10525 | 3.89129E-06 | 2.35639E-05 | 7.96462E-06 | Tnf/Il6/Il1b | 3 | 5.4099 | 89.9573 |
| mmu05164 | Influenza A | 4/9 | 174/10525 | 8.52361E-06 | 4.88986E-05 | 1.65278E-05 | Tnf/Il6/Il1b/Nfkb1 | 4 | 5.0694 | 26.8838 |
| mmu05152 | Tuberculosis | 4/9 | 180/10525 | 9.75036E-06 | 5.31395E-05 | 1.79612E-05 | Tnf/Il6/Il1b/Nfkb1 | 4 | 5.0110 | 25.9877 |
| mmu05332 | Graft-versus-host disease | 3/9 | 60/10525 | 1.44399E-05 | 7.495E-05 | 2.53332E-05 | Tnf/Il6/Il1b | 3 | 4.8404 | 58.4722 |
| mmu05168 | Herpes simplex virus 1 infection | 4/9 | 213/10525 | 1.89759E-05 | 8.90365E-05 | 3.00944E-05 | Tnf/Il6/Il1b/Nfkb1 | 4 | 4.7218 | 21.9614 |
| mmu04621 | NOD-like receptor signaling pathway | 4/9 | 216/10525 | 2.00525E-05 | 8.90365E-05 | 3.00944E-05 | Tnf/Il6/Il1b/Nfkb1 | 4 | 4.6978 | 21.6564 |
| mmu05415 | Diabetic cardiomyopathy | 4/9 | 217/10525 | 2.04212E-05 | 8.90365E-05 | 3.00944E-05 | Nfkb1/Ndufb8  /Sdhb/Uqcrc2 | 4 | 4.6899 | 21.5566 |
| mmu05417 | Lipid and atherosclerosis | 4/9 | 217/10525 | 2.04212E-05 | 8.90365E-05 | 3.00944E-05 | Tnf/Il6/Il1b/Nfkb1 | 4 | 4.6899 | 21.5566 |
| mmu05140 | Leishmaniasis | 3/9 | 70/10525 | 2.29999E-05 | 9.64227E-05 | 3.2591E-05 | Tnf/Il1b/Nfkb1 | 3 | 4.6383 | 50.1190 |
| mmu05208 | Chemical carcinogenesis - reactive oxygen species | 4/9 | 229/10525 | 2.52471E-05 | 0.000101923 | 3.44502E-05 | Nfkb1/Ndufb8  /Sdhb/Uqcrc2 | 4 | 4.5978 | 20.4270 |
| mmu05144 | Malaria | 3/9 | 77/10525 | 3.0643E-05 | 0.000119289 | 4.03197E-05 | Tnf/Il6/Il1b | 3 | 4.5137 | 45.5628 |
| mmu04623 | Cytosolic DNA-sensing pathway | 3/9 | 82/10525 | 3.70188E-05 | 0.00013549 | 4.57957E-05 | Il6/Il1b/Nfkb1 | 3 | 4.4316 | 42.7846 |
| mmu05132 | Salmonella infection | 4/9 | 253/10525 | 3.73609E-05 | 0.00013549 | 4.57957E-05 | Tnf/Il6/Il1b/Nfkb1 | 4 | 4.4276 | 18.4892 |
| mmu05163 | Human cytomegalovirus infection | 4/9 | 255/10525 | 3.85338E-05 | 0.00013549 | 4.57957E-05 | Tnf/Il6/Il1b/Nfkb1 | 4 | 4.4142 | 18.3442 |
| mmu05323 | Rheumatoid arthritis | 3/9 | 87/10525 | 4.42117E-05 | 0.000150596 | 5.09017E-05 | Tnf/Il6/Il1b | 3 | 4.3545 | 40.3257 |
| mmu04640 | Hematopoietic cell lineage | 3/9 | 94/10525 | 5.57434E-05 | 0.000184122 | 6.22335E-05 | Tnf/Il6/Il1b | 3 | 4.2538 | 37.3227 |
| mmu04659 | Th17 cell differentiation | 3/9 | 105/10525 | 7.75893E-05 | 0.000248526 | 8.4002E-05 | Il6/Il1b/Nfkb1 | 3 | 4.1102 | 33.4127 |
| mmu05016 | Huntington disease | 4/9 | 308/10525 | 8.0677E-05 | 0.000248526 | 8.4002E-05 | Tfam/Ndufb8  /Sdhb/Uqcrc2 | 4 | 4.0933 | 15.1876 |
| mmu04064 | NF-kappa B signaling pathway | 3/9 | 107/10525 | 8.2082E-05 | 0.000248526 | 8.4002E-05 | Tnf/Il1b/Nfkb1 | 3 | 4.0858 | 32.7882 |
| mmu04931 | Insulin resistance | 3/9 | 110/10525 | 8.91357E-05 | 0.000262589 | 8.87553E-05 | Tnf/Il6/Nfkb1 | 3 | 4.0499 | 31.8939 |
| mmu05171 | Coronavirus disease - COVID-19 | 4/9 | 334/10525 | 0.000110615 | 0.000317291 | 0.000107245 | Tnf/Il6/Il1b/Nfkb1 | 4 | 3.9562 | 14.0053 |
| mmu04380 | Osteoclast differentiation | 3/9 | 136/10525 | 0.000167467 | 0.000468048 | 0.000158201 | Tnf/Il1b/Nfkb1 | 3 | 3.7761 | 25.7966 |
| mmu05014 | Amyotrophic lateral sclerosis | 4/9 | 376/10525 | 0.000175132 | 0.000477235 | 0.000161306 | Tnf/Ndufb8  /Sdhb/Uqcrc2 | 4 | 3.7566 | 12.4409 |
| mmu00190 | Oxidative phosphorylation | 3/9 | 141/10525 | 0.000186371 | 0.000495474 | 0.000167471 | Ndufb8/Sdhb/Uqcrc2 | 3 | 3.7296 | 24.8818 |
| mmu05162 | Measles | 3/9 | 145/10525 | 0.000202458 | 0.000525427 | 0.000177595 | Il6/Il1b/Nfkb1 | 3 | 3.6937 | 24.1954 |
| mmu05418 | Fluid shear stress and atherosclerosis | 3/9 | 149/10525 | 0.000219425 | 0.000556216 | 0.000188002 | Tnf/Il1b/Nfkb1 | 3 | 3.6587 | 23.5459 |
| mmu05161 | Hepatitis B | 3/9 | 164/10525 | 0.000291246 | 0.000721495 | 0.000243866 | Tnf/Il6/Nfkb1 | 3 | 3.5357 | 21.3923 |
| mmu05169 | Epstein-Barr virus infection | 3/9 | 228/10525 | 0.000765216 | 0.001853524 | 0.000626493 | Tnf/Il6/Nfkb1 | 3 | 3.1162 | 15.3874 |
| mmu04714 | Thermogenesis | 3/9 | 238/10525 | 0.000867111 | 0.002054675 | 0.000694482 | Ndufb8/Sdhb/Uqcrc2 | 3 | 3.0619 | 14.7409 |
| mmu05166 | Human T-cell leukemia virus 1 infection | 3/9 | 247/10525 | 0.000965927 | 0.002240129 | 0.000757166 | Tnf/Il6/Nfkb1 | 3 | 3.0151 | 14.2038 |
| mmu05012 | Parkinson disease | 3/9 | 274/10525 | 0.001304823 | 0.002963035 | 0.001001509 | Ndufb8/Sdhb/Uqcrc2 | 3 | 2.8844 | 12.8041 |
| mmu04940 | Type I diabetes mellitus | 2/9 | 67/10525 | 0.001396338 | 0.003106139 | 0.001049878 | Tnf/Il1b | 2 | 2.8550 | 34.9088 |
| mmu04060 | Cytokine-cytokine receptor interaction | 3/9 | 294/10525 | 0.001599199 | 0.003442347 | 0.001163517 | Tnf/Il6/Il1b | 3 | 2.7961 | 11.9331 |
| mmu04920 | Adipocytokine signaling pathway | 2/9 | 72/10525 | 0.001610639 | 0.003442347 | 0.001163517 | Tnf/Nfkb1 | 2 | 2.7930 | 32.4846 |
| mmu04622 | RIG-I-like receptor signaling pathway | 2/9 | 73/10525 | 0.001655274 | 0.003452688 | 0.001167012 | Tnf/Nfkb1 | 2 | 2.7811 | 32.0396 |
| mmu04010 | MAPK signaling pathway | 3/9 | 299/10525 | 0.00167883 | 0.003452688 | 0.001167012 | Tnf/Il1b/Nfkb1 | 3 | 2.7750 | 11.7336 |
| mmu04061 | Viral protein interaction with cytokine and cytokine receptor | 2/9 | 95/10525 | 0.002784984 | 0.005621542 | 0.001900087 | Tnf/Il6 | 2 | 2.5552 | 24.6199 |
| mmu05410 | Hypertrophic cardiomyopathy | 2/9 | 100/10525 | 0.003080647 | 0.006105281 | 0.002063591 | Tnf/Il6 | 2 | 2.5114 | 23.3889 |
| mmu05145 | Toxoplasmosis | 2/9 | 109/10525 | 0.003648552 | 0.007101645 | 0.002400363 | Tnf/Nfkb1 | 2 | 2.4379 | 21.4577 |
| mmu04066 | HIF-1 signaling pathway | 2/9 | 117/10525 | 0.004191509 | 0.008015342 | 0.002709193 | Il6/Nfkb1 | 2 | 2.3776 | 19.9905 |
| mmu04660 | T cell receptor signaling pathway | 2/9 | 122/10525 | 0.004548905 | 0.008548804 | 0.002889504 | Tnf/Nfkb1 | 2 | 2.3421 | 19.1712 |
| mmu04071 | Sphingolipid signaling pathway | 2/9 | 126/10525 | 0.004844737 | 0.008950447 | 0.00302526 | Tnf/Nfkb1 | 2 | 2.3147 | 18.5626 |
| mmu04068 | FoxO signaling pathway | 2/9 | 133/10525 | 0.005383494 | 0.009780014 | 0.003305654 | Fbxo32/Il6 | 2 | 2.2689 | 17.5856 |
| mmu04210 | Apoptosis | 2/9 | 136/10525 | 0.005622536 | 0.010046827 | 0.003395837 | Tnf/Nfkb1 | 2 | 2.2501 | 17.1977 |
| mmu05160 | Hepatitis C | 2/9 | 167/10525 | 0.008373347 | 0.014720884 | 0.004975673 | Tnf/Nfkb1 | 2 | 2.0771 | 14.0053 |
| mmu04217 | Necroptosis | 2/9 | 177/10525 | 0.00936762 | 0.01620747 | 0.00547814 | Tnf/Il1b | 2 | 2.0284 | 13.2141 |
| mmu04218 | Cellular senescence | 2/9 | 183/10525 | 0.009988654 | 0.017011926 | 0.005750048 | Il6/Nfkb1 | 2 | 2.0005 | 12.7808 |
| mmu05167 | Kaposi sarcoma-associated herpesvirus infection | 2/9 | 224/10525 | 0.014710118 | 0.024667736 | 0.008337719 | Il6/Nfkb1 | 2 | 1.8324 | 10.4415 |
| mmu05202 | Transcriptional misregulation in cancer | 2/9 | 226/10525 | 0.014961245 | 0.024708722 | 0.008351572 | Il6/Nfkb1 | 2 | 1.8250 | 10.3491 |
| mmu05170 | Human immunodeficiency virus 1 infection | 2/9 | 242/10525 | 0.017037927 | 0.027718419 | 0.009368852 | Tnf/Nfkb1 | 2 | 1.7686 | 9.6648 |
| mmu05310 | Asthma | 1/9 | 25/10525 | 0.021183656 | 0.033956155 | 0.011477213 | Tnf | 1 | 1.6740 | 46.7778 |
| mmu00020 | Citrate cycle (TCA cycle) | 1/9 | 32/10525 | 0.027043144 | 0.042720329 | 0.014439513 | Sdhb | 1 | 1.5679 | 36.5451 |
| mmu05165 | Human papillomavirus infection | 2/9 | 359/10525 | 0.035639409 | 0.055495651 | 0.018757584 | Tnf/Nfkb1 | 2 | 1.4481 | 6.5150 |
| mmu04672 | Intestinal immune network for IgA production | 1/9 | 43/10525 | 0.03618793 | 0.055556118 | 0.018778022 | Il6 | 1 | 1.4414 | 27.1964 |
| mmu04151 | PI3K-Akt signaling pathway | 2/9 | 368/10525 | 0.037301371 | 0.056470131 | 0.019086959 | Il6/Nfkb1 | 2 | 1.4283 | 6.3557 |
| mmu04930 | Type II diabetes mellitus | 1/9 | 48/10525 | 0.040319334 | 0.05938929 | 0.020073637 | Tnf | 1 | 1.3945 | 24.3634 |
| mmu05030 | Cocaine addiction | 1/9 | 48/10525 | 0.040319334 | 0.05938929 | 0.020073637 | Nfkb1 | 1 | 1.3945 | 24.3634 |

**Table S9. GO chord plot**

| **Description** | **Fbxo32** | **TRIM63** | **Tnfα** | **Il6** | **Il1β** | **Nfkb1** | **Tfam** | **Ndufb8** | **Sdhb** | **Uqcrc2** | **Mtco1** | **Atp5f1a** |
| --- | --- | --- | --- | --- | --- | --- | --- | --- | --- | --- | --- | --- |
| AGE-RAGE signaling pathway in diabetic complications | 0 | 0 | 1 | 1 | 1 | 1 | 0 | 0 | 0 | 0 | 0 | 0 |
| Oxidative phosphorylation | 0 | 0 | 0 | 0 | 0 | 0 | 0 | 1 | 1 | 1 | 0 | 0 |
| IL-17 signaling pathway | 0 | 0 | 1 | 1 | 1 | 1 | 0 | 0 | 0 | 0 | 0 | 0 |
| Pathways of neurodegeneration - multiple diseases | 0 | 0 | 1 | 1 | 1 | 1 | 0 | 1 | 1 | 1 | 0 | 0 |
| Chemical carcinogenesis - reactive oxygen species | 0 | 0 | 0 | 0 | 0 | 1 | 0 | 1 | 1 | 1 | 0 | 0 |
| MAPK signaling pathway | 0 | 0 | 1 | 0 | 1 | 1 | 0 | 0 | 0 | 0 | 0 | 0 |
| Huntington disease | 0 | 0 | 0 | 0 | 0 | 0 | 1 | 1 | 1 | 1 | 0 | 0 |
| Parkinson disease | 0 | 0 | 0 | 0 | 0 | 0 | 0 | 1 | 1 | 1 | 0 | 0 |
| log2FC | -1.5825 | -1.7153 | -1.8009 | -0.5309 | -0.6541 | -0.3755 | 0.6757 | 0.4437 | 0.1493 | 0.1898 | 0.2458 | 0.1862 |

**
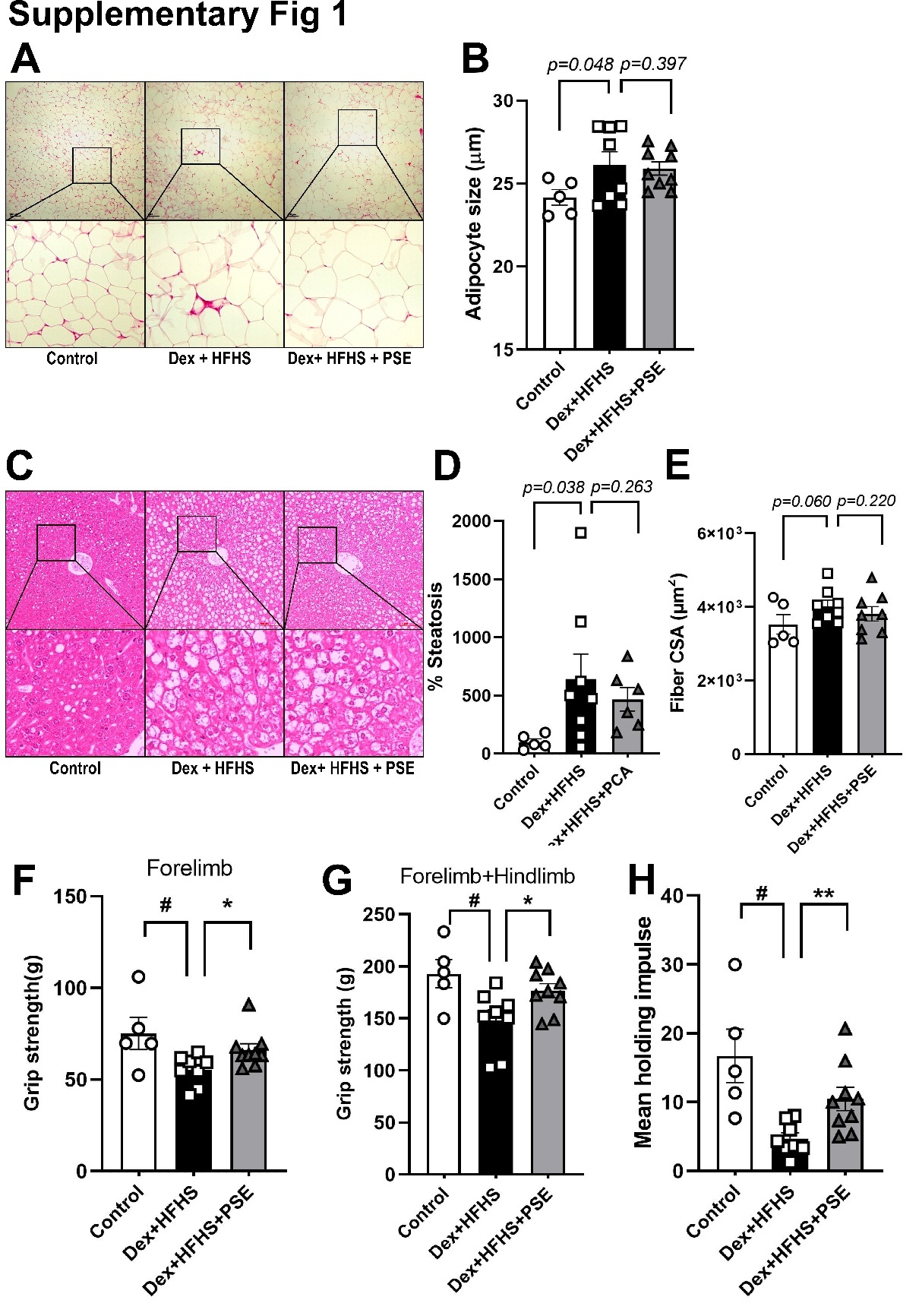
**

**Supplementary Fig. 1.** Male C57BL/6 mice are fed control diet (Control, n=5) or HFHS diet with daily administration of saline or PSE (10 mg/kg body weight [BW]) for 10 weeks. Dex was injected during the last six days to induce muscle atrophy (Dex+HFHS, n=8; Dex+HFHS+PSE, n=9 per group). **(A)** Representative sections of the epididymal adipose tissue stained with hematoxylin and eosin (H&E) are examined at a 20x magnification. Scale bars measuring 100 µm are included to provide a visual reference for the size of the adipocytes. **(B)** adipocytes size (µm). **(C)** Representative sections of the liver tissue stained with hematoxylin and eosin (H&E) are examined at a 20x magnification. (**D)** % Steatosis measured by MIPAR software. **(E)** Muscle fiber CSA (µm^2^). **(F)** Grip strength test of forelimbs (g). **(G)** Grip strength test of forelimbs + hindlimbs (g). **(H)** Mean holding impulse. Data are expressed as the mean ± standard error of the mean (SEM); *n.s.* not significant; #p<0.05 (Control vs. Dex+HFHS), *p<0.0, **p<0.01 (Dex+HFHS vs. PSE+Dex+HFHS) by Student’s t-test.

**Supplementary Fig 2**

**
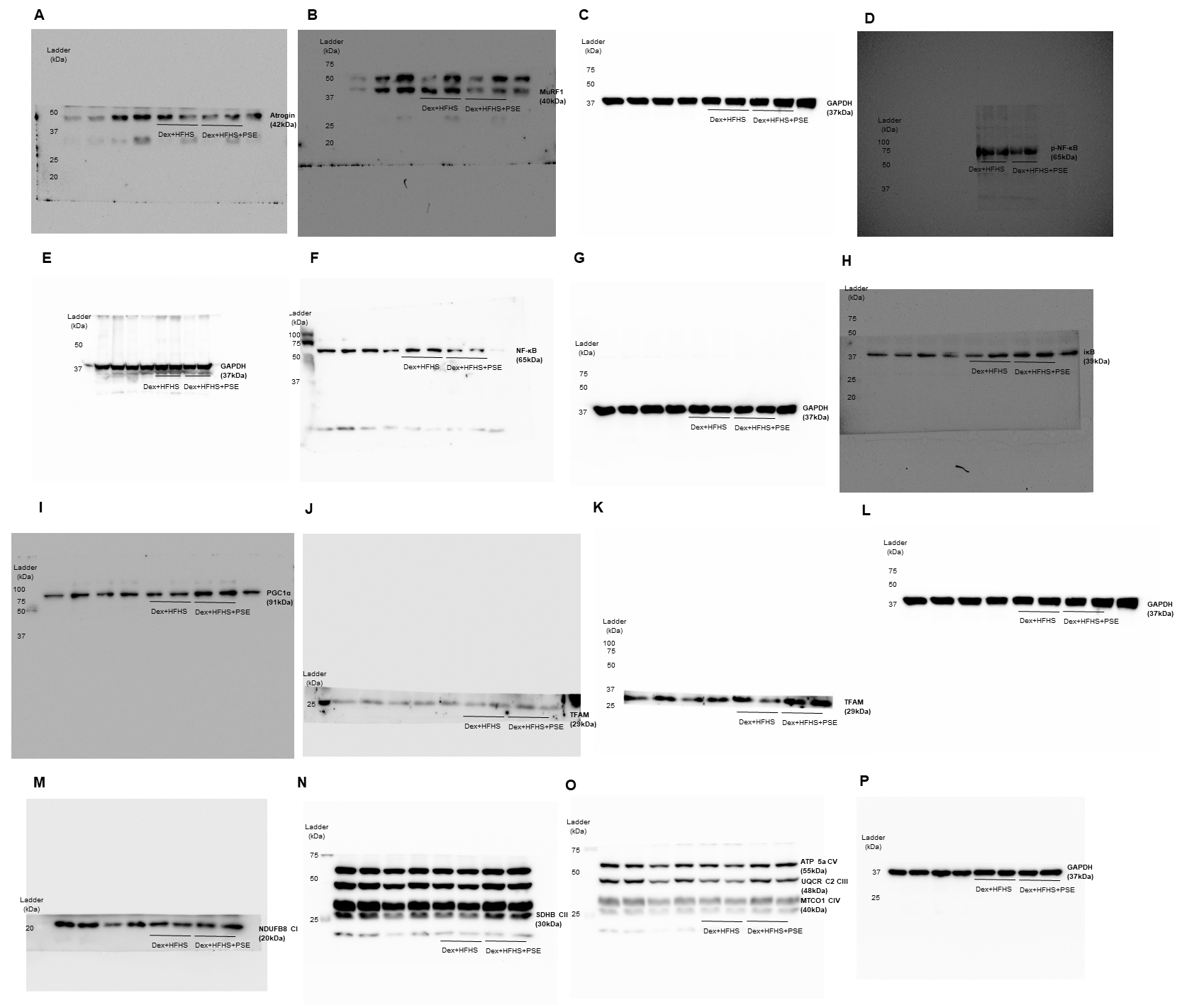
**

**Supplementary Fig. 2.** Original western blot of Figure 2B, 2E, 3A, and 3B with group name and ladder. Examples of the whole western blot with the same time exposure to all analyzed proteins. Each part of blots is separately presented with time exposure specific for each protein. (**A**) Western blot analysis of Figure 2B for the atrophic protein Atrogin-1, (**B**) Western blot analysis of Figure 2B for MuRF1. (**C**) Western blot analysis of Figure 2B for GAPDH, (**D**) Western blot analysis of Figure 2E for p-NF-κB, (**E**) Western blot analysis of Figure 2E for GAPDH, (**F**) Western blot analysis of Figure 2E for NF-κB, (**G**) Western blot analysis of Figure 2E for GAPDH, (**H**) Western blot analysis of Figure 2E for iκB, (**I**) Western blot analysis of Figure 3A for PGC1α, (**J**) Western blot analysis of Figure 3A for TFAM (before mask), (**K**) Western blot analysis of Figure 3A for TFAM (after mask), (**L**) Western blot analysis of Figure 3A for GAPDH, (**M**) Western blot analysis of Figure 3B for OXPHOS subunit, (NDUFB8) CI, (**N**) Western blot analysis of Figure 3B for OXPHOS subunit, (SDHB) CII, (**O**) Western blot analysis of Figure 3B for OXPHOS subunits, (UQCR C2) CIII, (MTCO1) CIV, (ATP5a) CV, (**P**) Western blot analysis of Figure 3B for GAPDH.

**Supplementary Fig 3**


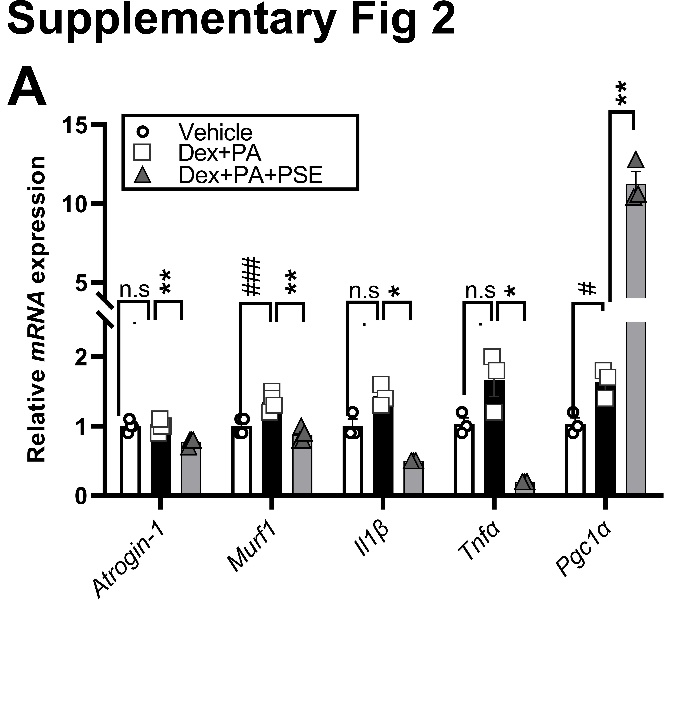


**Supplementary Fig. 3.** The C2C12 cells were incubated with 25 µg/mL of PSE for five days during and after differentiation. After starving for 1 h, they are treated with 10 µM Dex+0.75 mM of PA and harvested after 24 h. (**A**) Atrophy and inflammatory gene expressions of *Atrogin-1, Murf1, Il1β, Tnfα*, and *Pgc1α* are quantified using real-time PCR. Data are expressed as the mean ± standard error of the mean (SEM); *n.s.* not significant; #p<0.05, ###p<0.001 (Vehicle vs. Dex+PA), *p<0.05, **p<0.01, (Dex+PA vs. Dex+PA+PSE) by Student’s t-test.
